# Cyclic Ether Triggers for Polymeric Frustrated Lewis Pair Gels

**DOI:** 10.1021/jacs.1c06408

**Published:** 2021-08-13

**Authors:** Utku Yolsal, Thomas A. R. Horton, Meng Wang, Michael P. Shaver

**Affiliations:** † Department of Materials, School of Natural Sciences, University of Manchester, Oxford Road, Manchester, M1 3BB, United Kingdom; ‡ Sustainable Materials Innovation Hub, Henry Royce Institute, University of Manchester, Oxford Road, Manchester, M13 9BL, United Kingdom

## Abstract

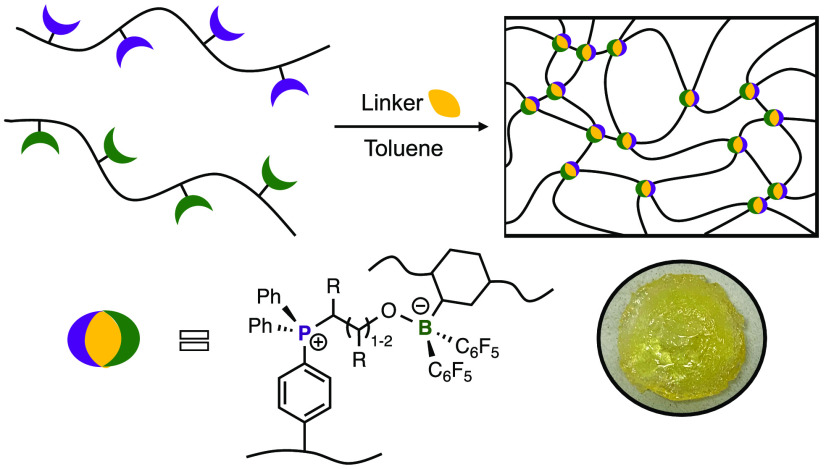

Sterically hindered Lewis acid and base centers are unable to form Lewis adducts, instead forming frustrated Lewis pairs (FLPs), where latent reactivity can be utilized for the activation of small molecules. Applying FLP chemistry into polymeric frameworks transforms this chemistry into responsive and functional materials. Here, we report a versatile synthesis strategy for the preparation of macromolecular FLPs and explore its potential with the ring-opening reactions of cyclic ethers. Addition of the cyclic substrates triggered polymer network formation, where the extent of cross-linking, strength of network, and reactivity are tuned by the steric and electronic properties of the ethers. The resultant networks behave like covalently cross-linked polymers, demonstrating the versatility of FLPs to simultaneously tune both small-molecule capture and mechanical properties of materials.

Bulky substituents can preclude the close contact required to form thermodynamically favorable bonds. When applied to Lewis acids (LAs) and bases (LBs), frustrated Lewis pairs (FLPs) are formed (Figure 1A), promoting activation of small molecules and subsequent applications in catalysis.^1−5^ Incorporating FLPs into polymer frameworks has the profound potential to extend this concept into responsive materials, as the dynamic nature of these bonds translates into functionality. The first-ever such macromolecular FLPs, capable of forming self-healing polymer networks via small-molecule activation, showed tunable rheological responses.^6−8^ Increased Lewis acidity in macromolecular FLPs can activate CO_2_ and facilitate catalytic formylations,^9^ while intramolecular and partially macromolecular FLPs promote C–H activation and amine formylation.^10,11^

**Figure 1:**
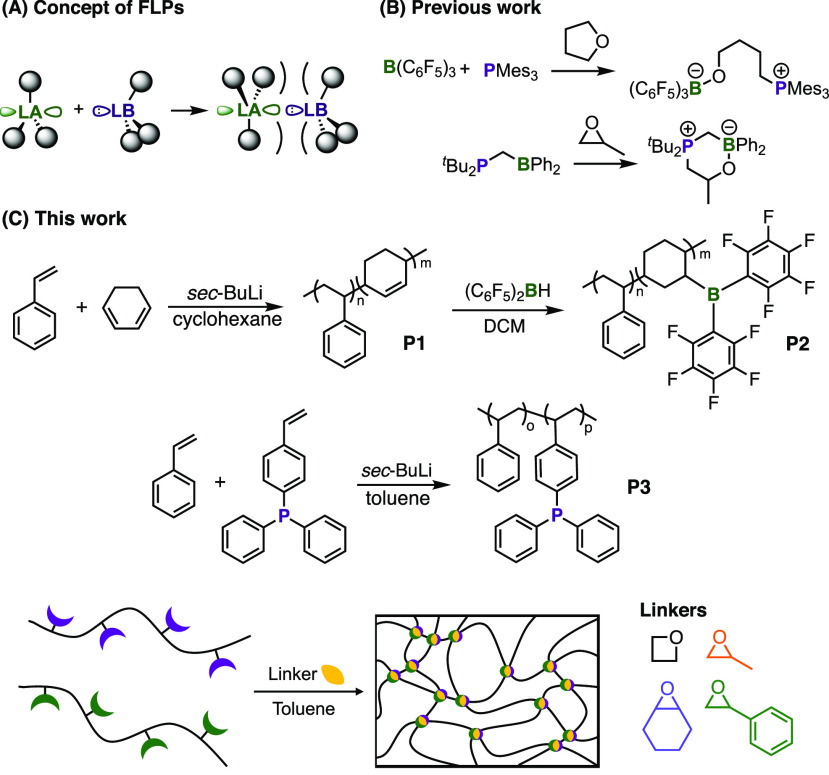
(A) Formation of FLPs. (B) Selected previously reported reactions between cyclic ethers and FLPs.^13,18^ (C) Schematic representation of the preparation of poly(FLP) networks using cyclic ether substrates.

Despite these exciting advances, incorporation of FLPs within polymer networks remains largely unexplored. This limited scope of reactivity is, in part, driven by the synthetic challenge of the air-sensitive polymers themselves. New polymeric systems that unlock a broader range of reactivity could thus significantly expand this nascent field. The FLP-mediated ring-openings of cyclic substrates (Figure 1B) are of particular interest; activation of these molecules has been reported for small-molecule LA/LB pairs for tetrahydrofuran, dioxane, and 1,4-thioxane ethers.^12−17^ In general, the activation of these cyclic substrates proceeds via heteroatom coordination to the Lewis acidic center, followed by attack of a Lewis basic species to form a zwitterionic product. FLP-mediated ring-openings of three- and four-membered cyclic ethers are rare. In 2018, Slootweg et al. showed that monosubstituted epoxides undergo regioselective FLP-mediated ring-opening reactions forming zwitterionic species that were stable at high temperatures.^18^ The inherent toxicity of many epoxides and the robustness of the resultant small molecules sparked interest in the capture of these substrates by poly(FLP)s (Figure 1C).

To develop this chemistry we recognized the need to rethink the synthesis strategy for preparing poly(FLP)s. Functionality can be incorporated into polymer chains using two main approaches: postpolymerization modification or polymerization of a functional monomer.^19,20^ While Si–B exchange reactions are a potentially scalable strategy,^21−23^ the majority of previous efforts to prepare air-sensitive LA-containing polymers through either of these methods has required lengthy and challenging syntheses that prevented nonexpert adoption. However, hydroboration reactions are a facile route to organoboranes; although previously applied to build polymer chains,^24,25^ their applications for postpolymerization modifications are rare. We felt that the residual alkene groups in the backbones of butadiene or cyclohexadiene (co)polymers could provide the framework for synthetically accessible, scalable, polymeric Lewis acids.^26^

This paper thus reports the development of a new, accessible poly(FLP) system and explores the capture and ring-opening of 1,3-propylene oxide (oxetane, **L1**), 1,2-propylene oxide (**L2**), styrene oxide (**L3**), and cyclohexene oxide (**L4**) to form responsive, functional poly(FLP) networks (Figure 1C). Unlike the diethyl azodicarboxylate triggered poly(FLP) gels,^6,8^ which behaved as supramolecular assemblies, we show that these poly(FLP) networks behave as covalently cross-linked chemical networks. A change in these linkers thus directly impacts the mechanical properties of the resulting polymeric networks, revealing the versatility of FLPs to simultaneously tune both reactivity and function.

To meet the steric bulk requirement of FLPs, 1,3-cyclohexadiene (1,3-CHD) was copolymerized with styrene (Figure 1C, feed ratio 1:9), resulting in the formation of copolymer **P1** with sterically encumbered cyclohexene units (Supporting Information section SYN1 and Table S1).^27^ Treatment with bis(pentafluorophenyl)borane, a highly Lewis acidic hydroborating agent prepared in a single step from commercially available starting materials,^28^ gave the desired organoborane macromolecules (Supporting Information section SYN2). This work presents one of the rare examples of alkylborane-based polymeric LAs which have long been considered prone to degradation via retro-hydroboration.^29^ However, the presence of neighboring styrene units flanking 1,3-CHD repeat units can create π–π interactions with the C_6_F_5_ groups that inhibit boryl migration.^28,30−32^

While hydroboration could not be monitored by ^11^B NMR spectroscopy due to signal broadening,^33,34^ complete conversion of (C_6_F_5_)_2_BH was confirmed by ^1^H and ^19^F NMR spectra (Figures S2 and S3), with the disappearance of CHD olefin peaks and broadening of the aryl fluorine resonances. A concomitant increase in polymer *M*_n_ (**P2**), observed by gel permeation chromatography, correlated to the addition of boron moieties (Table S1). The Lewis acidity matched that of a small-molecule mimic, as confirmed by a ^31^P NMR study using the Gutmann–Beckett method (acceptor number = 68 vs 69 for CyB(C_6_F_5_)_2_, **1**; see Supporting Information sections SYN4 and SYN5).^35,36^ Line width (ω_1/2_) of the ^31^P resonances also increased from 50 (**1**) to 230 Hz (**P2**), further confirming the polymer-supported nature of the LAs. While this paper focused solely on this highly Lewis acidic variant, the ease of synthesis and large variety of commercially available hydroborating agents suggest this is a flexible synthetic route to tunable polymeric LAs.

To prepare the LB polymer, 4-styryldiphenylphosphine was copolymerized with styrene using anionic polymerization. In earlier poly(FLP) publications, blocky polymers have been reported using reversible-addition–fragmentation-chain-transfer (RAFT) copolymerization,^6,9^ meaning that functional monomers were not distributed along a polymer chain. Anionic copolymerization eliminates the presence of any donating RAFT agents on the polymer chains. Unwanted interactions with the Lewis acidic borane units can therefore be minimized while maintaining a well-controlled polymerization (Table S1) and potentially afford a more random copolymerization and thus increase potential sites for capture and cross-linking.

Reactivity was modeled using zwitterionic small molecules to probe cross-linking reaction, using CyB(C_6_F_5_)_2_ (**1**) and PPh_3_ (**2**). Complexes of the linker molecules were formed with **1** and **2**, forming **SM1**, **SM2**, **SM3**, and **SM4** (Scheme 1). The mimics were characterized using NMR spectroscopy (Table S2, Figures S8–S23) with tetracoordinate borate centers confirmed by upfield shifts in ^11^B NMR spectra and tetracoordinate phosphonium ions observed in downfield-shifted ^31^P NMR peaks. *para*-Fluorine atoms additionally showed the large upfield shifts common for a tetracoordinate boron.^37^ For **SM3** and **SM4**, two sets of fluorine environments were observed corresponding to the diastereotopic C_6_F_5_ signals. The selectivity of the ring-opening was probed through 2D NMR studies and ^13^C–^31^P coupling constants which revealed that **2** attacks at the less sterically hindered carbon for **SM2** (47 Hz) and at the more hindered carbon for **SM3** (44 Hz). The latter was attributed to the stabilizing inductive forces of the phenyl ring (see the Supporting Information).

**Scheme 1:**
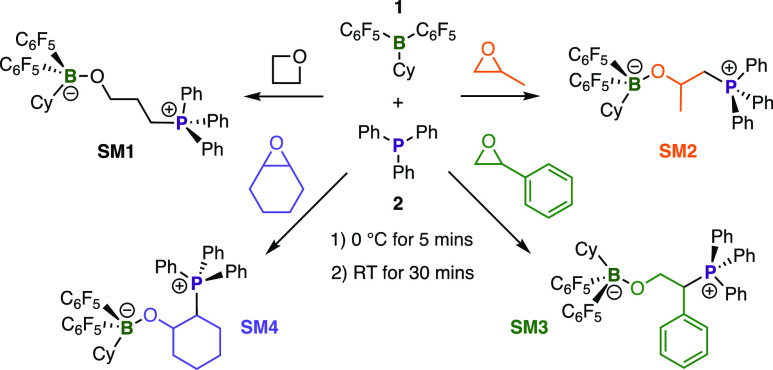
FLP-Mediated Ring-Opening Reactions of **L1**, **L2**, **L3**, and **L4** Using 1/2 Pair

Encouraged by these FLP-mediated ring-openings, we explored the reactivity of **L1**, **L2**, **L3**, and **L4** as triggers for polymeric networks using **P2** and **P3**. Two separate solutions of these polymers were prepared in toluene with an equivalent number of B/P units. The frustrated nature of their structure was confirmed, with no change in solution viscosity observed upon mixing. The addition of cyclic substrates (2.5 equiv) triggered immediate gelation (Movies S1 and S2, Figure S30), attributed to the capture and poly(FLP)-mediated ring-opening of the substrates (Figure 2A). Although coordination of the cyclic ether substrate to **P2** may be expected to disrupt the π–π interactions to induce retro-hydroboration, the obtained gels were stable hinting the rapid and efficient formation of the borate groups. Unlike our first generation poly(FLP) networks,^6,8^ no chain rearrangement was visually observed. Gel fractions and swelling capacities of the samples were determined (Figures S31 and S32). The formation of **N1** gave a gel fraction of ∼1, suggesting all macromolecules react to form a continuous network structure. The gel fraction then decreased from **N1** to **N2**, **N3**, and **N4**. As expected, the reverse trend, peaking at a swelling ratio of 16 for **N3** and **N4** compared to that of the tighter gel **N1** (5). As both solvent and temperature were controlled during gelation, this swelling ratio can be related to the cross-link density using the Flory–Rehner equation where the swelling ratio depends on the molecular weight of the chains between effective cross-links.^38^ The cross-link density thus decreases, and the number of unlinked P/B species increases, in the order **N1**, **N2**, **N3** and **N4**.

**Figure 2:**
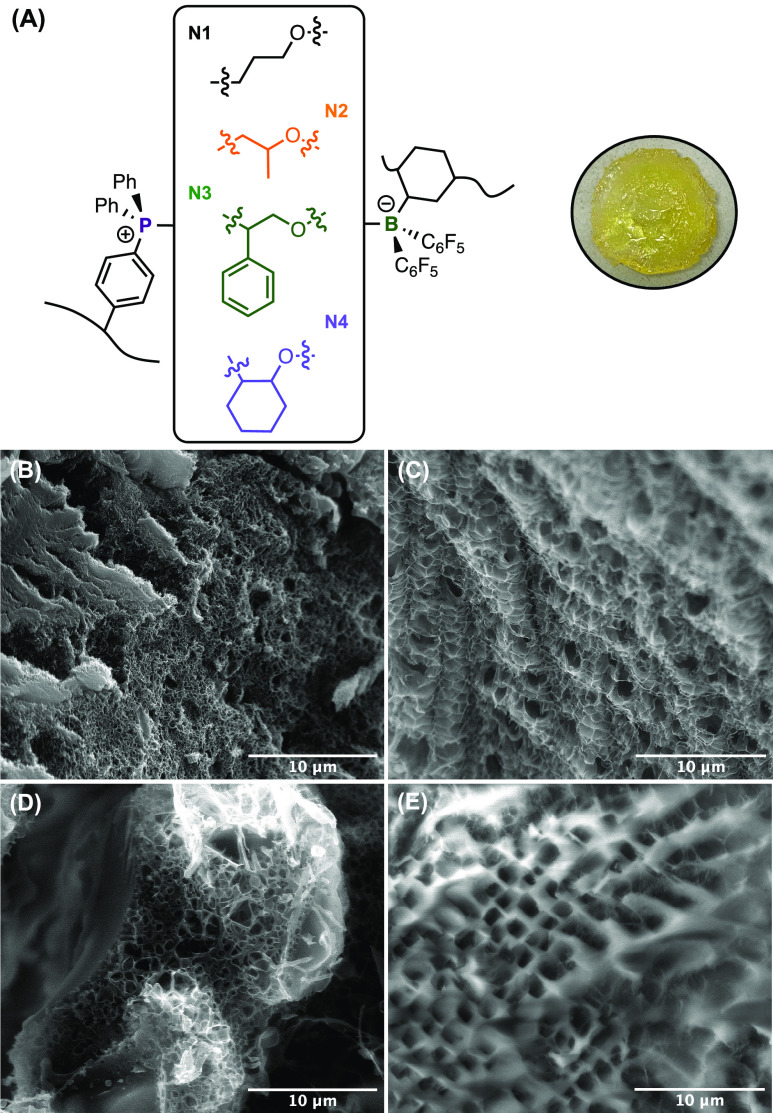
Illustrative representation of cross-linking chemical structures (A, left) and an example gel picture of N1 (A, right) and the SEM images to show the microstructure of the freeze-dried gels of **N1** (B), **N2** (C), **N3** (D), and **N4** (E).

Computational studies of small molecules showed the importance of both proximity and geometry of the LA and LB in ring-opening epoxides.^18^ The trend in cross-link density can be similarly explained: As LA coordination constitutes the initial step in ring opening,^14^ basicity and inductive effects play a dominant role at the beginning of gelation where chains are unencumbered. Increased basicity (**L1** < **L2** < **L3** < **L4**) results in stronger cyclic ether–LA coordination and induces rapid cross-linking, limiting polymer chain rearrangement and generating a nonhomogeneous microstructure. A lower energy barrier to ring opening has a similar impact as this also accelerates cross-linking over chain rearrangement. As the phenyl group on **L3** can resonance stabilize the positive charge generated by LA coordination and similarly **L4** through hyperconjugation,^39^ they promote the fastest cross-linking and lowest density. As gelation progresses and chains become more restricted, substrate steric influences predominate. Bulkier side chains (**L2** vs **L1**) hinder further cross-linking and thus preclude higher cross-linking densities. While in our first-generation systems, this rapid cross-linking was then followed by rearrangement to thermodynamically optimized gels,^4,5^ cyclic ether ring opening forms kinetically trapped gels (*vide infra*).

The microstructures of the poly(FLP) networks were characterized using scanning electron microscopy (SEM). The cross-sectional morphology of all samples showed noticeably clear continuous 3D polymer mesh and porous structure (Figures 2B–E and S35–S38). **N1** revealed a very dense microstructure, with pore diameters from 100 to 400 nm and an irregular continuous polymer mesh. These relatively small pore sizes support the smaller solvent uptake capacity of this polymer network. **N2** showed a distinctive internal pattern compared to that of **N1**, with closed-cell pores and thin polymer walls resulting in pores roughly 5 times larger in diameter. **N3** and **N4** showed a much wider range of pore diameters, from several hundred nanometers to micrometers. The random distribution and irregular structures confirm that steric hindrance and basicity influence cross-linking at a structural level.

The mechanical properties of these poly(FLP) gels were characterized using rheology. With all the polymeric networks, the storage (*G*′) moduli dominated over the loss (*G*′′) moduli over all the tested frequencies under small-amplitude oscillatory shear (Figure 3A). This suggests that most of the deformation energies were stored elastically within the networks, suggesting that a covalently cross-linked polymer network is formed. This was attributed to the release of ring strain preventing the thermodynamically unfavorable reverse reaction, leading to a behavior that is dramatically different from all previous poly(FLP) gels that act as reversible supramolecular assemblies with a strong frequency dependency.^8^ This enabled the preparation of poly(FLP)s with tunable mechanical properties. Although *G*′ is not completely independent of frequency, as would be expected from a perfectly covalently cross-linked polymer network, its dependency is plateau-like suggesting an imperfectly cross-linked network with dangling polymer chains.^40^ The power law dependency of *G*′ to ω was determined (Figure S41) and increased from **N1** to **N4**, with higher values correlating with more imperfections in the network structures, in agreement with the SEM results. Moduli values can be related to the stiffness of the polymeric networks and ranged from 10^5^ to 10^2^ Pa with **N1** being the stiffest, followed by **N2**, **N3**, and **N4**, respectively. The affine network model, where the modulus of a polymer network increases with its cross-link density,^40^ suggests that cross-linking density decreases from **N1** to **N4**, in agreement with the swelling data.

**Figure 3:**
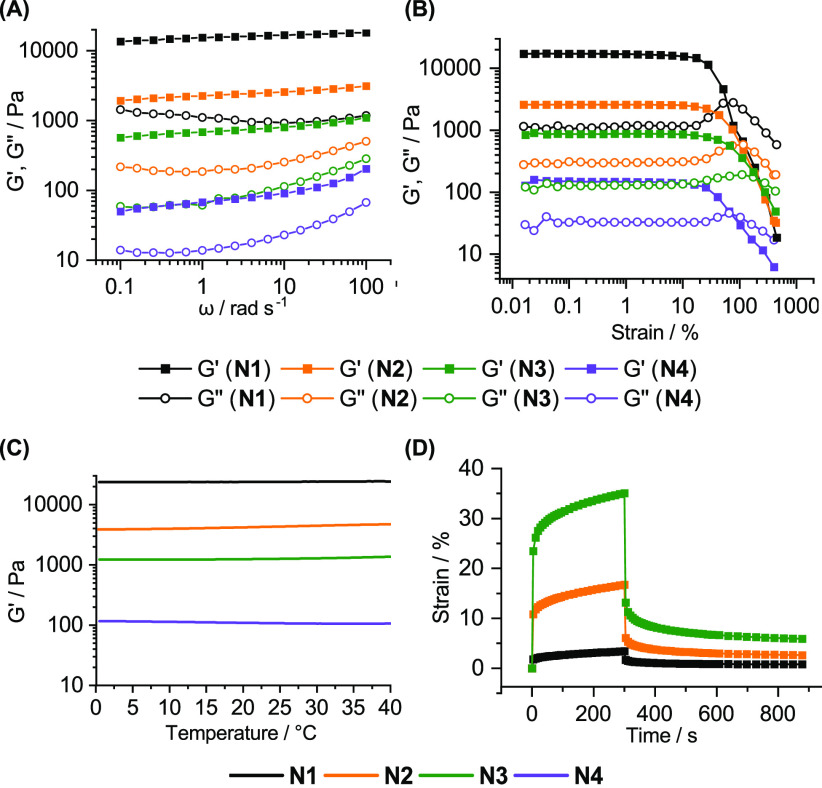
Rheological characterization of the prepared poly(FLP) networks. Storage and loss moduli values were tested against various frequencies (A), amplitudes (B) and temperatures (C), and the creep-recovery behaviors (D) were recorded under constant applied stress.

Amplitude sweep tests (Figures 3B and S43–S44) further support these mechanically tunable networks, as **N1** has the highest yield stress (3200 Pa) and the lowest yield strain (28%) (Table S5), followed by **N2** (810 Pa, 52%), **N3** (410 Pa, 73%), and **N4** (62 Pa, 98%). Only marginal changes were observed in the moduli values when temperature was varied from 0 to 40 °C at a constant frequency and under a small strain (Figure 3C), demonstrating the stability of the polymeric gels over the tested range. Creep-recovery behaviors of **N1**, **N2**, and **N3** confirmed the strong viscoelastic behaviors of these poly(FLP) networks (Figures 3D and S46). Creep strain values of 3, 17, and 36% were observed for **N1**, **N2**, and **N3**, respectively. An increased number of junctions precludes macromolecular movement, preventing strain from increasing.

While covalently linked, the gels remain responsive. Triggered degradation of **N1** and **N2**, our most robust polymer networks, is achieved through addition of either a stronger Lewis acid (BCl_3_) or Lewis base (pyridine). LA-induced degradation is particularly rapid, with gels degrading within minutes back to polymeric species (Figures S49−S51, Movie S3) and pyridine degradation occurring over days.

While this first disclosure of the system precluded full development of an application of these materials, interesting avenues for future investigations are found in the observation of macroscopic changes in the gels. Upon the addition of excess epoxides, unique color patterns were observed, suggesting future opportunities as chemical sensors (Figure S59A,B), while for oxetane-based networks, lowering the cross-linking density accesses **L1**-linked polymer networks that serve as triggerable adhesives (Figure S59C).

In summary, we report the first cyclic-ether-triggered P,B-based polymer networks. This new chemistry exploited a novel, easily prepared, polymeric Lewis acid using the versatility of hydroboration chemistry. The ring-opening reactions of cyclic ethers gave poly(FLP) gels which behaved as covalently cross-linked networks, contrasting with previous systems, and show that both reactivity and function can be tuned. Our current efforts are exploring the reactivity of these captured epoxides and an increased diversity of small-molecule triggers.

## Supporting Information Available

Detailed experimental synthetic procedures, spectral data for all reactions, molecules and polymers, network preparation and characterization, and additional data (PDF)
Videos showcasing the preparation of the triggered polymer network from poly(FLP)s (Movies S1 and S2, MP4, MP4) and the subsequent degradation with BCl_3_ (Movie S3, MP4)
