# Supplementary material for: Cyclic Ether Triggers for Polymeric Frustrated Lewis Pair Gels
Source: J Am Chem Soc. 2021 Aug 13;143(33):12980–4. doi: 10.1021/jacs.1c06408 (PMC8397318; doi:10.1021/jacs.1c06408)
Supplement: Supplementary file 1 — ja1c06408_si_001.pdf [file ja1c06408_si_001.pdf]

Supporting Information for

Cyclic Ether Triggers for Polymeric Frustrated Lewis Pair Gels

Utku Yolsal,† Thomas A. R. Horton,† Meng Wang,† and Michael P. Shaver *,†,‡

† Department of Materials, School of Natural Sciences, University of Manchester, Manchester, U.K.

‡ Sustainable Materials Innovation Hub, Henry Royce Institute, University of Manchester, Manchester, U.K.

*michael.shaver@manchester.ac.uk

Table of Contents

General Considerations 2

Materials 2

Instrumentation 3

Preparations of Polymeric LAs and LBs 3

SYN1. Synthesis of poly(styrene-*co*-1,3-cyclohexadiene) 3

SYN2. Synthesis of poly(styrene-*co*-cyclohexyl-bis(pentafluorophenyl)borane) 4

SYN3. Synthesis of poly(styrene-*co*-4-styryl-diphenylphosphine) 6

Guttmann-Beckett Tests 8

SYN4. Lewis acidity of $CyB(C_6F_5)_2$ 8

SYN5. Lewis acidity of poly(styrene-*co*-cyclohexyl-bis(pentafluorophenyl)borane) 9

Synthesis of Small Molecules 10

SYN6. Synthesis of cyclohexyl-bis(pentafluorophenyl)borane 10

SYN7. Synthesis of SM1 11

SYN8. Synthesis of SM2 15

SYN9. Synthesis of SM3 18
SYN10. Synthesis of SM4 22
NMR Characterization of Product Connectivity 27
Network Formation and Characterisation 30
General procedure for the preparation of polymeric FLP networks 30
Gel fractions and swelling ratio of the polymeric networks 31
ATR-FTIR spectra of the xerogels 32
Polymer network preparation for SEM 33
SEM Images 33
Polymer network preparation for rheological characterisation 36
Rheological characterisation 37
Degradation of the Polymer Networks N1 and N2 44
Exposure to Boron Trichloride 44
Exposure to Pyridine 47
Poly(FLP)s as Functional Materials 52
References 52

General Considerations

All oxygen- and moisture-sensitive reactions were performed under an inert atmosphere using standard Schlenk techniques on a dual-manifold Schlenk line equipped with an in-line gas drying column containing a copper catalyst or in an argon-filled glovebox, unless otherwise stated. All glassware for air/moisture-sensitive reactions were pre-dried in an oven (200 °C) overnight.

Materials

Anhydrous toluene (Tol), dichloromethane (DCM), *n*-hexane (Hex) and tetrahydrofuran (THF) were obtained using an mBraun Solvent Purification System containing alumina and copper catalysts. Cyclohexene, 1,2-propylene oxide (PO), oxetane (1,3-propylene oxide, OX), styrene oxide (SO), cyclohexene oxide (CHO), styrene, cyclohexane, 1,3-cyclohexadiene (1,3-CHD), benzene, chloroform-*d*, DCM-d_2 and tol-d_8 were purchased from Sigma-Aldrich

and distilled over CaH_2 (Acros Organics) under inert atmosphere. All solvents and the distilled reagents were degassed by freeze-pump-thaw for three cycles before use. Anhydrous pyridine, triphenylphosphine, triethylphosphine oxide and 1.4 M *sec*-butyllithium (*sec*-BuLi) in cyclohexane were purchased from Sigma-Aldrich and methanol (reagent grade) was purchased from Fisher Scientific and they were used as received. Bis(pentafluorophenyl)borane and 4-styryl-diphenylphosphine were synthesised using the reported literature procedures.[1, 2]

Instrumentation

^1H, ^{11}B, ^{13}C, ^{19}F and ^{31}P nuclear magnetic resonance (NMR) data were obtained using 400 MHz Bruker AVIII (BBFO 5 mm probe), 400 MHz Bruker AVIII HD (BBO 5mm probe) and 500 MHz Bruker AVIII HD (BBO 5 mm probe) spectrometers. Young's tap NMR tubes were used for all the air-/moisture-sensitive samples. Exact masses of the small molecule mimics were determined using a Thermo Orbitrap QExactive mass spectrometer in electrospray ionisation (ESI) mode by using DCM as solvent. Molecular weights of the polymers were determined using an Agilent 1260 Infinity II Multi-Detector Gel Permeation Chromatography (GPC)/Size Exclusion Chromatography System through PLgel 5 µm columns packed with PSDVB beads. The GPC was run with THF at a flow rate of 1.00 ml min^{-1} at 35 °C. Molecular weights were obtained using triple detection based on a calibration prepared with narrow dispersity polystyrene standards. Fourier transform infrared (FTIR) spectra were obtained using a Nicolet 5700 ATR-FTIR instrument with a Smart Orbit diamond single bounce crystal. The data reported are the average of 32 scans and were acquired using a resolution of 4 cm^{-1} at room temperature. Rheological characterisations of the prepared gels were performed using a TA Discovery Hybrid Rheometer (DHR) 2 with parallel plate geometry. Temperature of the experiments were controlled using the TA Peltier Plate smooth geometry. Top geometry was a 20 mm diameter cross-hatched tool for all the measurements. Scanning electron microscopy (SEM) images were take using a FEI Quanta 250 FEG-SEM with Oxford Instrument EDS and GATAN 3view system. All equation fittings were performed using Origin Professional 2020b and the SEM images were analysed using ImageJ.

Preparations of Polymeric LAs and LBs

SYN1. Synthesis of poly(styrene-*co*-1,3-cyclohexadiene)

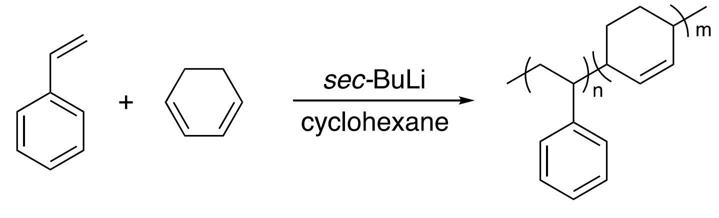

This synthesis was modified from a previous literature report.[3] Styrene (4.00 g, 38.4 mmol) and 1,3-cyclohexadiene (0. 34 g, 4.3 mmol, 1,3-CHD) were dissolved in cyclohexane (25 ml). The solution was stirred before the addition of *sec*-BuLi (1.4M in cyclohexane, 0.17 ml, 0.24 mmol) to initiate the polymerization which changed the colour of the solution to orange. The reaction mixture was allowed to stir for 3 hours, before being quenched with a small amount of methanol. The colourless mixture was then precipitated into methanol twice and the copolymer was recovered by filtration. The product was dried under high vacuum overnight, yielding a fine white powder (3.92 g, 90%).

$\mathbf{M_n}$ / g mol^{-1} 18000 (theoretical), 21400 (experimental), Đ = 1.1.

^{1}H NMR (400 MHz, 294 K, $CDCl_3$) δ / ppm 7.36-6.90 (br, *o*-C_6H_5, *p*-C_6H_5), 6.83-6.27 (br, *m*-C_6H_5), 5.93-5.23 (br, HC=CH), 2.52-1.09 (br, CH (alkyl), CH_2).

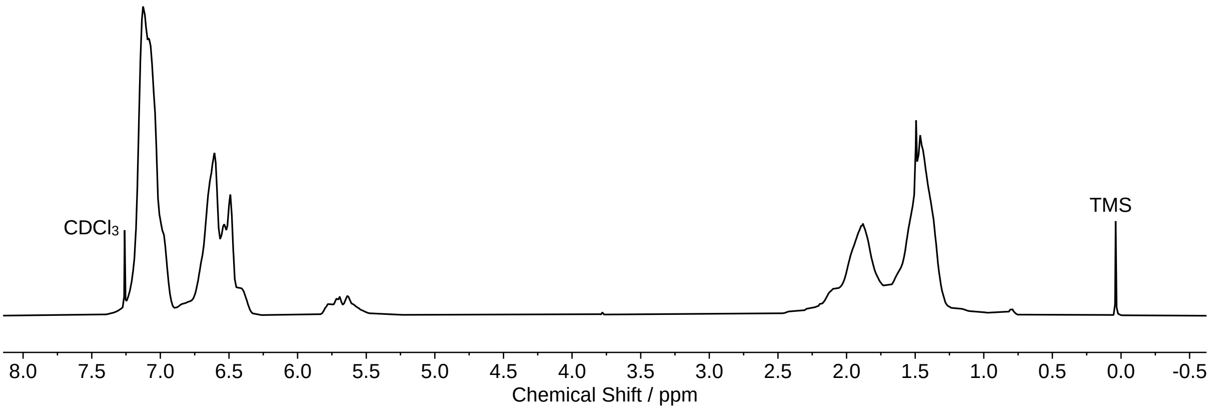

Figure S1: ^{1}H NMR spectrum of poly(styrene-*co*-1,3-CHD).

SYN2. Synthesis of poly(styrene-*co*-cyclohexyl-bis(pentafluorophenyl)borane)

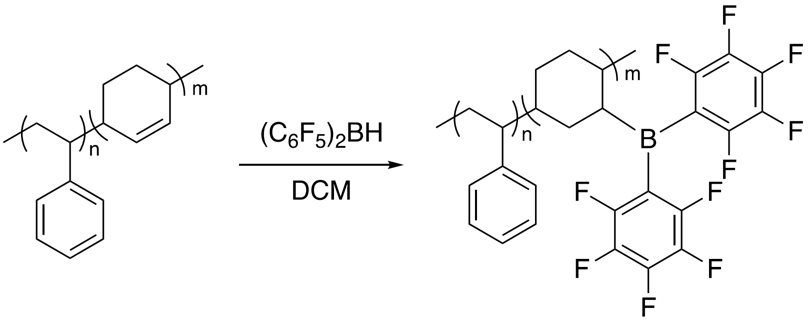

Poly(styrene-*co*-1,3-CHD) (1.00 g, 55.6 μmol) and bis(pentafluorophenyl)borane (0.48 g, 1.11 mmol) were mixed in DCM (5 ml) to form a cloudy solution. Upon stirring the mixture overnight at ambient temperature, the solution became colourless. DCM was removed *in vacuo* and the copolymer was dissolved in toluene (4 ml) prior to precipitating into hexanes (20 ml). This precipitation was repeated three times prior to filtration. The product was dried under vacuum overnight to yield a white powder (0.96 g, 65%).

M_n / g mol^{-1} 25000 (theoretical), 32000 (experimental), Đ = 1.2.

^{1}H NMR (400 MHz, 298 K, $CDCl_3$) / ppm 7.29-6.80 (br, *o*-C_6H_5, *p*-C_6H_5), 6.75-6.21 (br, *m*-C_6H_5), 2.14-1.06 (br, CH (alkyl), CH_2).

$^{11}B\{^{1}H\}$ NMR (128 MHz, 298 K, $CDCl_3$) δ / ppm not observed (n.o.)

^{19}F NMR (376 MHz, 298 K, $CDCl_3$) δ / ppm -127.5- -113.8 (br, *o*-C_6F_5), -147.7- -152.6 (br, *p*-C_6F_5), -159.8- -163.9 (br, *m*-C_6F_5).

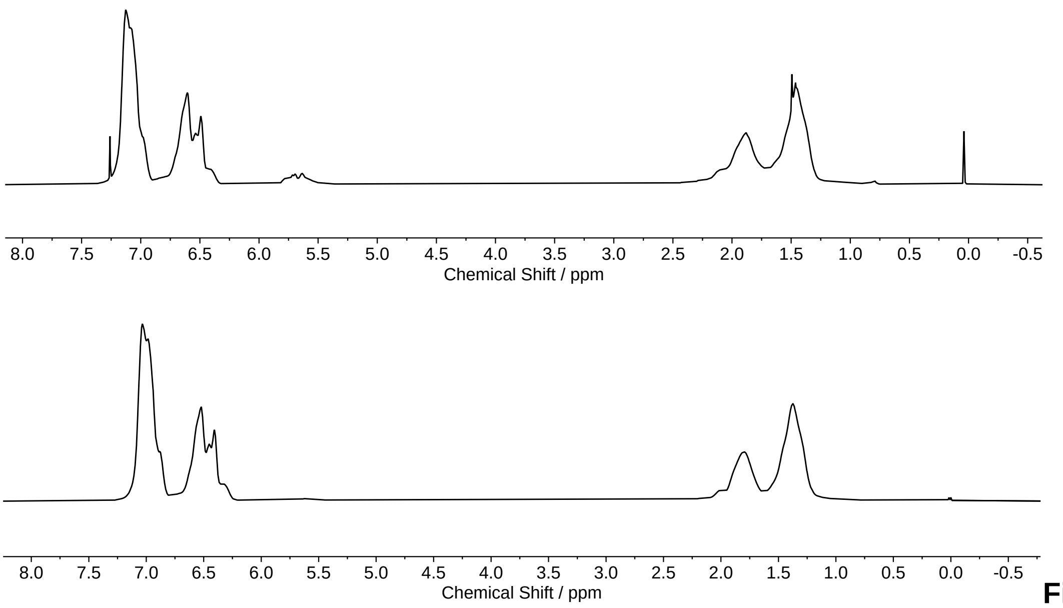

Figure S2: ^{1}H NMR spectra of poly(Sty-co-1,3-CHD) (top) and the post-polymerization functionalized copolymer (bottom).

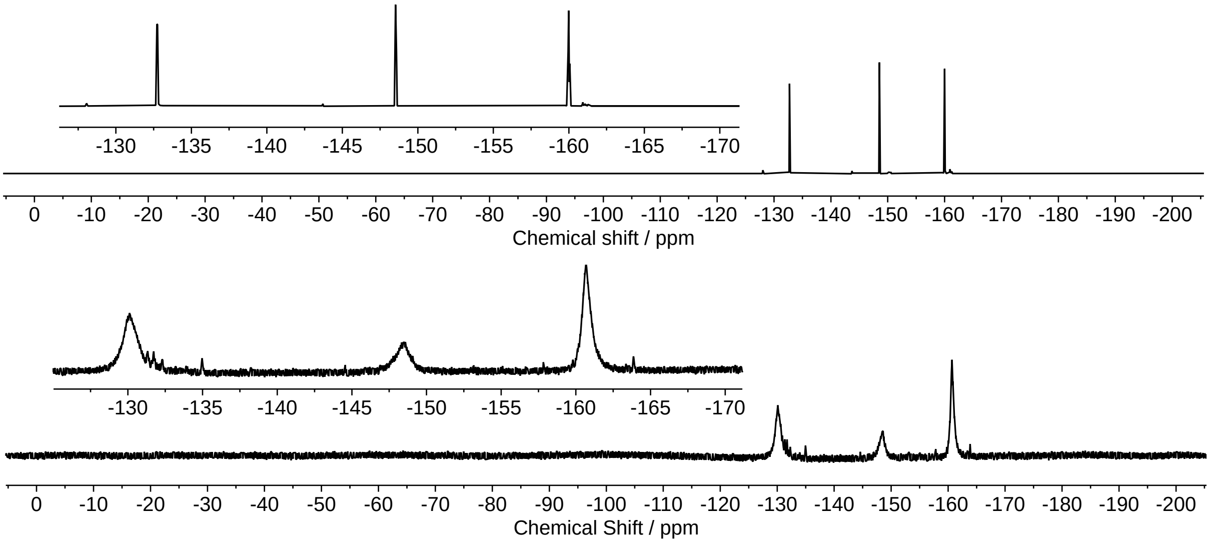

Figure S3: ^{19}F NMR spectra of bis(pentafluorophenyl)borane (top) and the post-polymerization functionalized copolymer (bottom).

SYN3. Synthesis of poly(styrene-*co*-4-styryl-diphenylphosphine)

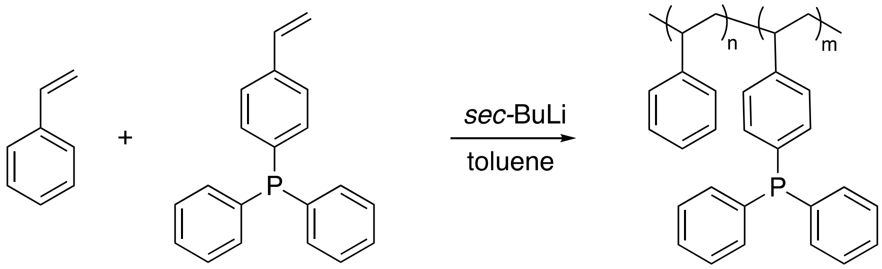

Pre-weighed amounts of styrene (2.60 g, 24.9 mmol) and 4-styryl-diphenylphosphine (0.72 g, 2.5 mmol) were dissolved in toluene (9 ml). The solution was stirred before addition of *sec*-BuLi (1.4M in cyclohexane, 0.10 ml, 0.14 mmol) to initiate the polymerization which changed the colour of the solution to red. The reaction mixture was allowed to stir for 5 hours, before being quenched with a small amount of methanol. The colourless mixture was then precipitated twice into methanol, before filtration and drying under vacuum obtained the desired product as a fine white powder (2.8 g, 84%).

M_n / g mol^{-1} 23200 (theoretical), 22300 (experimental), Đ = 1.1.

^{1}H NMR (400 MHz, 295 K, DCM-*d_2*) / ppm 7.42-6.87, 6.81-6.25 (br, C_6H_5), 2.20-1.11 (br, CH (alkyl), CH_2).

^{31}P NMR (162 MHz, 295 K, DCM-*d_2*) δ / ppm -6.5 (br, $\omega_{1/2}$ = ~52 Hz).

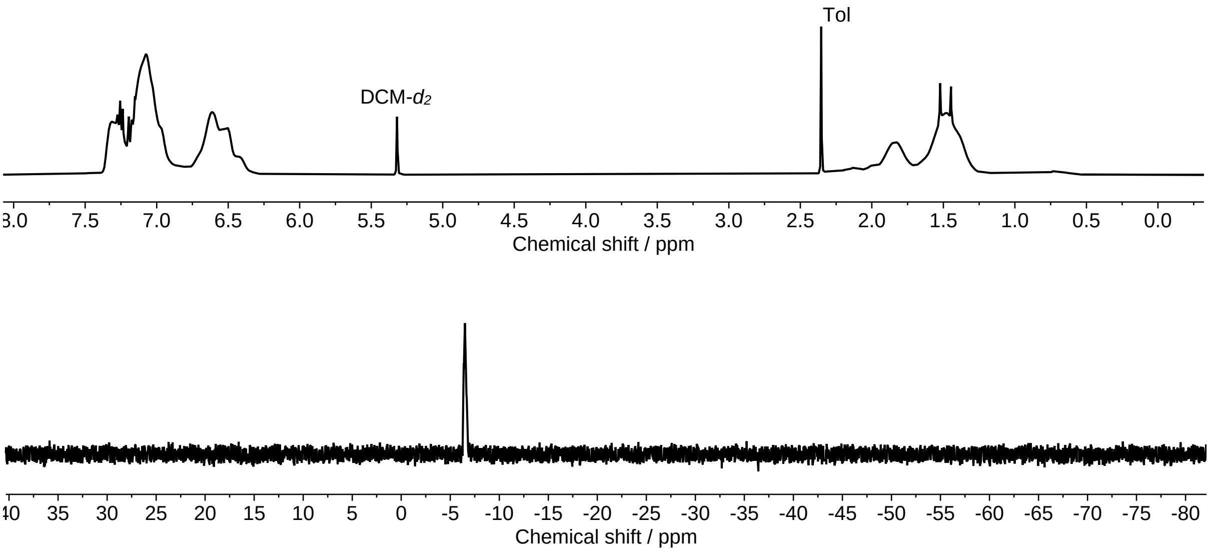

Figure S4: ^{1}H (top) and ^{31}P (bottom) NMR spectra of poly(Sty-*co*-PPh_3).

Table S1: Parameters of the prepared copolymers. Experimental M_n and Đ values were obtained by using a triple detection THF GPC.

Polymer	Mol fraction (%)		$M_{n(theo)}$ (g/mol)	$M_{n(exp)}$ (g/mol)	Đ
	Sty	Comonomer			
**P1**	89	11	18000	21400	1.1
**P2**	89	11	25000	32000	1.2
**P3**	90	10	23200	22300	1.1

Guttmann-Beckett Tests

SYN4. Lewis acidity of $CyB(C_6F_5)_2$

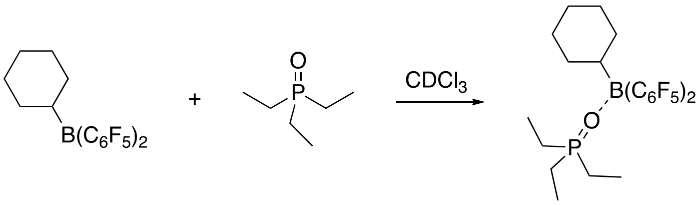

$CyB(C_6F_5)_2$ (32 mg, 75 μmol) and triethylphosphine oxide (10 mg, 75 μmol) were dissolved in deuterated chloroform (0.7 ml) inside a Young's NMR tube at RT.

^{1}H NMR (400 MHz, 298 K, $CDCl_3$) δ / ppm 2.04-1.53, 1.39-0.96, 0.74-0.55 (m, 11H, Cy), 1.39-0.96, 0.74-0.55 (m, 11H, Cy), 1.83 (dq, 6H, CH_2-P, $J_{P,H}$ 12 Hz, $J_{H,H}$ 7.7 Hz), 1.15 (dt, 9H, CH_3, $J_{P,H}$ 18 Hz, $J_{H,H}$ 7.7 Hz).

^{11}B{^{1}H} NMR (128 MHz, 298 K, $CDCl_3$) δ / ppm 4.24 (br, $\omega_{1/2}$ = ~300 Hz).

^{19}F NMR (376 MHz, 298 K, $CDCl_3$) δ / ppm -132.1 (m, 4F, *o*-C_6F_5), -159.8 (t, 2F, *p*-C_6F_5, $J_{F,F}$ = 20 Hz), -164.4 (m, 4F, *m*-C_6F_5).

^{31}P NMR (162 MHz, 298 K, $CDCl_3$) δ / ppm 72.3 (br, $\omega_{1/2}$ = ~50 Hz).

Lewis acceptor number (AN) of $CyB(C_6F_5)_2$ = 2.21 × (δ $^{31}P_{LA\cdot Et3PO}$ - 41.0) = 69.2 ($CDCl_3$).

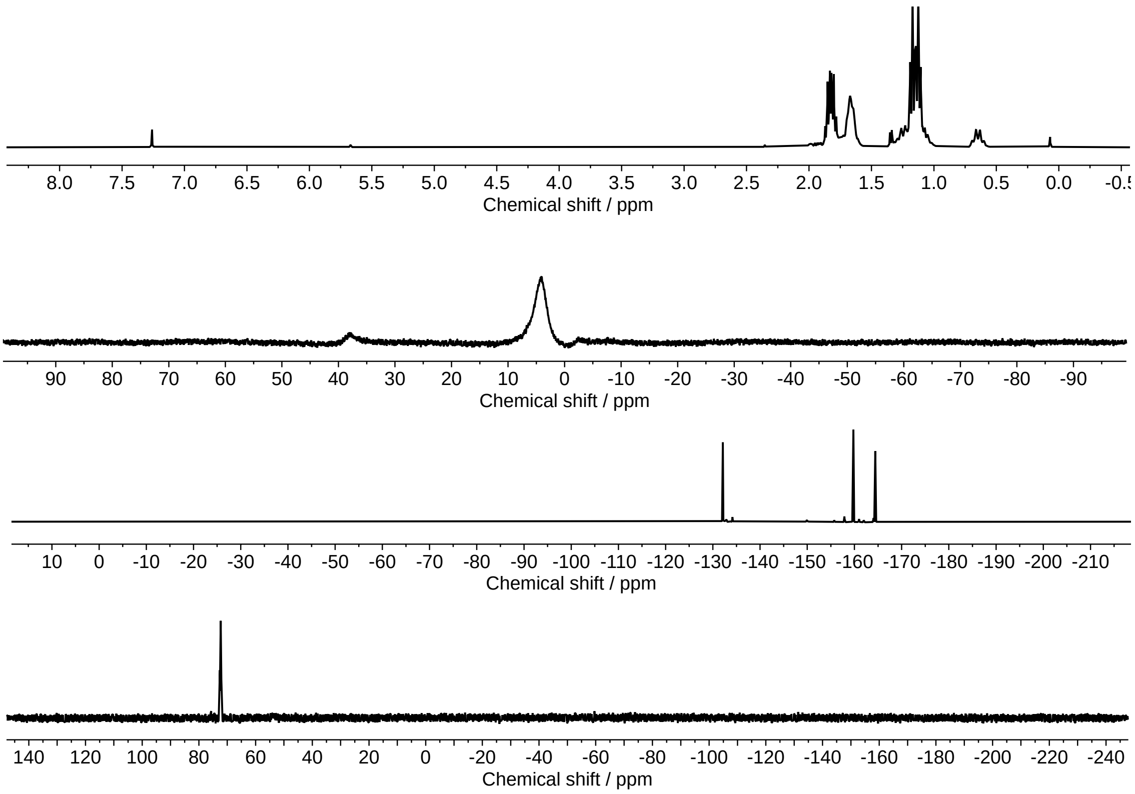

Figure S5: ^{1}H, ^{11}B, ^{19}F and ^{31}P (top to bottom) NMR spectra of CyB(C_6F_5)$_2$.Et$_3$PO adduct.

SYN5. Lewis acidity of poly(styrene-*co*-cyclohexyl-bis(pentafluorophenyl)borane)

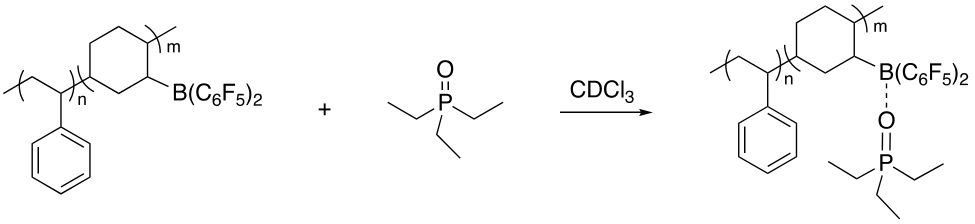

Poly(Sty-*co*-CyB(C_6F_5)$_2$) (50 mg, 2.0 μmol) and triethylphosphine oxide (5.4 mg, 20 μmol) were dissolved in toluene (1 ml) and stirred at RT overnight. The resulting solution was precipitated into hexane (5 ml) and recovered by filtration. Obtained white powder was dried overnight under reduce vacuum to form the copolymer adduct (22 mg, 40%).

^{1}H NMR (400 MHz, 298 K, Tol-*d$_8$*) δ / ppm 7.41-6.88 (br, *o*-C_6H_5, *p*-C_6H_5), 6.88-6.34 (br, *m*-C_6H_5), 2.38-1.92, 1.90-1.39, 1.39-1.08, 0.87-0.56, 0.56-0.24 (br, CH (alkyl), CH_2, CH_3).

^{11}B{^{1}H} NMR (128 MHz, 298 K, Tol-*d$_8$*) δ / ppm n.o.

^{19}F NMR (376 MHz, 298 K, Tol-*d$_8$*) δ / ppm -127.9- -134 (br, 4F, *o*-C_6F_5), -157.5- -161.5 (br, 2F, *p*-C_6F_5), -162.3- -167.2 (br, 4F, *m*-C_6F_5).

^{31}P NMR (162 MHz, 298 K, Tol-*d$_8$*) δ / ppm 71.9 (br, $\omega_{1/2}$ = ~230 Hz).

AN of poly(Sty-*co*-CyB(C_6F_5)$_2$) = 2.21 × (δ $^{31}P_{LA \cdot Et3PO}$ - 41.0) = 68.3 (Tol-*d*$_8$).

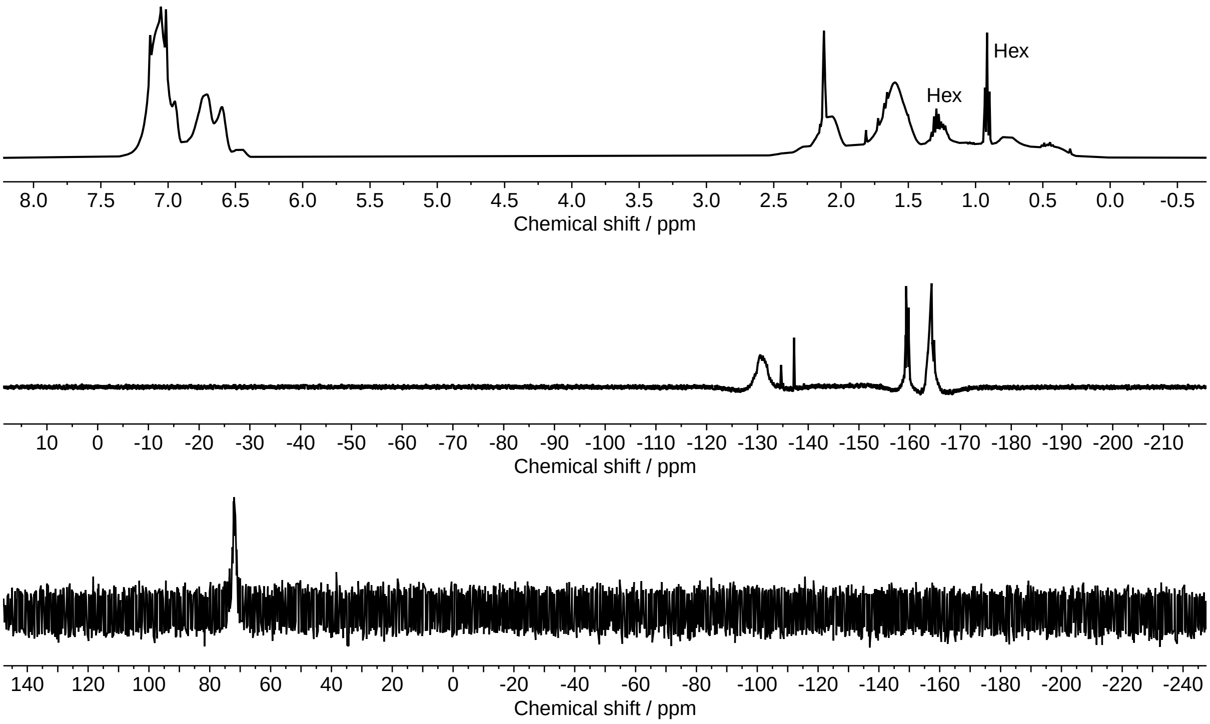

Figure S6: ^{1}H, ^{19}F and ^{31}P (top to bottom) NMR spectra of poly(Sty-*co*-CyB(C_6F_5)$_2$).Et_3PO adduct.

Synthesis of Small Molecules

SYN6. Synthesis of cyclohexyl-bis(pentafluorophenyl)borane

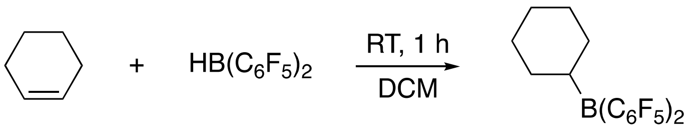

The synthesis was modified from a previous literature report.[4] To a stirred suspension of bis(pentafluorophenyl)borane (1.00 g, 2.89 mmol) in DCM (5 ml), cyclohexene (0.30 g, 3.65 mmol) was added dropwise. The mixture was stirred for 1 hour and the solvent was removed from the transparent solution *in vacuo,* yielding cyclohexyl-bis(pentafluorophenyl)borane (CyB(C_6F_5)$_2$) as a white powder (1.18 g, 95%). The product can also be re-crystallized from hexane. Full characterisation can be found in the cited literature.

^{1}**H NMR** (400 MHz, 298 K, $CDCl_3$) δ / ppm 2.23-2.12 (m, 1H, B-CH), 1.89-1.70, 1.46-1.19 (m, 10H, Cy-H).

^{19}F NMR (376 MHz, 298 K, $CDCl_3$) δ / ppm -130.1 (m, 4F, *o*-C_6F_5), -149.1 (tt, 2F, *p*-C_6F_5), -160.9 (m, 4F, *m*-C_6F_5).

^{11}B{^{1}H} NMR (128 MHz, 298 K, $CDCl_3$) δ / ppm 75.3 (s, br, $\omega_{1/2}$ = ~ 570 Hz).

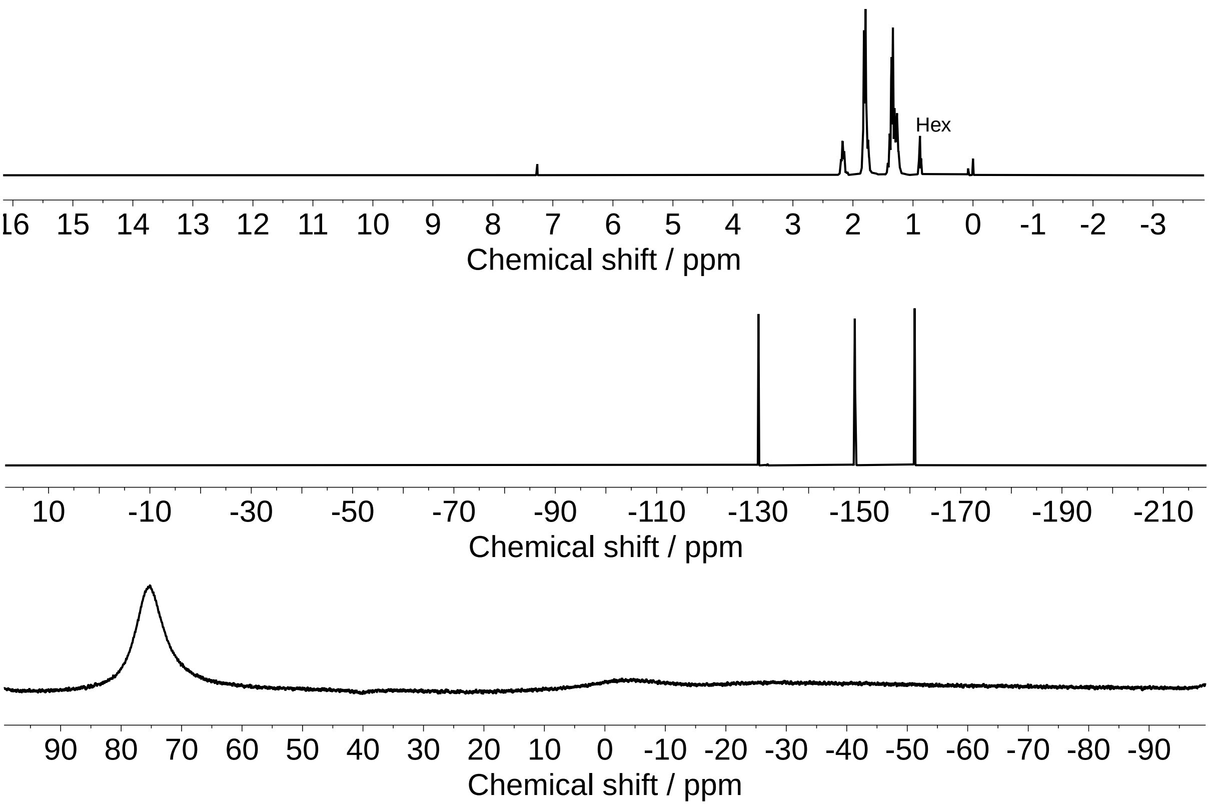

Figure S7: ^{1}H, ^{19}F and ^{11}B (top to bottom) NMR spectra of $CyB(C_6F_5)_2$.

SYN7. Synthesis of SM1

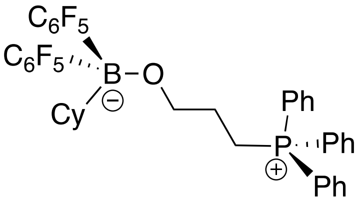

Under an inert atmosphere, a solution of oxetane (20 mg, 0.35 mmol) in toluene (2 ml) was added dropwise to a stirred suspension of PPh_3 (92 mg, 0.35 mmol) and $CyB(C_6F_5)_2$ (0.15 g, 0.35 mmol) in toluene (3 ml) at 0 °C. The resulting solution was stirred at this temperature for 5 mins, before being allowed to warm to room temperature for a further 30 mins stirring. Upon completion, anhydrous hexane was added, resulting in the precipitation of a white solid. The solution was recrystallized in the glovebox freezer (-35 °C) overnight to obtain a white crystalline product in 77% yield (0.20 g).

^{1}H NMR (400 MHz, 294 K, DCM-*d$_2$*) δ / ppm 7.85-7.77 (m, 3H, *p*-C_6H_5), 7.75-7.62 (m, 12H, *m*-C_6H_5, *o*-C_6H_5), 3.32-3.17 (m, 4H, O-CH_2, P-CH_2), 1.84-1.74 (d, 2H, Cy, $J_{H,H}$ 13 Hz), 1.74-1.55 (m, 5H, C*H$_2$*CH_2P, Cy), 1.36-1.17 (m, 3H, Cy), 1.03 (qt, 1H, Cy, $J_{H,H}$ 13, 4 Hz), 0.57 (q, 1H, Cy, J 12 Hz).

^{13}C{^{1}H} NMR (126 MHz, 294 K, DCM-*d$_2$*) δ / ppm 148.4 (dm, *o*-C_6F_5, $J_{F,C}$ 236 Hz), 137.7 (dm, *p*-C_6F_5, $J_{F,C}$ 244 Hz), 136.7 (dm, *m*-C_6F_5, $J_{F,C}$ 247 Hz), 127.2 (br, *i*-C_6F_5), 135.4 (d, *p*-C_6H_5, $J_{P,C}$ 3.0 Hz), 133.8 (d, *m*-C_6H_5, $J_{P,C}$ 9.8 Hz), 130.8 (d, *o*-C_6H_5, $J_{P,C}$ 12 Hz), 119.2 9 (d, *i*-C_6H_5, $J_{P,C}$ 86 Hz), 60.9 (d, O-CH, $J_{P,C}$ 14 Hz), 34.0 (br, B-CH), 30.9, 30.0, 29.7, 28.6, 28.3 (s, Cy), 26.4 (d, C*H$_2$*CH_2P, $J_{P,C}$ 4.5 Hz), 18.8 (d, P-CH_2, $J_{P,C}$ 54 Hz).

^{11}B{^{1}H} NMR (128 MHz, 294 K, DCM-*d$_2$*) δ / ppm 1.77 (br, $\omega_{1/2}$ = ~100 Hz).

^{19}F NMR (376 MHz, 294 K, DCM-*d$_2$*) δ / ppm -131.5 (m, 4F, *o*-C_6F_5), -164.6 (t, 2F, *p*-C_6F_5, $J_{F,F}$ = 20 Hz), -167.1 (m, 4F, *m*-C_6F_5).

^{31}P{^{1}H} NMR (162 MHz, 294 K DCM-*d$_2$*) δ / ppm 23.9 (s).

HR-ESI-MS calculated for $C_{39}H_{32}BF_{10}OP$ $[M+Na]^+$ 771.2022, found 771.2009.

IR (ATR) cm^{-1} 3064 (w), 3031 (w), 2914 (w), 2934 (w), 1637 (w), 1508 (m), 1448 (s), 1437 (s), 1388 (w), 1375 (w), 1359 (w), 1348 (w), 1273 (w), 1259 (w), 1205 (w), 1157 (w), 1115 (s), 1103 (s), 1074 (s), 1025 (w), 998 (w), 983 (w), 960 (s), 960 (m), 919 (w), 871 (w), 846 (w), 802 (w), 781 (w), 752 (m), 737 (s), 730 (s), 723 (s), 685 (s), 651 (w), 619 (w), 593 (w), 574 (w), 555 (w), 526 (s), 509 (s).

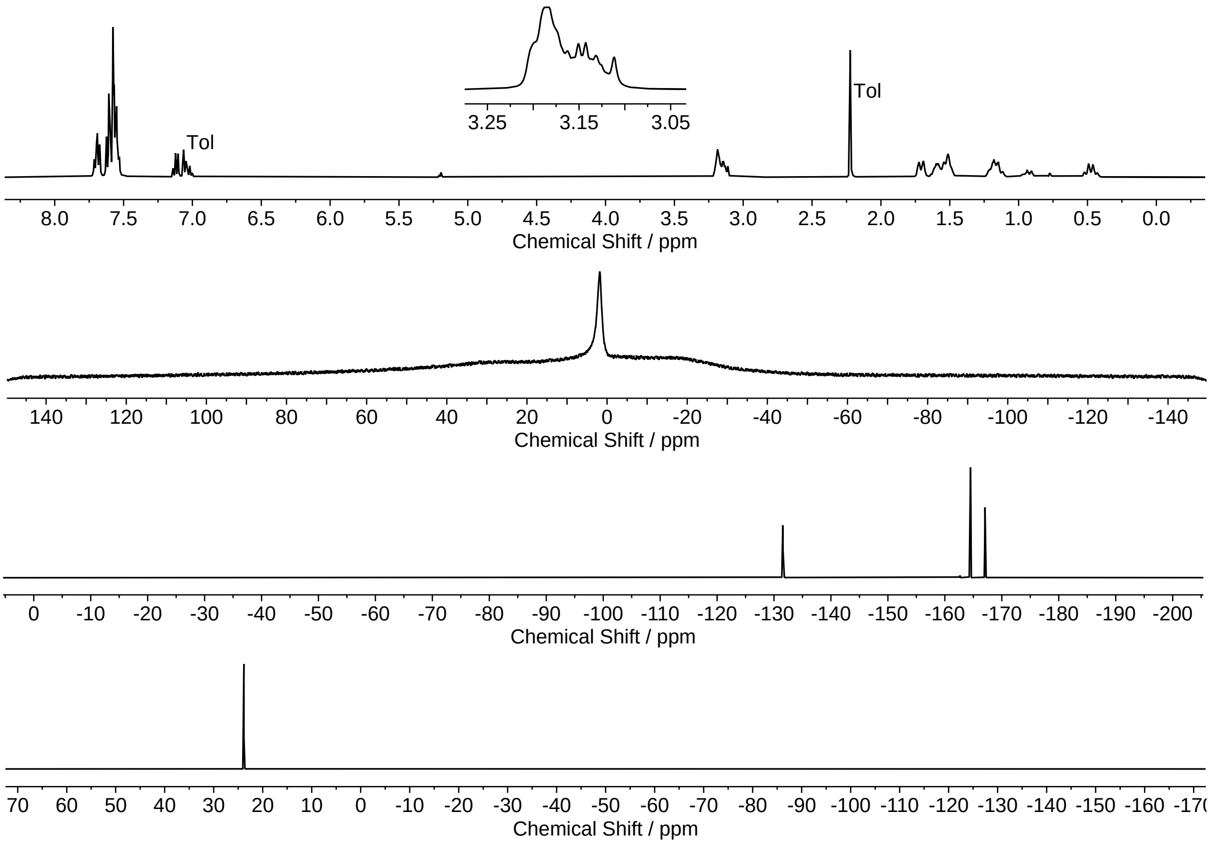

Figure S8: ^{1}H, ^{11}B, ^{19}F and ^{31}P (top to bottom) NMR spectra of **SM1**.

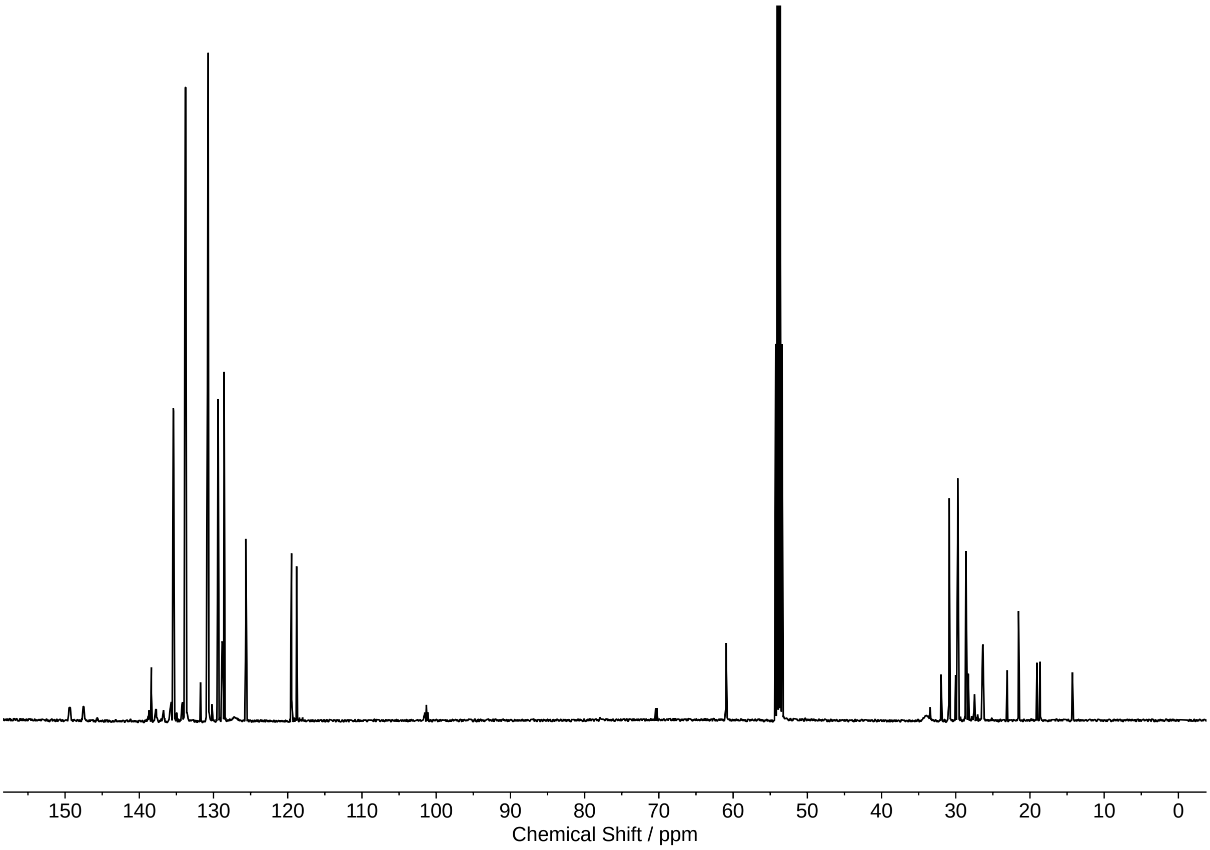

Figure S9: ^{13}C NMR spectrum of **SM1**.

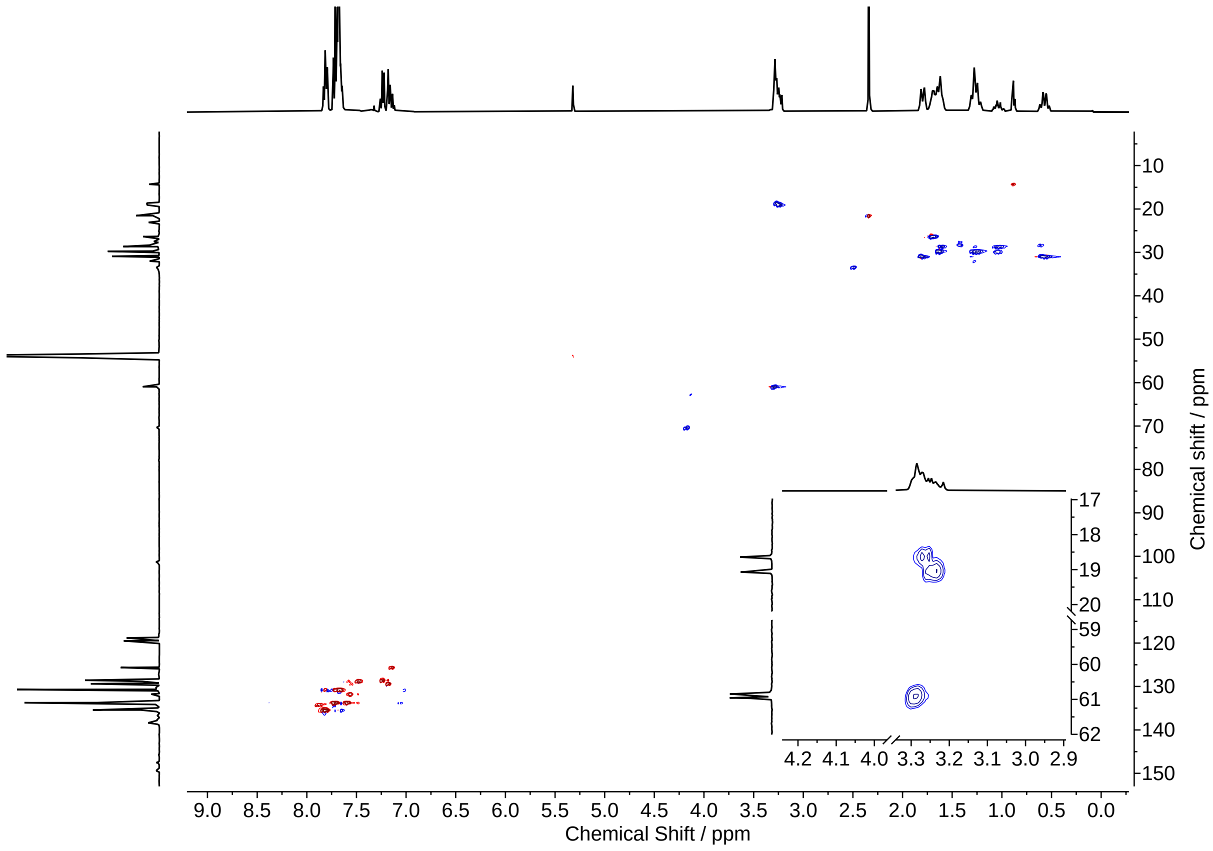

Figure S10: ^{1}H-^{13}C HSQC NMR spectrum of **SM1.**

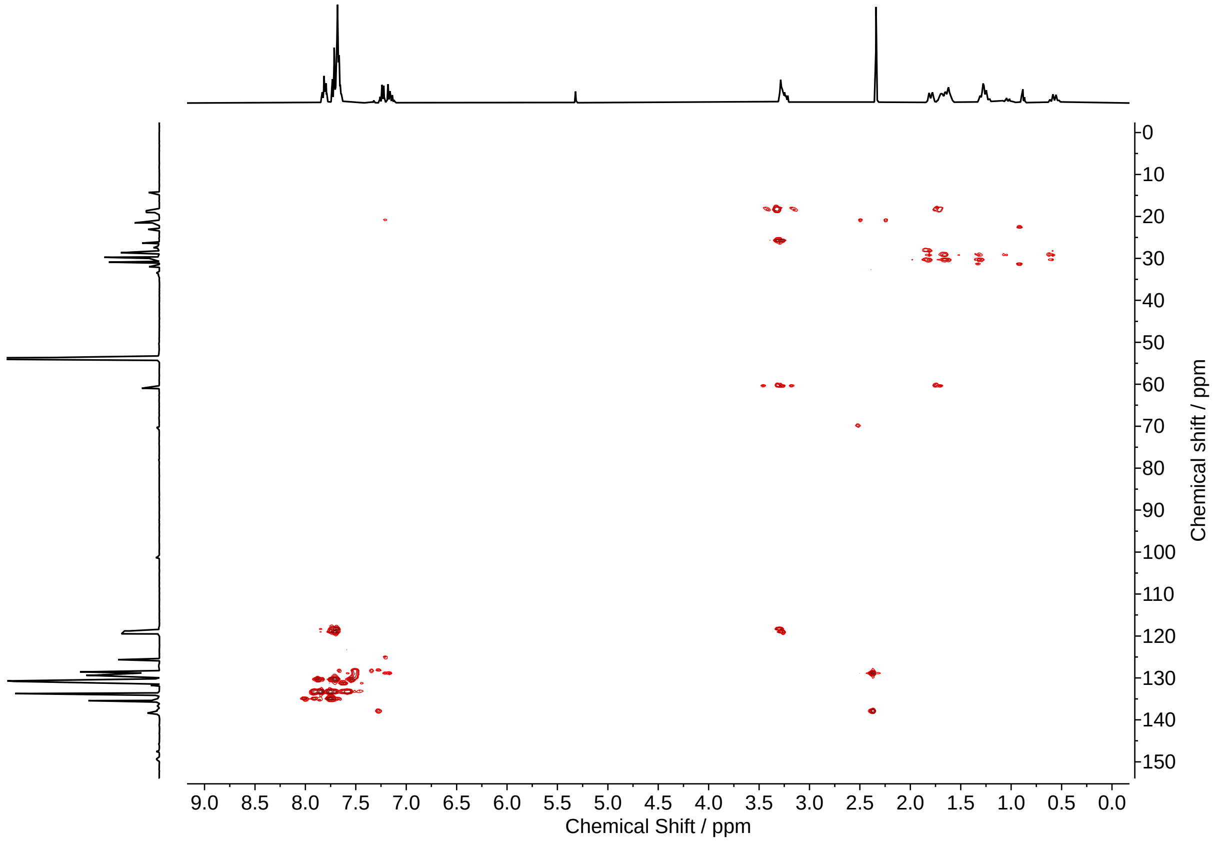

Figure S11: ^{1}H-^{13}C HMBC NMR spectrum of **SM1.**

SYN8. Synthesis of SM2

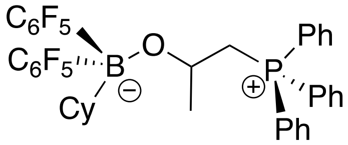

A solution of propylene oxide (20 mg, 0.35 mmol) in toluene (2 ml) was added dropwise to a stirred suspension of PPh_3 (92 mg, 0.35 mmol) and $CyB(C_6F_5)_2$ (0.15 g, 0.35 mmol) in toluene (3 ml) at 0 °C under an inert atmosphere. The resulting solution was stirred at this temperature for 15 mins and allowed to warm to room temperature for a further 30 mins stirring. Upon completion, anhydrous hexane was added via cannula, resulting in the precipitation of a white solid. Upon recrystallization in the glovebox freezer (-35 °C), colourless crystals were obtained in 61% yield (0.16 g), with slight formation of a yellow crystalline impurity.

^{1}H NMR (400 MHz, 294 K, DCM-*d₂*) δ / ppm 7.82-7.72 (m, 3H, *p*-C_6H_5), 7.68-7.51 (m, 12H, *m*-C_6H_5, *o*-C_6H_5), 3.83 (m, 1H, OC*H*(CH_3), $J_{H,H}$ = 12, 5.5 Hz), 3.36 (ddd, 1H, P-C*H*H, $J_{P,H}$ 15 Hz, $J_{H,H}$ 12, 5.5 Hz), 2.93 (ddd, 1H, P-CH*H*, $J_{P,H}$ 15 Hz, $J_{H,H}$ 12, 5.5 Hz), 1.73-1.64, 1.63-1.44, 1.22-0.87, 0.47-0.26 (m, 11H, Cy), 1.14 (d, 3H, CH_3, $J_{H,H}$ = 5.5 Hz).

^{13}C{^{1}H} NMR (126 MHz, 294 K, DCM-d_2) δ / ppm 148.0 (dm, *o*-C_6F_5, $J_{F,C}$ 235 Hz), 137.2 (dm, *p*-C_6F_5, $J_{F,C}$ 253 Hz), 136.0 (dm, *m*-C_6F_5, $J_{F,C}$ 255 Hz), *n.o.* (*i*-C_6F_5), 134.9 (d, *p*-C_6H_5, $J_{P,C}$ 3.1 Hz), 134.1 (d, *m*-C_6H_5, $J_{P,C}$ 10 Hz), 130.3 (d, *o*-C_6H_5, $J_{P,C}$ 12 Hz), 119.9 (d, *i*-C_6H_5, $J_{P,C}$ 86 Hz), 65.1 (d, O-CH, $J_{P,C}$ 4.6 Hz), 35.9 (d, P-CH_2, $J_{P,C}$ 47 Hz), 34.7 (br, B-CH), 31.0, 30.7, 29.8, 29.6, 28.5 (s, Cy), 25.0 (d, CH_3, $J_{P,C}$ 12 Hz).

^{11}B{^{1}H} NMR (128 MHz, 294 K, DCM-d_2) δ / ppm 1.38 (br, $\omega_{1/2}$ = ~140 Hz).

^{19}F NMR (376 MHz, 294 K, DCM-d_2) δ / ppm -131.6 (m, 4F, *o*-C_6F_5), -164.6 (t, 2F, *p*-C_6F_5, $J_{F,F}$ = 20 Hz), -167.3 (m, 4F, *m*-C_6F_5).

^{31}P{^{1}H} NMR (162 MHz, 294 K, DCM-d_2) δ / ppm 23.8 (s).

HR-ESI-MS calculated for $C_{39}H_{32}BF_{10}OPNa$ $[M+Na]^+$ 771.2022, found 771.2006.

FTIR (ATR) cm^{-1} 3056 (w), 3029 (w), 2912 (w), 2840 (w), 1639 (w), 1504 (m), 1437 (s), 1385 (w), 1340 (w), 1261 (m), 1203 (w), 1132 (w), 1112 (m), 1070 (s), 1027 (w), 998 (w), 960 (s),

939 (m), 904 (w), 893 (w), 852 (w), 817 (w), 800 (w), 779 (w), 746 (m), 731 (s), 723 (m), 687 (s), 640 (m), 623 (m), 575 (w), 542 (m), 524 (m), 501 (s).

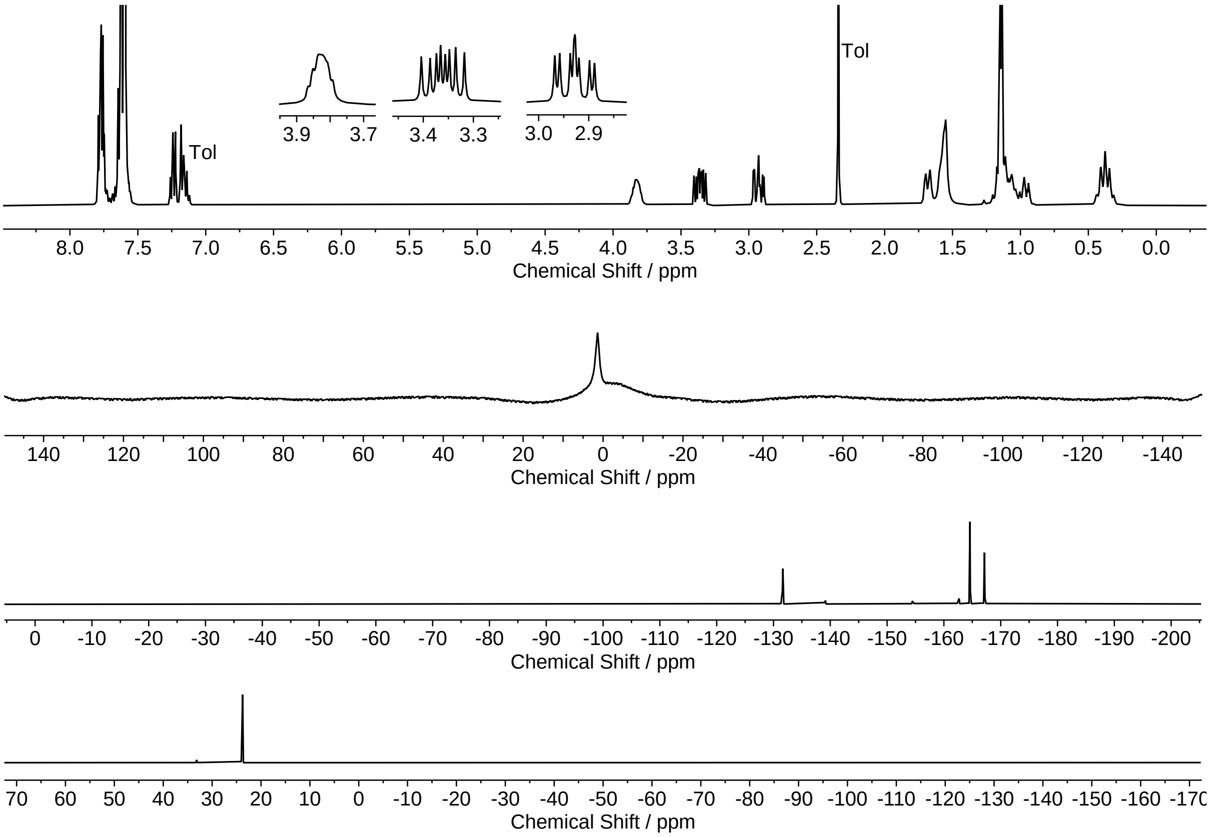

Figure S12: ^{1}H, ^{11}B, ^{19}F and ^{31}P (top to bottom) NMR spectra of **SM2**.

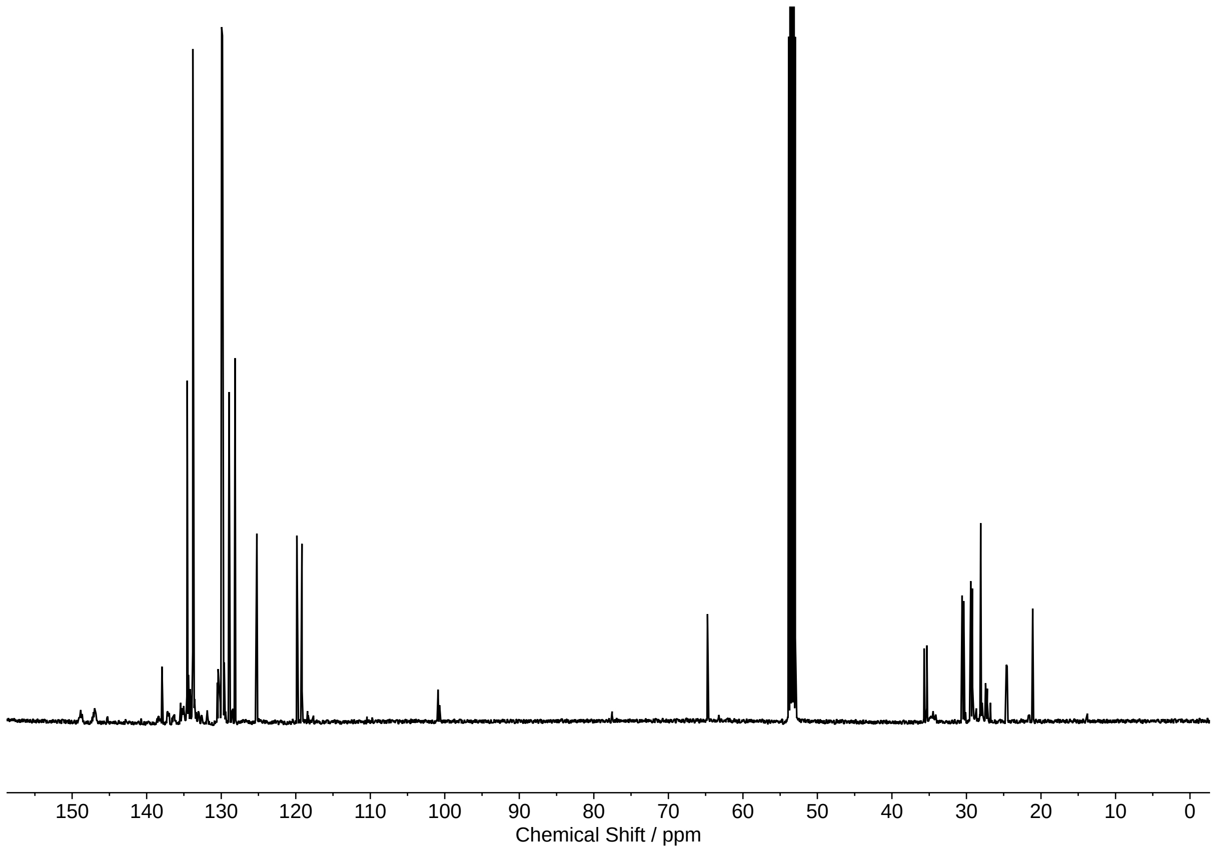

Figure S13: ^{13}C NMR spectrum of **SM2**.

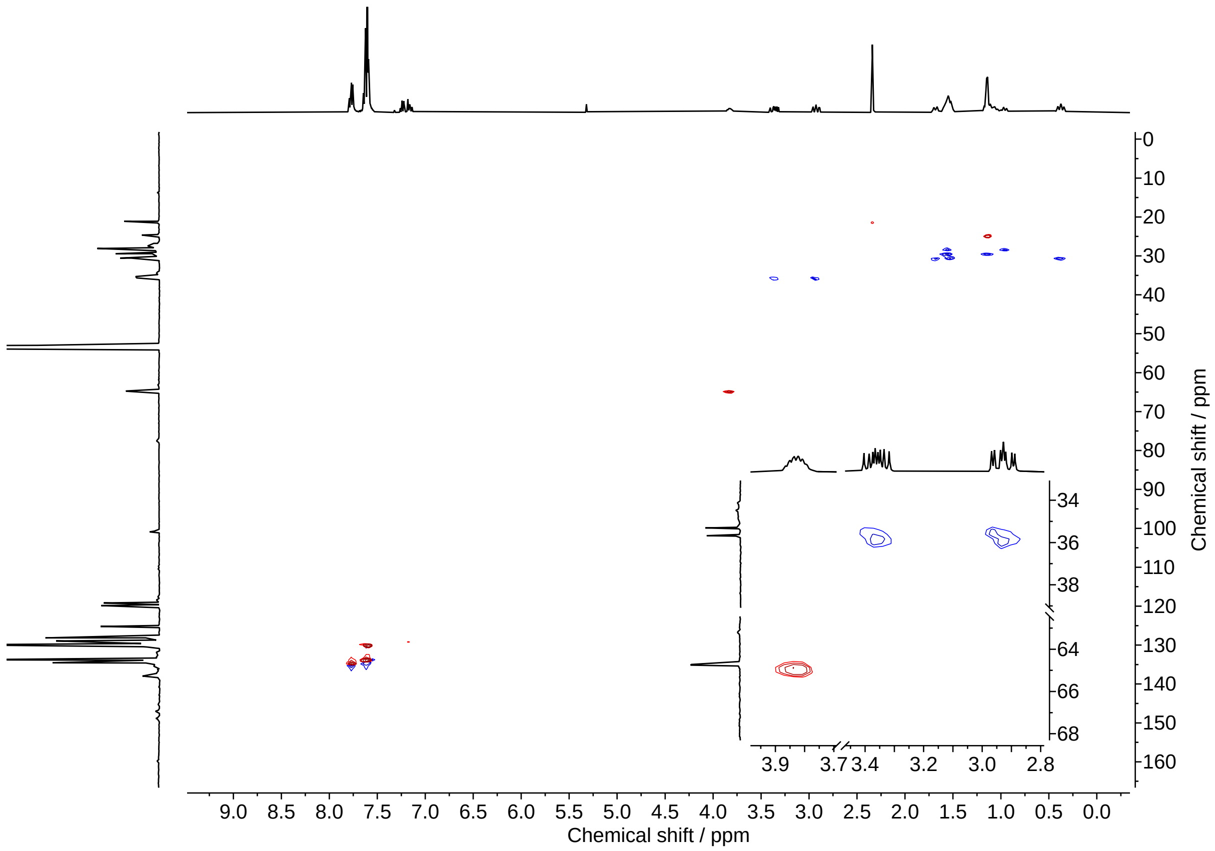

Figure S14: ^{1}H-^{13}C HSQC NMR spectrum of **SM2**.

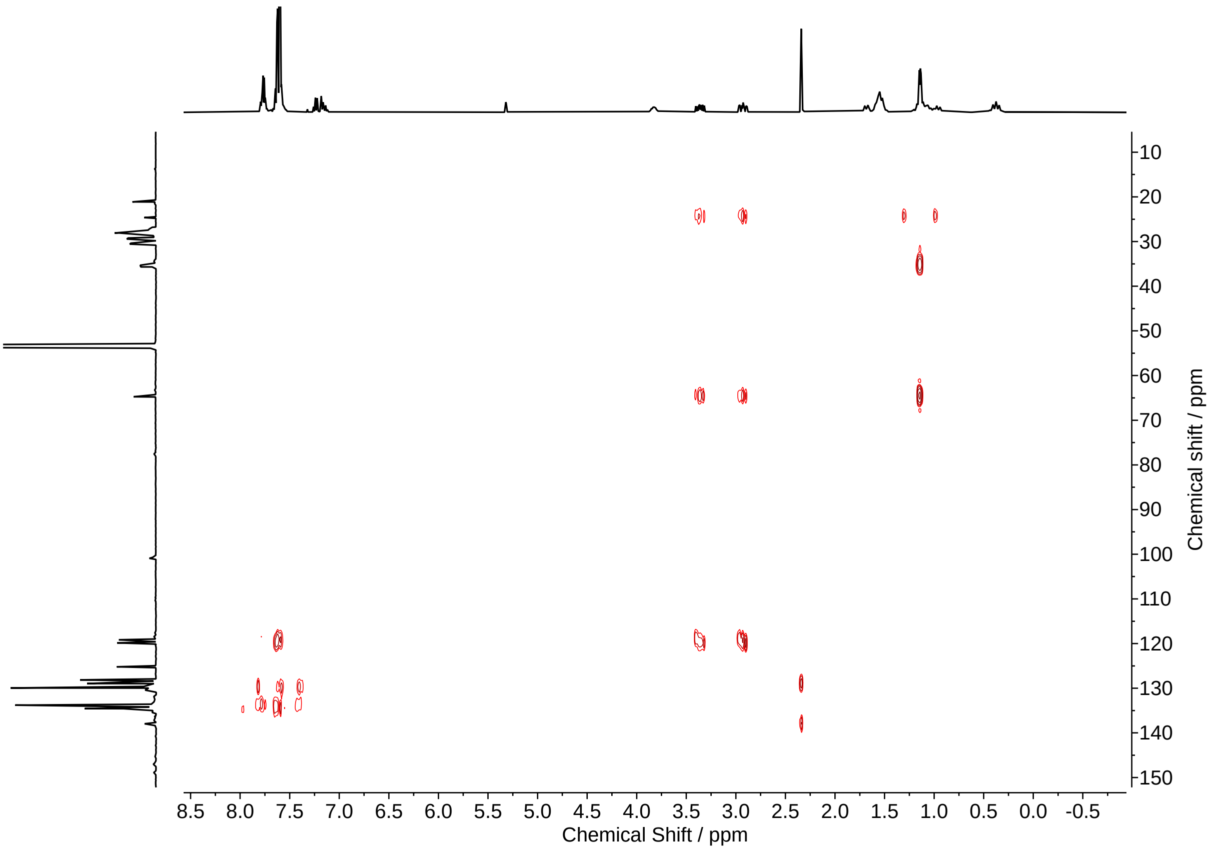

Figure S15: ^{1}H-^{13}C HMBC NMR spectrum of **SM2**.

SYN9. Synthesis of SM3

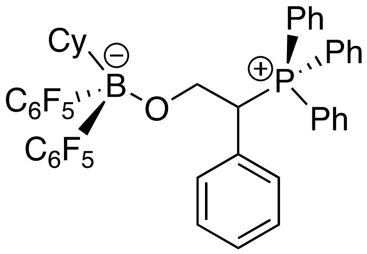

To a toluene (4 ml) solution containing $CyB(C_6F_5)_2$ (0.21 g, 0.50 mmol) and triphenylphosphine (0.13 g, 0.50 mmol), styrene oxide (60 mg, 0.50 mmol) was added at 0 °C under inert atmosphere. The mixture was stirred at this temperature for 10 mins and then allowed to warm to RT. It was stirred for another 30 mins before being poured into hexane. The crude product was collected by filtration and purified by re-dissolving in toluene and precipitating into hexane to give white colour powder in 72 % yield (0.28 g).

^{1}H NMR (400 MHz, 294 K, DCM-*d*$_2$) δ / ppm 7.78-7.66 (m, 3H, (*p*-C_6H_5)P), 7.58-7.40 (m, 12H, (*m*-C_6H_5)P, (*o*-C_6H_5)P), 7.37-7.28 (m, 1H, (*m*-C_6H_5)C) 7.28-7.10 (m, 2H, (*m*-C_6H_5)C), 7.02-6.92 (2H, *o*-C_6H_5)C), 4.61 (ddd, 1H, P-CH, $J_{H,H}$ 13, 11 Hz, $J_{P,H}$ 8.5 Hz), 3.94 (ddd, 1H, C*H*H-

O, $J_{P,H}$ 30 Hz, $J_{H,H}$ 11, 5 Hz), 3.82 (ddd, 1H, CH*H*-O, $J_{P,H}$ 19 Hz, $J_{H,H}$ 11, 8.5 Hz), 1.67-1.37, 1.36-1.01, 1.00-0.77 (m, 9H, Cy), 0.46 (q, 1H, Cy, $J_{H,H}$ 12 Hz), 0.18 (q, 1H, Cy, $J_{H,H}$ 12 Hz).

^{13}C{^{1}H} NMR (101 MHz, 294 K, DCM-*d$_2$*) δ / ppm 148.3 (dm, *o*-C_6F_5, $J_{F,C}$ 236 Hz), 137.8 (dm, *p*-C_6F_5, $J_{F,C}$ 252 Hz, 136.7 (dm, *m*-C_6F_5, $J_{F,C}$ 248 Hz), *n.o.* (*i*-C_6F_5), 135.1 (d, (*o*-C_6H_5)P, $J_{P,C}$ 10 Hz), 134.7 (d, (*p*-C_6H_5)P, $J_{P,C}$ 3 Hz), 132.4 (d, (*i*-C_6H_5)C), $J_{P,C}$ 5 Hz), 130.7 (d, (*o*-C_6H_5)C, $J_{P,C}$ 6.1 Hz), 129.9 (d, (*m*-C_6H_5)P, $J_{P,C}$ 12 Hz), 129.5 (d, (*m*-C_6H_5)C, $J_{P,C}$ 2.3 Hz), 129.4 (s, (*p*-C_6H_5)C), 118.9 (d, (*i*-C_6H_5)P, $J_{P,C}$ 84 Hz), 66.0 (d, O-CH, $J_{P,C}$ 5.2 Hz), 48.5 (d, P-CH_2, $J_{P,C}$ 44 Hz), 34.7 (br, B-CH), 31.0, 30.3, 29.9, 29.7, 28.5 (s, Cy).

^{11}B{^{1}H} NMR (128 MHz, 294 K, DCM-*d$_2$*) δ / ppm 1.86 (br, $\omega_{1/2}$ = ~160 Hz).

^{19}F NMR (376 MHz, 294 K, DCM-*d$_2$*) δ / ppm -130.6 (br, 2F, *o*-C_6F_5), -132.9 (br, 2F, *o*-C_6F_5), -163.2 (t, 1F, *p*-C_6F_5, $J_{F,F}$ 21 Hz), -164.4 (t, 1F, *p*-C_6F_5, $J_{F,F}$ 21 Hz), -166.3 (m, 2F, *m*-C_6F_5), -166.9 (m, 2F, *m*-C_6F_5).

^{31}P{^{1}H} NMR (162 MHz, 294 K DCM-*d$_2$*) δ / ppm 26.7 (s).

HR-ESI-MS calculated for $C_{44}H_{34}BF_{10}OP$ $[M+Na]^+$ 833.2178, found 833.2172.

IR (ATR) cm^{-1} 3062 (w), 3029 (w), 2913 (w), 2840 (w), 1641 (w), 1600 (w), 1588 (w), 1508 (m), 1496 (w), 1484 (w), 1437 (s), 1385 (w), 1265 (m), 1207 (w), 1108 (m), 1072 (s), 1028 (w), 998 (w), 964 (s), 948 (m), 923 (m), 906 (w), 879 (w), 844 (w), 808 (w), 788 (w), 771 (w), 746 (m), 727 (m), 684 (s), 632 (w), 613 (w), 576 (w), 551 (w), 528 (m), 507 (m).

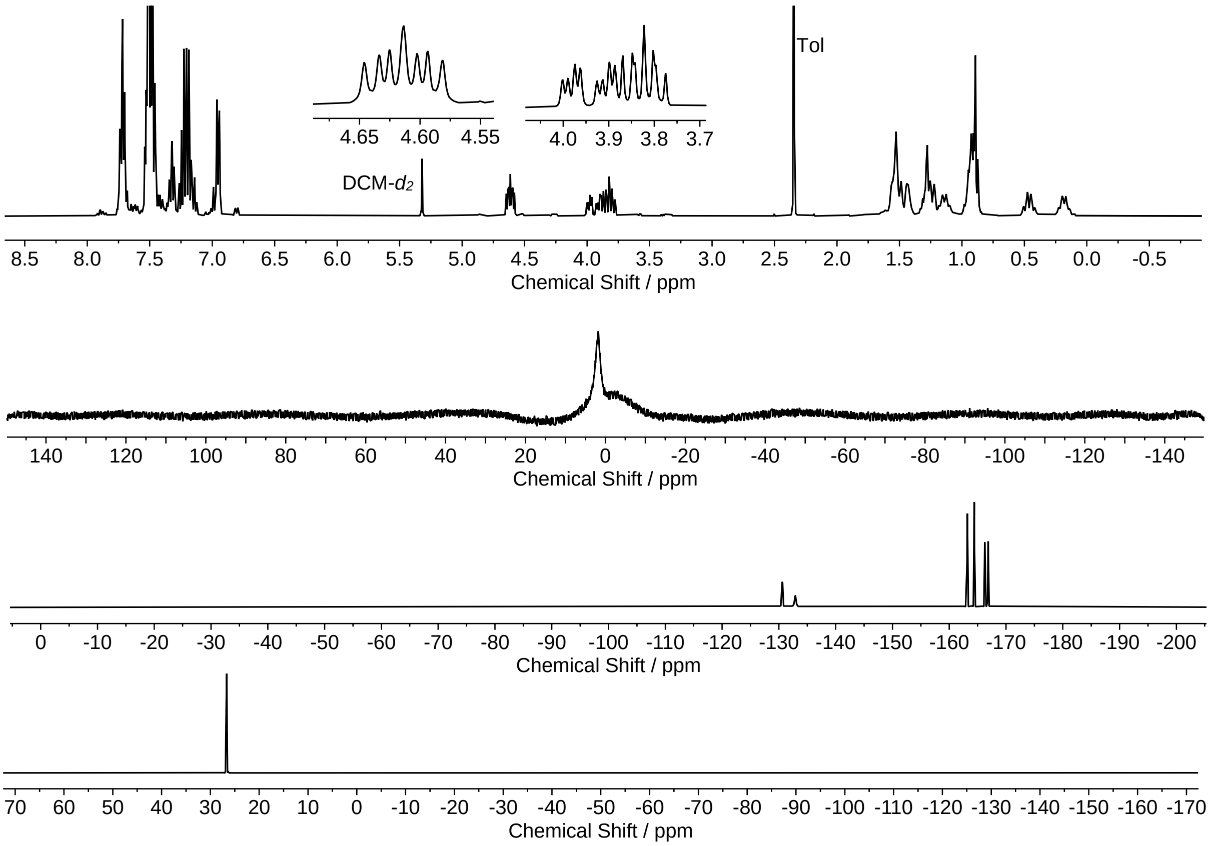

Figure S16: ^{1}H, ^{11}B, ^{19}F and ^{31}P (top to bottom) NMR spectra of **SM3**.

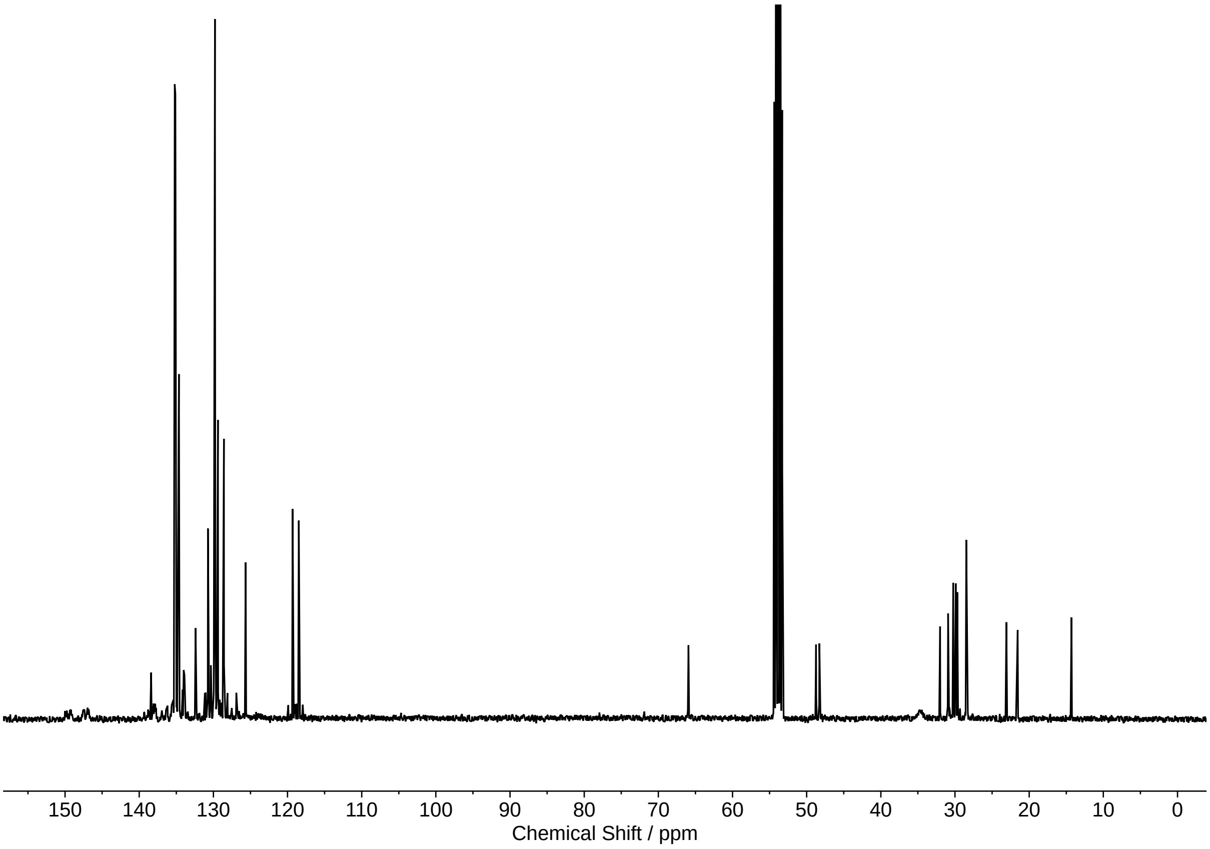

Figure S17: ^{13}C NMR spectrum of **SM3**.

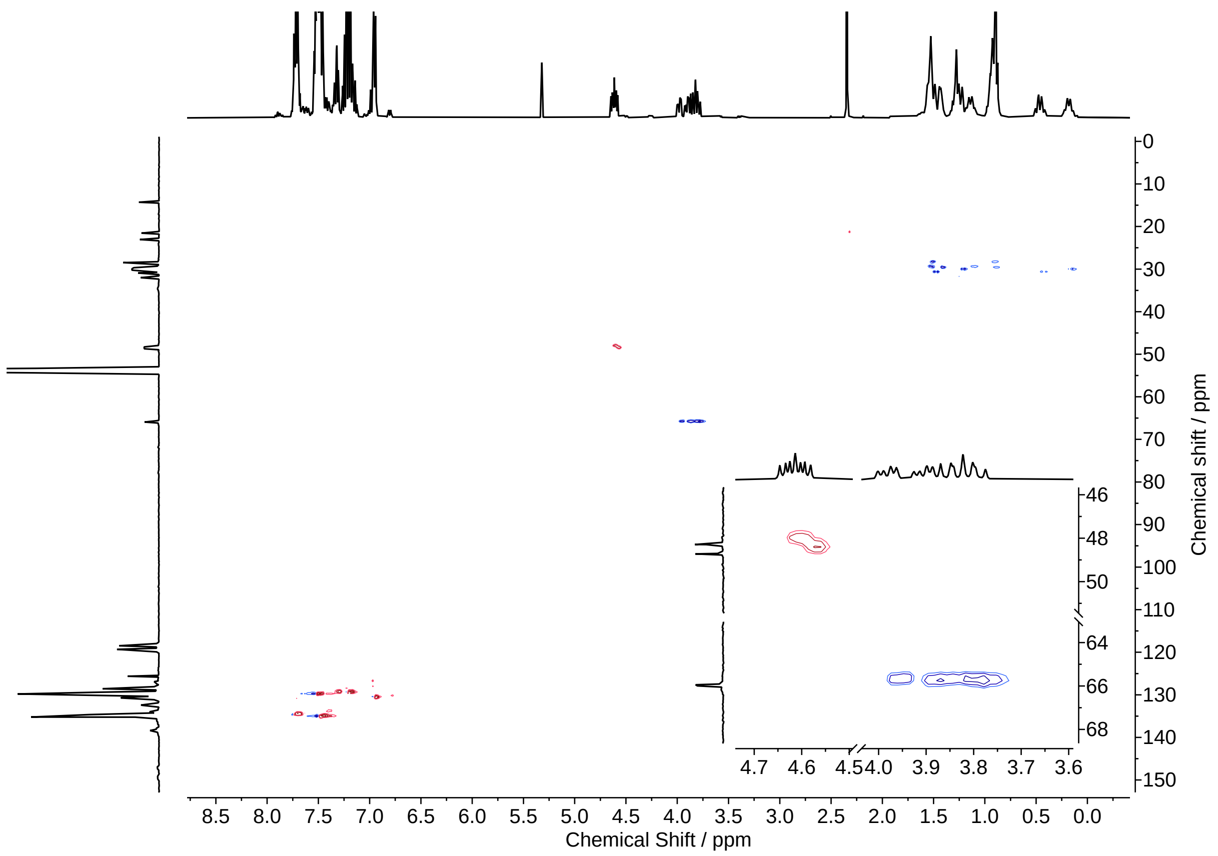

Figure S18: ^{1}H-^{13}C HSQC NMR spectrum of **SM3**.

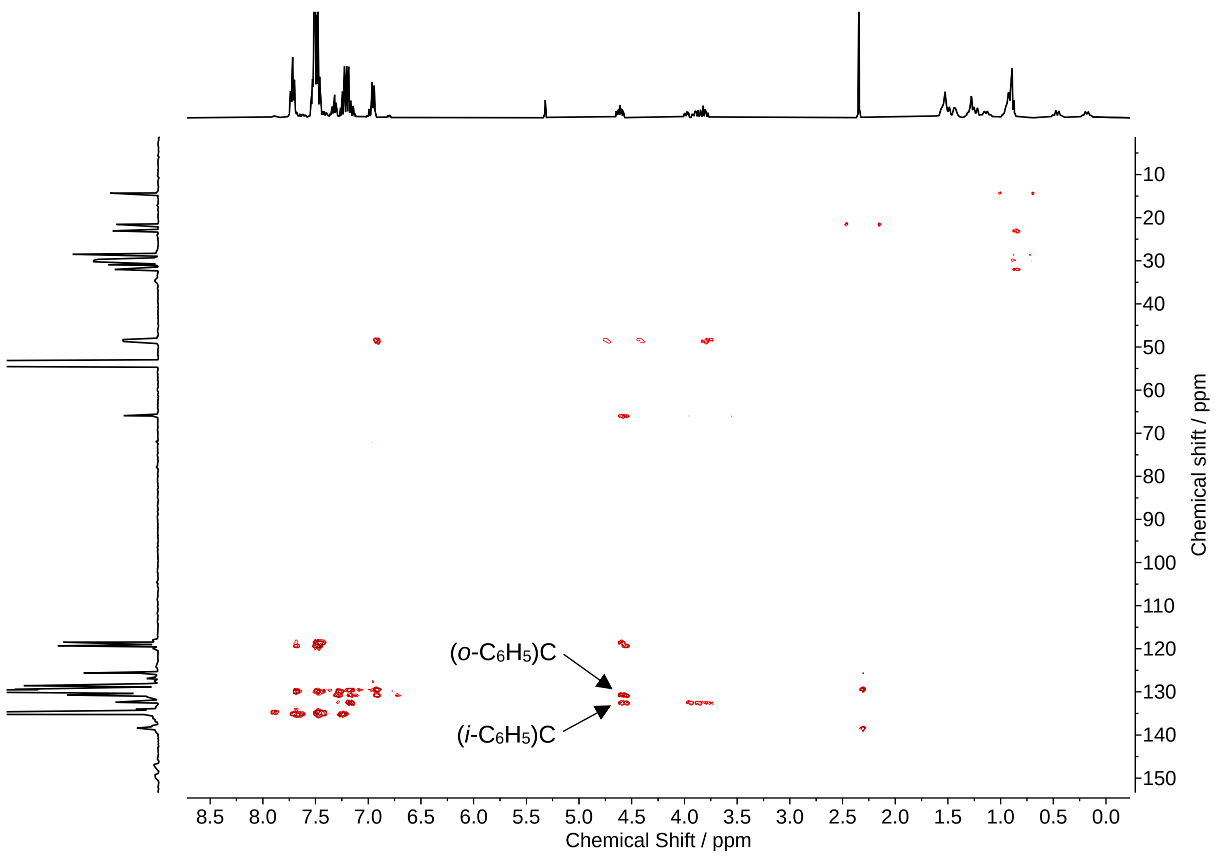

Figure S19: ^{1}H-^{13}C HMBC NMR spectrum of **SM3**.

SYN10. Synthesis of SM4

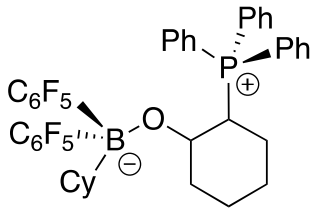

To a toluene (4 ml) solution containing $CyB(C_6F_5)_2$ (0.20 g, 0.47 mmol) and triphenylphosphine (0.12 g, 0.47 mmol), cyclohexene oxide (46 mg, 0.47 mmol) was added at 0 °C under inert atmosphere. The mixture was stirred at this temperature for 10 mins and then allowed to warm to RT. It was stirred for another 30 mins before being poured into hexane. The crude product was collected by filtration and purified by re-dissolving in toluene and precipitating into hexane to give a white foam in 83% yield (0.31 g).

^{1}H NMR (400 MHz, 294 K, DCM-*d₂*) δ / ppm 7.78-6.31 (m, 9H, *p*-C_6H_5, *o*-C_6H_5), 7.58 (td, 6H, *m*-C_6H_5, $J_{H,H}$ 7.8, 3.4 Hz), 3.46-3.22 (m, 4H, O-CH, P-CH), 2.52-2.39 (m, 1H, CyO), 1.78-1.53, 1.52-1.37, 1.36-0.80 (m, 14H, CyO, CyB), 0.70-0.54, 0.53-0.39, 0.39-0.24, 0.13- -0.03 (m, 4H, CyB).

^{13}C{^{1}H} NMR (101 MHz, 294 K, DCM-d_2) δ / ppm 148.3 (dm, *o*-C_6F_5, $J_{F,C}$ 239 Hz), n.o. (*p*-C_6F_5), 136.5 (dm, *m*-C_6F_5, $J_{F,C}$ 242 Hz), *n.o.* (*i*-C_6F_5), 134.8 (d, *o*-C_6H_5, $J_{P,C}$ 10 Hz), 134.3 (d, *p*-C_6H_5, $J_{P,C}$ 3 Hz), 129.9 (d, *m*-C_6H_5, $J_{P,C}$ 12 Hz), 120.8 (d, br, *i*-C_6H_5, $J_{P,C}$ 84 Hz), 74.9 (d, O-CH, $J_{P,C}$ 5.8 Hz), 44.4 (d, P-CH, $J_{P,C}$ 45 Hz), 35.8 (br, B-CH), 35.2 (d, CyO, $J_{P,C}$ 11 Hz), 31.6, 31.5, 29.8, 29.6, 28.4 (s, CyB), 30.2 (d, CyO, $J_{P,C}$ 3.6 Hz), 27.5 (d, CyO, $J_{P,C}$ 14 Hz), 25.0 (d, CyO, $J_{P,C}$ 1.0 Hz).

^{11}B{^{1}H} NMR (128 MHz, 294 K, DCM-d_2) δ / ppm 0.93 (br, $\omega_{1/2}$ = ~140 Hz).

^{19}F NMR (376 MHz, 294 K, DCM-d_2) δ / ppm -128.7 (br, 4F, *o*-C_6F_5), -163.3 (t, 1F, *p*-C_6F_5, $J_{F,F}$ 21 Hz), -165.4 (t, 1F, *p*-C_6F_5, $J_{F,F}$ 21 Hz), -166.2 (br, 2F, *m*-C_6F_5), 167.9 (m, 2F, *m*-C_6F_5).

^{31}P{^{1}H} NMR (162 MHz, 294 K DCM-d_2) δ / ppm 29.4 (s).

HR-ESI-MS calculated for $C_{42}H_{36}BF_{10}OP$ $[M+Na]^+$ 811.2335, found 811.2320.

IR (ATR) cm^{-1} 3062 (w), 2913 (w), 2838 (w), 1645 (w), 1510 (m), 1485 (w), 1448 (s), 1434 (s), 1386 (w), 1375 (w), 1282 (w), 1268 (w), 1193 (w), 1105 (m), 1092 (m), 1074 (m), 1027 (w), 981 (m), 966 (s), 919 (m), 895 (w), 870 (w), 856 (w), 842 (w), 808 (w), 796 (w), 784 (w), 773

(m), 746 (m), 732 (m), 713 (m), 692 (s), 667 (m), 657 (m), 631 (w), 619 (w), 575 (w), 566 (w), 540 (m), 525 (m), 507 (s).

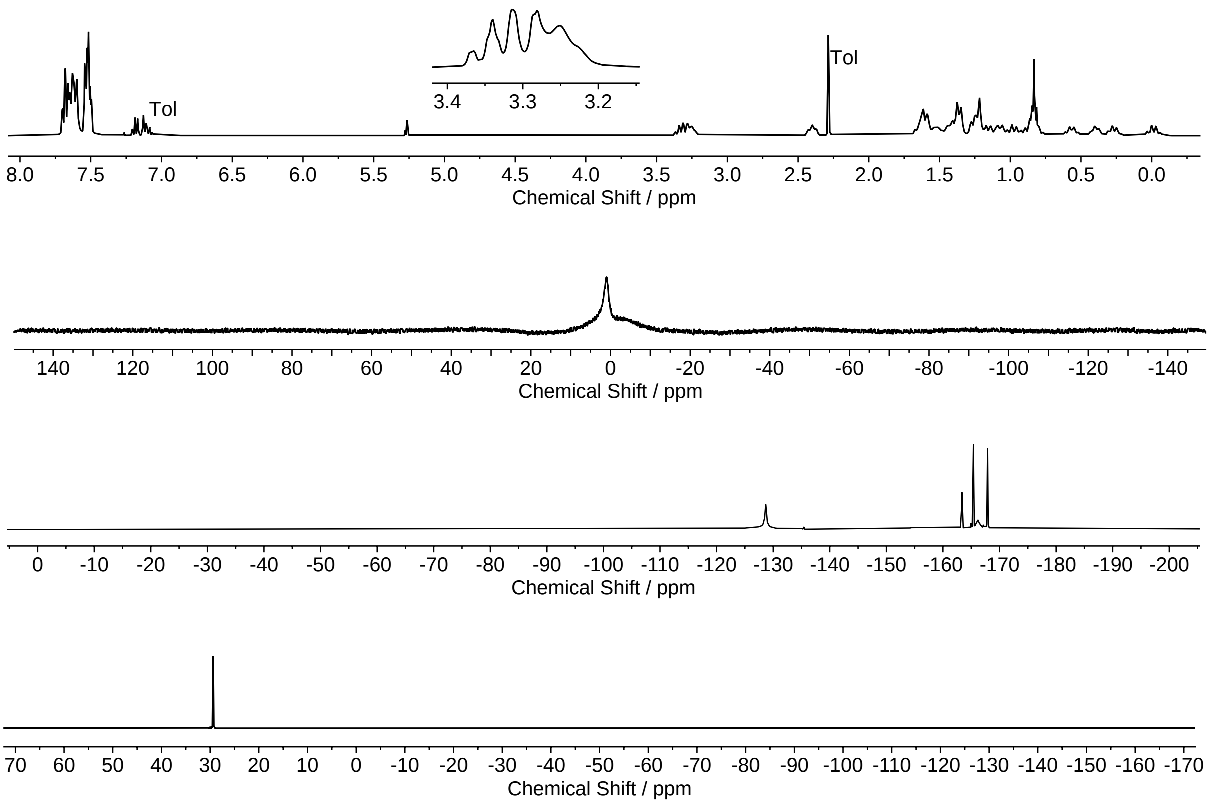

Figure S20: ^{1}H, ^{11}B, ^{19}F and ^{31}P (top to bottom) NMR spectra of **SM4**.

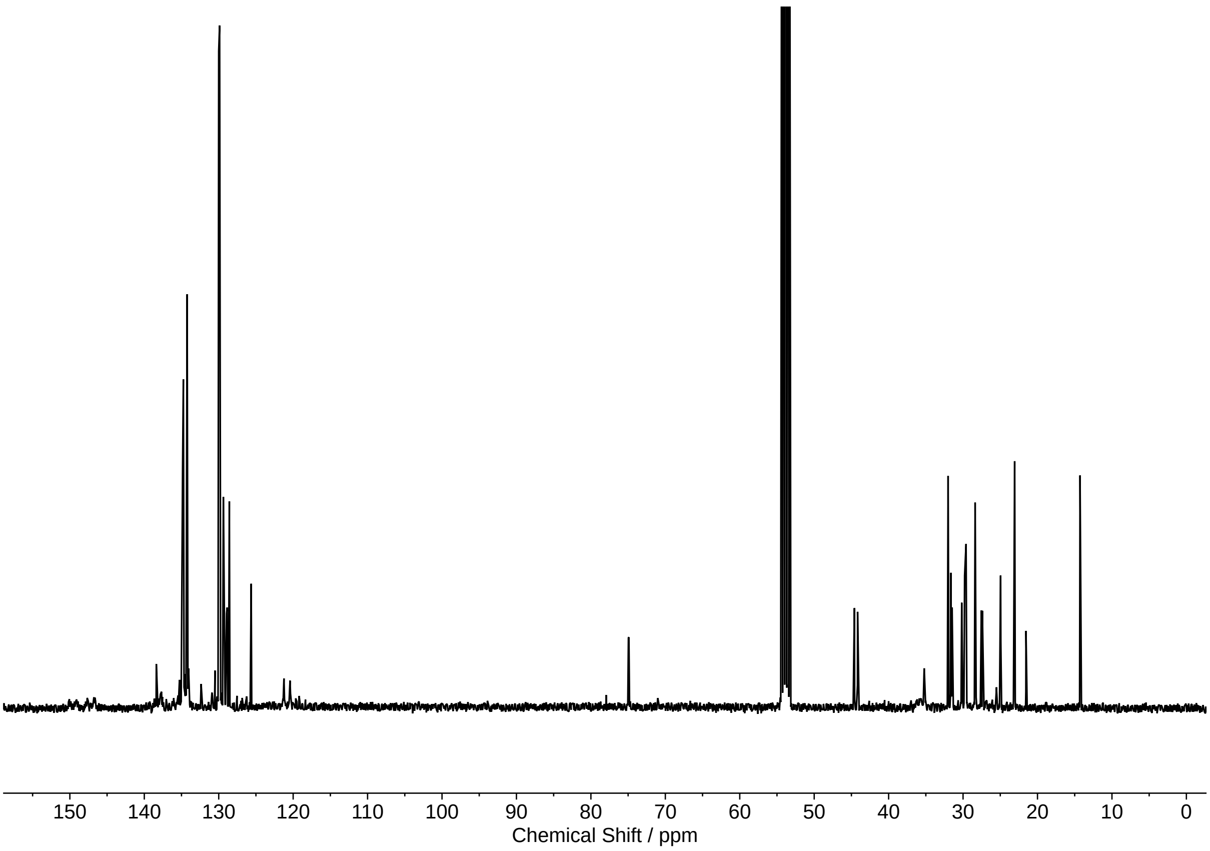

Figure S21: ^{13}C NMR spectrum of **SM4**.

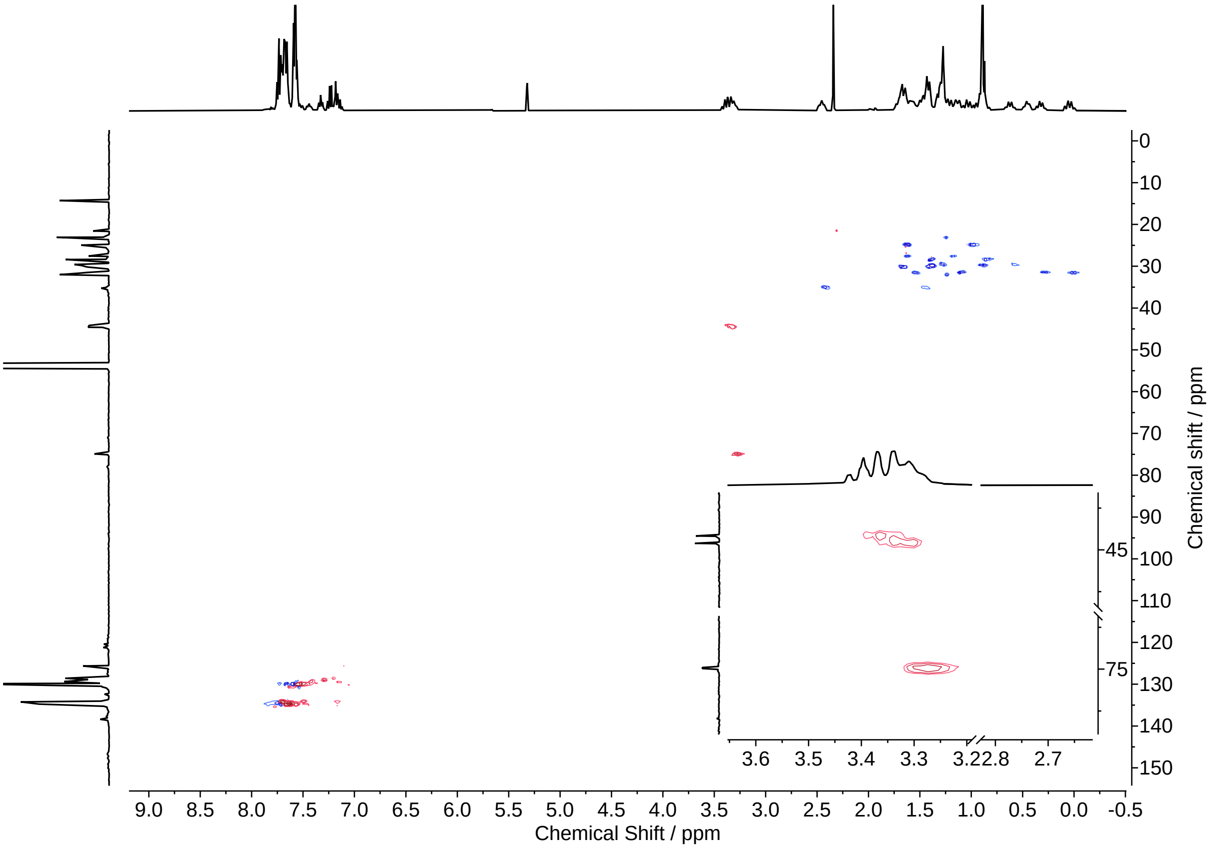

Figure S22: ^{1}H-^{13}C HSQC NMR spectrum of **SM4**.

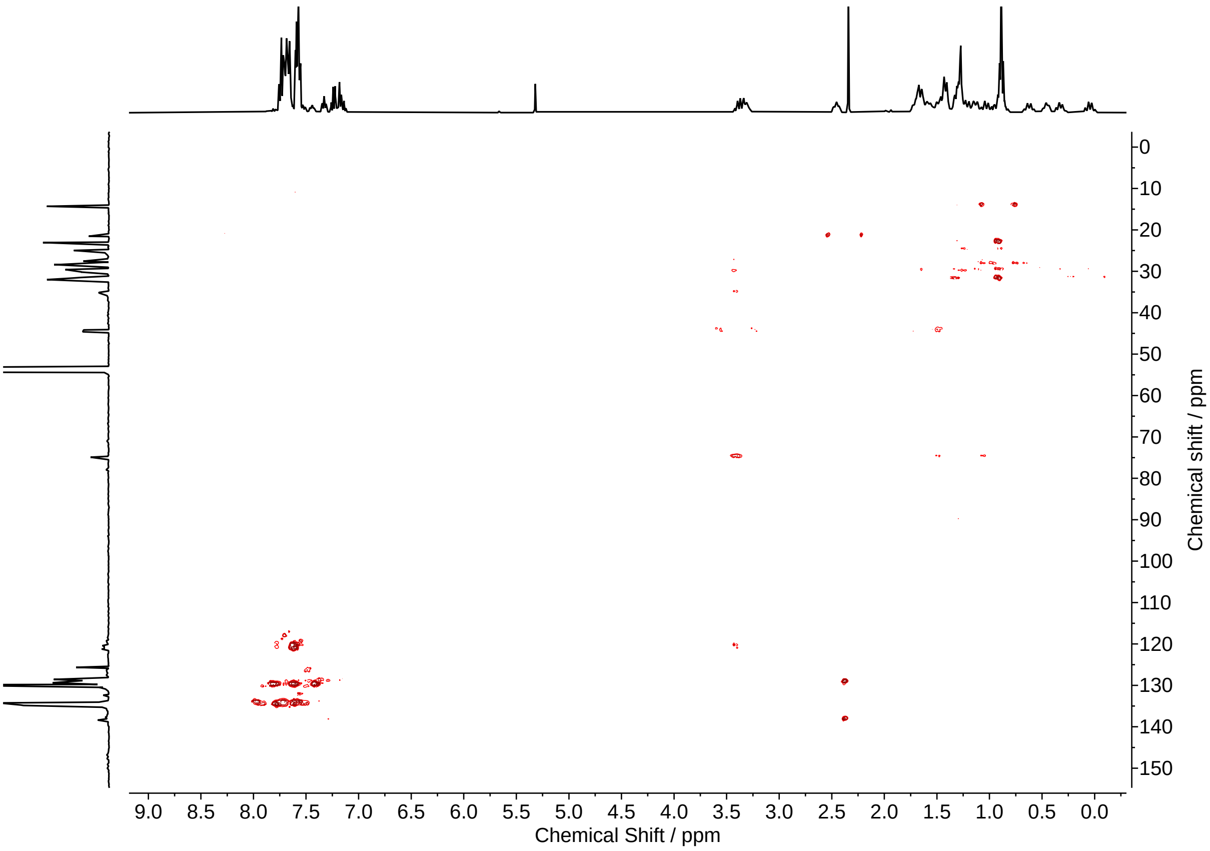

Figure S23: ^{1}H-^{13}C HMBC NMR spectrum of **SM4**.

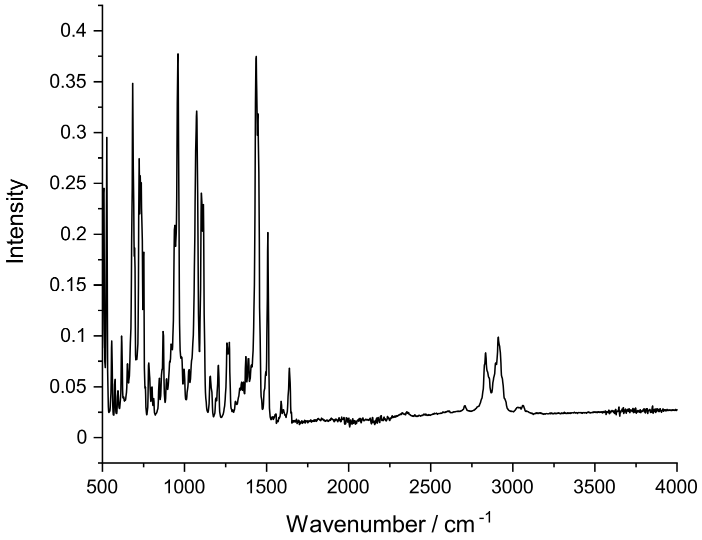

Figure S24: FTIR spectrum of **SM1**.

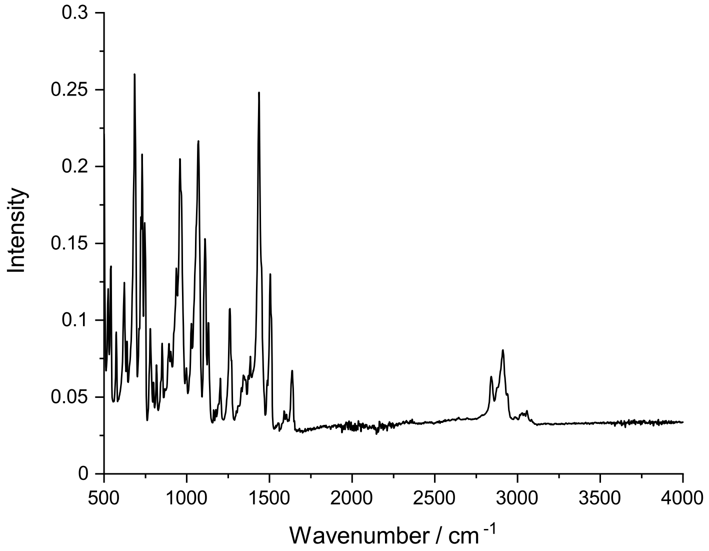

Figure S25: FTIR spectrum of **SM2**.

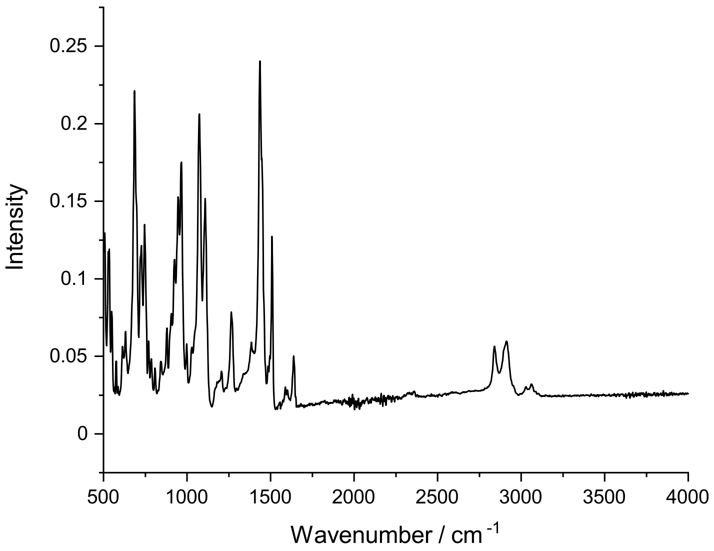

Figure S26: FTIR spectrum of **SM3**.

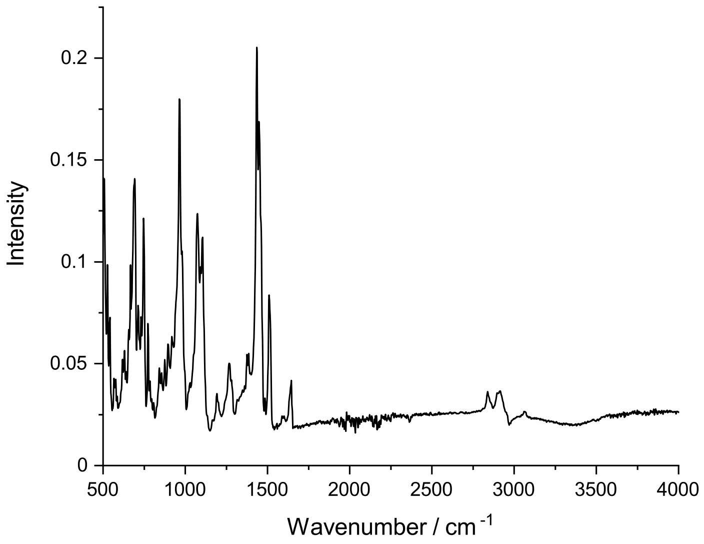

Figure S27: FTIR spectrum of **SM4**.

Table S2: ^{11}B, ^{31}P and ^{19}F NMR chemical shifts for $CyB(C_6F_5)_2$, PPh_3, **SM1**, **SM2**, **SM3** and **SM4**.

Chemical	δ(^{11}B) / ppm	δ(^{31}P) / ppm	δ(^{19}F) / ppm
$CyB(C_6F_5)_2$[a]	75.3 ($\omega_{1/2}$ ~570 Hz)	-	-130.1, -149.1, -160.9
PPh_3[a]	-	-5.4	-
**SM1**[b]	1.77 ($\omega_{1/2}$ ~100 Hz)	23.9	-131.5, -164.6, -167.1
**SM2**[b]	1.38 ($\omega_{1/2}$ ~140 Hz)	23.8	-131.6, -164.6, -167.3
**SM3**[b]	1.86 ($\omega_{1/2}$ ~160 Hz)	26.7	-130.6 (br), -132.9 (br), -163.2, -164.4, -166.3, -166.9
**SM4**[b]	0.93 ($\omega_{1/2}$ ~140 Hz)	29.4	-128.7 (br), -163.3, -165.4, -166.2 (br), -167.9

[a]NMR spectra obtained in $CDCl_3$. [b]NMR spectra obtained in CD_2Cl_2.

NMR Characterization of Product Connectivity

As X-ray quality crystals could not be obtained from these structures, NMR studies were used to correlate to preexisting structures to aid structural determination. Thanks to the works of Slootweg, Stephan and their co-workers, a library of FLP-mediated ring-opened cyclic ethers

is shown in Figure S28. All the displayed zwitterions were specifically chosen as they were characterized using X-ray diffraction in their reported publications.[5-8] When an in-depth investigation of their NMR spectra was performed, a strong relationship between the coupling constants $^{1}J_{C,P}$ and $^{2}J_{C,P}$ (of P-RO-B groups, the carbon atoms of interest are labelled as red and green below, respectively) was found. This link is also shown in Table S3, where the carbon atom directly attached to the phosphine center has 5 to 10 times stronger coupling constants than the carbon atoms two bonds away from the P centers. This is also in agreement with the expectation that the single bond coupling would dramatically increase with the loss of phosphine lone pair.[9] Considering the strong literature precedent, the magnitudes of the coupling constants are likely diagnostic for the carbon atom bonded to P.

Slootweg 2018

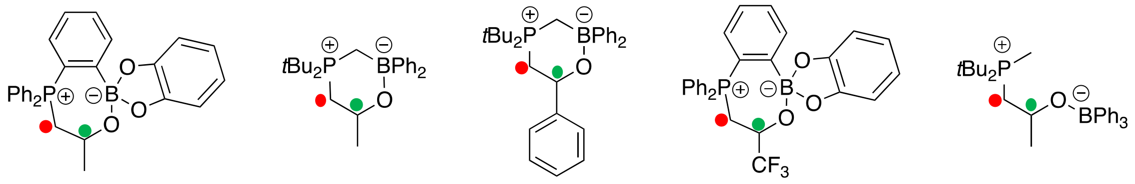

1 **2** **3** **4** **5**

Stephan 2009, 2010 and 2011

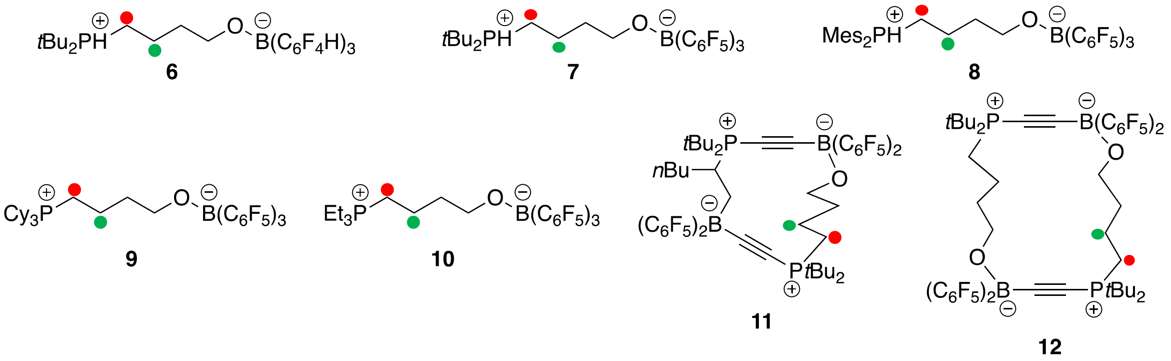

Figure S28: XRD-characterized zwitterions prepared by the FLP-mediated ring-opening reactions of cyclic ethers. Red circles represent the carbon atoms directly attached to the PPh_3 moieties and green circles represent the carbon atoms two bonds away from the same moiety.[5-8]

Table S3: A summary of the ^{31}P-^{13}C NMR coupling constants reported for the carbon atoms of interest.[5-8]

Compound	$^{1}J_{C,P}$ / Hz	$^{2}J_{C,P}$ / Hz	Compound	$^{1}J_{C,P}$ / Hz	$^{2}J_{C,P}$ / Hz	Compound	$^{1}J_{C,P}$ / Hz	$^{2}J_{C,P}$ / Hz
1	58.9	6.8	5	42.3	6.0	9	44	singlet
2	48.4	4.9	6	38.5	6.5	10	47	7
3	45.3	4.3	7	39	multiplet	11	49	10
4	64.3	6.1	8	58	singlet	12	48.7	6.4

Strong $^{1}J_{C,P}$ couplings (P-RO-B) were observed with all the small molecule mimics in their ^{13}C NMR spectra - 54 Hz (**SM1**), 47 Hz (**SM2**), 44 Hz (**SM3**) and 45 Hz (**SM4**). The ^{13}C resonances were then matched with the respective CH_2 or CH/CH_3 protons using DEPT-edited HSQC (Figure S29). The P center attacks at the less sterically crowded carbon atom for **SM2** and at the more sterically hindered carbon for **SM3**. As seen in Figure S29, the strongly phosphine coupled ^{13}C resonances are directly bonded to CH_2 and CH groups for **SM2** and **SM3**, respectively. This could happen if the aromatic ring stabilizes any induced +ve in electron density, by lowering down the energy at transition state and increasing the positive charge on the benzyl carbon. The only other FLP-mediated ring-openings of styrene oxides were reported by Slootweg and co-workers.[5] where P centers attacking at the less sterically crowded carbon atoms. We attribute these differences to the intramolecular nature of the FLP systems and the relatively lower electrophilicities of the Lewis acids (having phenyl and catechol substituents instead of the strongly electron-withdrawing C_6F_5 groups compared to our reported system). With **SM2** (**S.PO**), P attacked to the less sterically hindered carbon atom, likely because it lacks the inductive effects of a phenyl group. The electrophilicity of a LA can directly affect the LA-catalyzed ring-openings of the epoxide rings.[10]

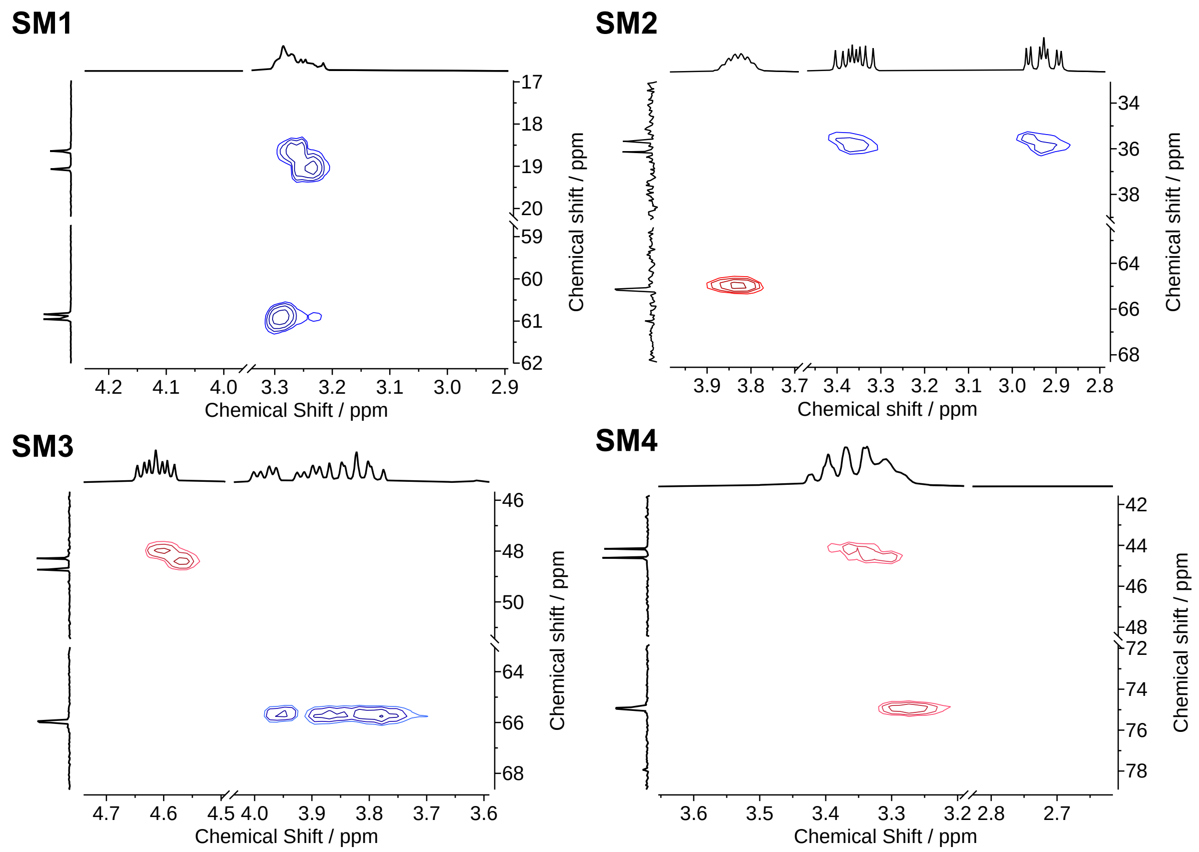

Figure S29: P-RO-B section of ^{1}H-^{13}C DEPT HSQC NMR spectra for all the small molecule mimics. Blue is for CH_2 and red is for CH and CH_3 groups.

Network Formation and Characterisation

General procedure for the preparation of polymeric FLP networks

Two separate solutions of poly(Sty-*co*-PPh_3) (0.12 g, 5.2 μmol) and poly(Sty-*co*-CyB$(C_6F_5)_2$) (0.10 g, 5.6 μmol) were prepared in toluene (or benzene, each 0.50 ml). The ratio was adjusted to have 1:1 equivalent of LA and LB moieties based on the data provided in Table 1. These solutions were kept inside the glovebox freezer (-35 °C) for storage. The polymer stock solutions were combined at ambient temperature prior to gelation and the containing vial was shaken to ensure true mixing. Pre-diluted cyclic ether substate in toluene (or benzene, 0.25 ml, containing 2.5 equivalents linker) was then injected into the vial resulting in an immediate gelation reaction. Total polymer concentration was kept at 0.18 g ml^{-1}. The resultant polymer network was allowed to stand at RT overnight.

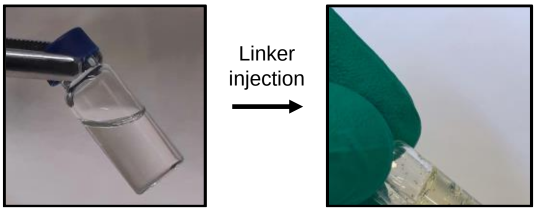

Figure S30: Preparation of the polymeric FLP gels inside vials (above example using PO as the crosslinker).

Gel fractions and swelling ratio of the polymeric networks

Polymer networks were prepared in benzene and flash-frozen in liquid nitrogen for 5 minutes prior to freeze-drying under high vacuum. About 50 mg of each xerogel was weighed into a vial and the dried samples were submerged in and washed with benzene. As the polymer network became swollen, supernatant benzene was constantly removed and re-placed. Changes in the weights of the samples were recorded until they stabilized. Excess benzene was then removed, and the sample was re-dried under vacuum to remove all the solvent. Changes in weights of the samples were recorded and used for the calculation of gel fractions.

Equation S1: Definition of the gel fraction.

$$Gel\ fraction = Weight\ of\ the\ dried\ crosslinked\ sample\ after\ benzene\ washes / Initial\ weight\ of\ the\ sample$$

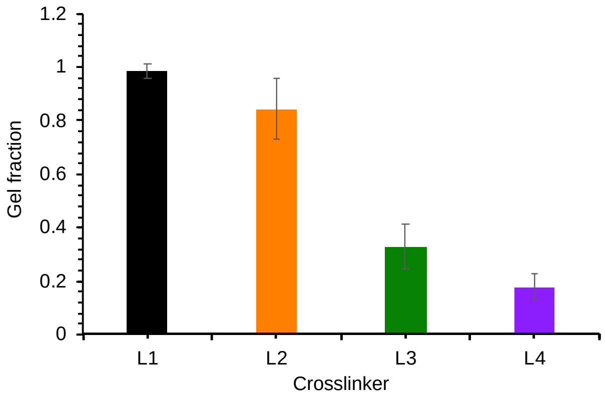

Figure S31: A plot to demonstrate how gel fraction changes with various crosslinkers.

About 50 mg of each washed and dried xerogels was weighed in a vial and submerged in toluene. Excess toluene was removed at regular intervals and the weight of the swollen polymer gels were recorded. This was continued until the measured weight became stable.

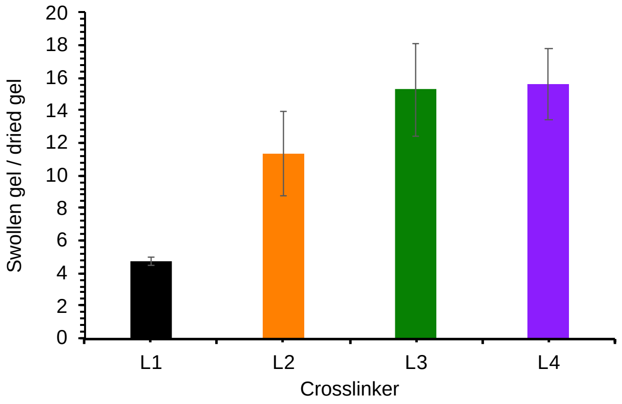

Figure S32: A plot to demonstrate how swelling ratio changes with various crosslinkers.

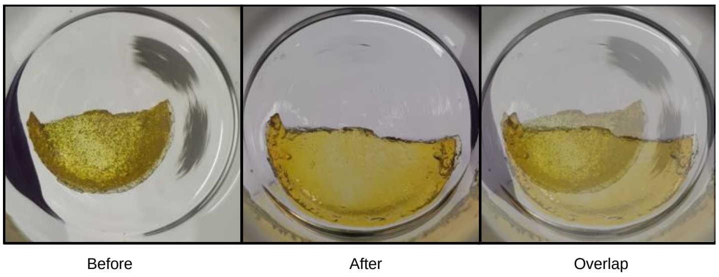

Figure S33: A series of pictures to show the extent of the polymer network swelling. Initial photo shows an OX-crosslinked polymer gel partially swollen in toluene. Upon submerging the gel into toluene overnight, the gel becomes fully swollen (middle photo). The overlap of the two photos shows the extent of the swelling where the polymer network also retains its shape.

ATR-FTIR spectra of the xerogels

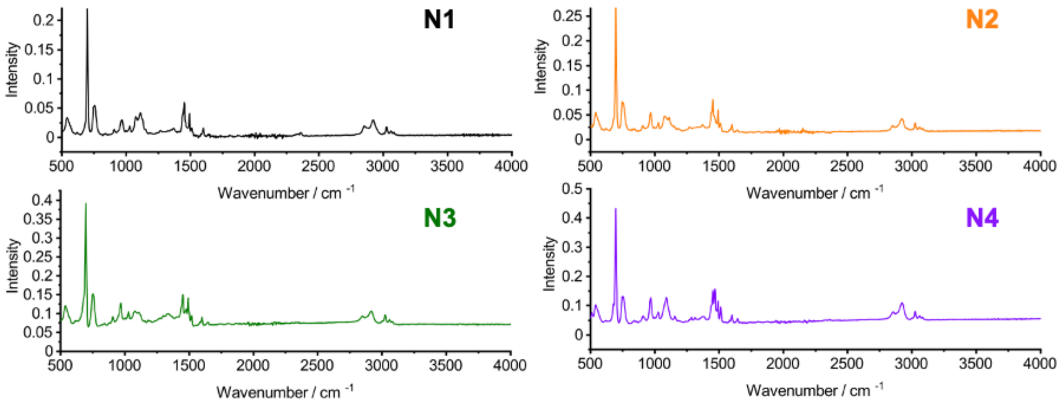

Figure S34: FTIR spectra of the polymeric LA and LB as well as the prepared dried xerogels.

Polymer network preparation for SEM

Polymer networks swollen in benzene were shock-frozen in liquid nitrogen for 5 minutes prior to freeze-drying under high vacuum. Samples were mounted on copper tapes on 12.5 mm SEM stubs. All samples were coated with silver films prior to characterisation. Samples were kept under inert atmosphere until coating and stored inside a vacuum desiccator after the coating procedure.

SEM Images

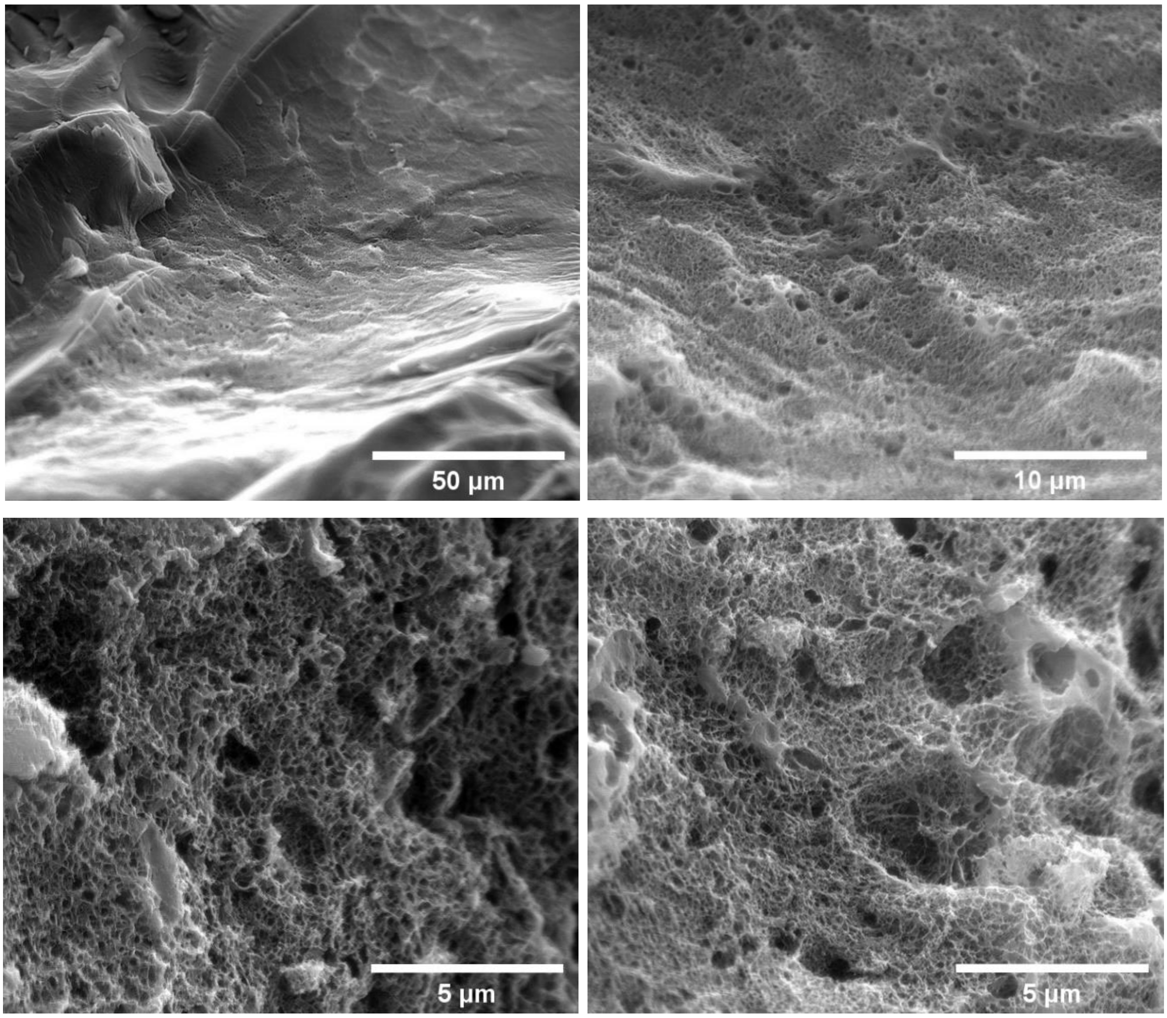

Figure S35: SEM images showing the microstructure of the freeze-dried **N1**.

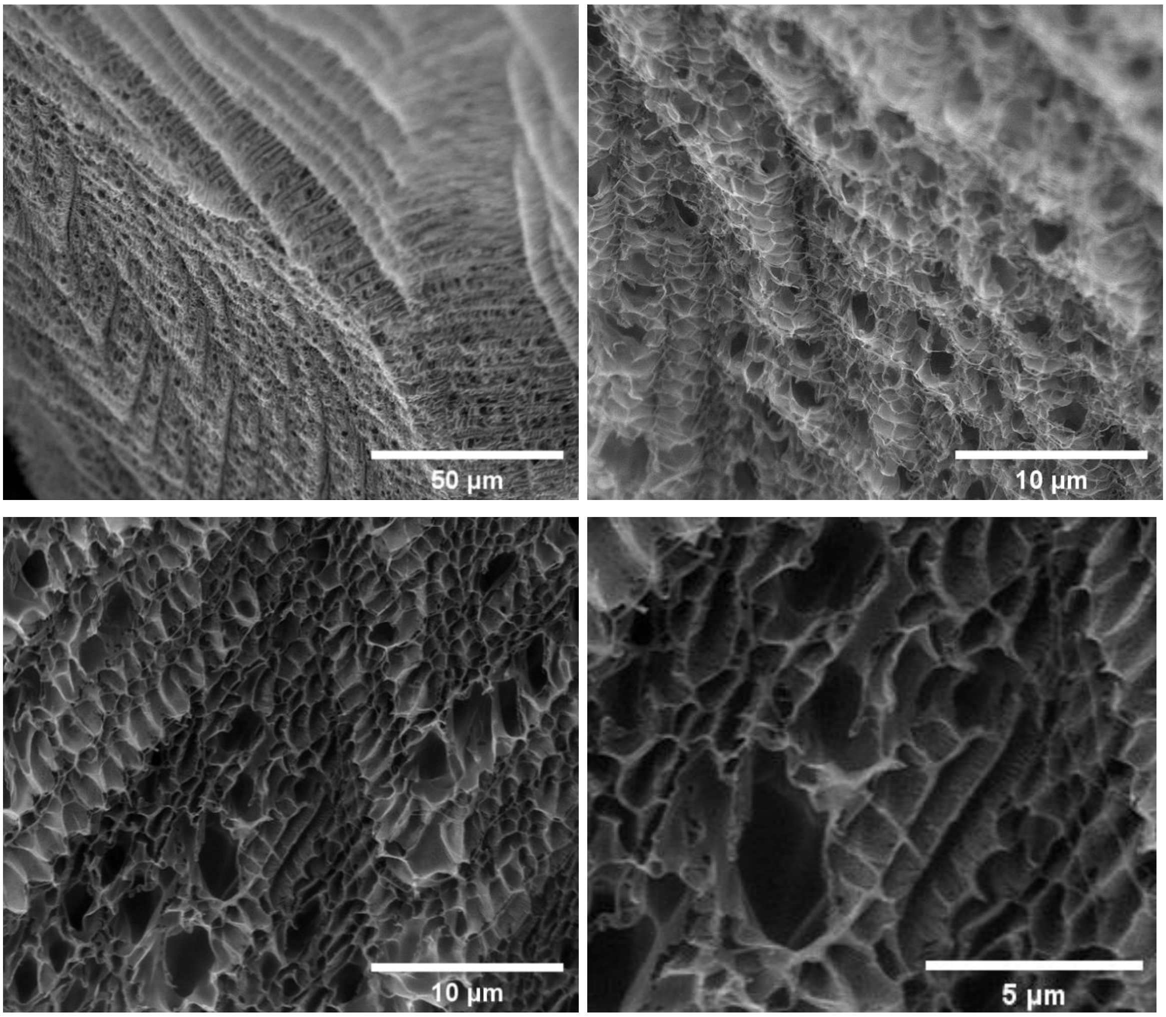

Figure S36: SEM images showing the microstructure of the freeze-dried **N2**.

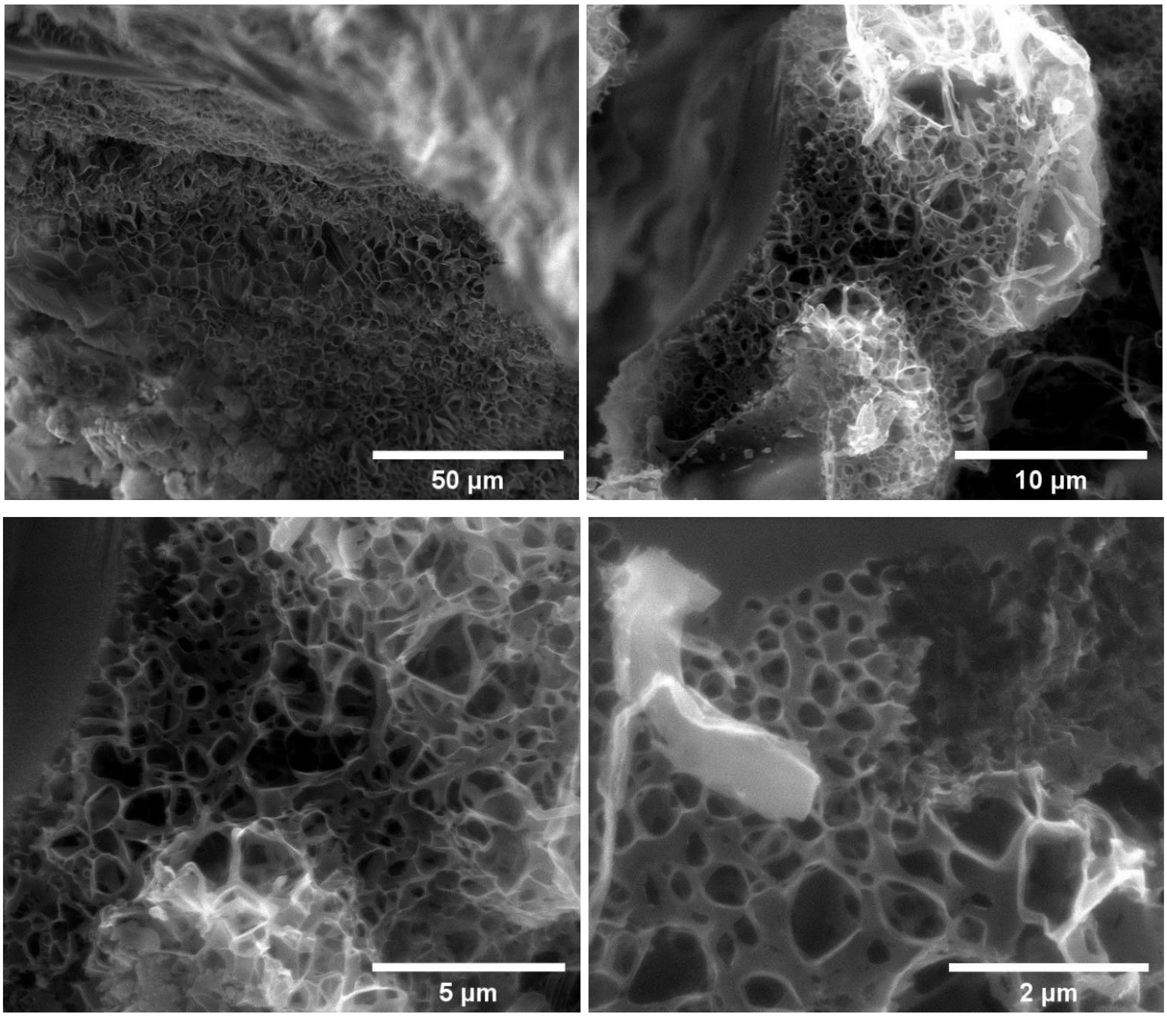

Figure S37: SEM images showing the microstructure of the freeze-dried **N3**.

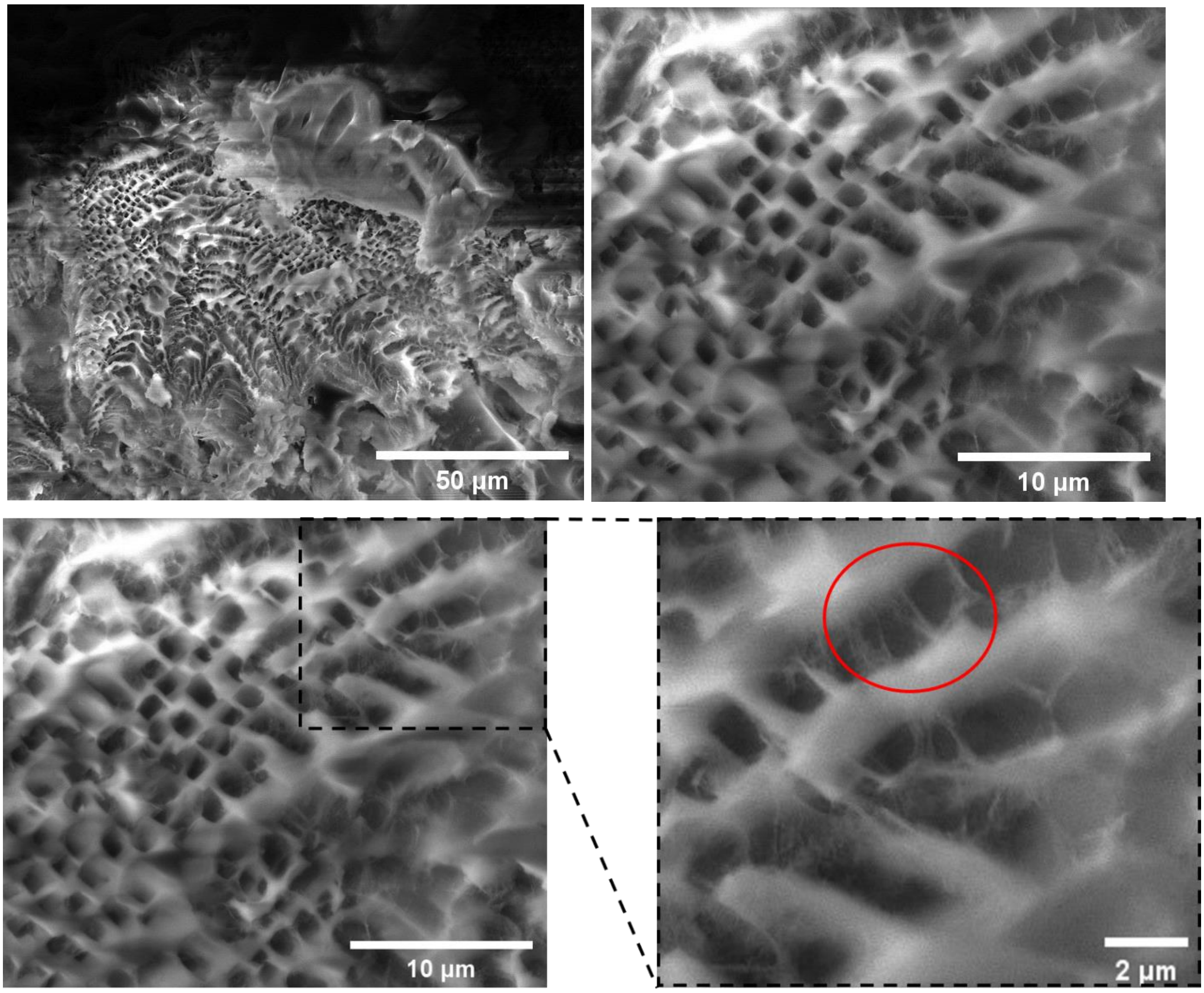

Figure S38: SEM images showing the microstructure of the freeze-dried **N4** with the bottom zoomed image highlighting (red circle) the thin and fragile polymer mesh.

Polymer network preparation for rheological characterisation

Removing polymer gels from the vials as single pieces proved challenging, and they often crumbled into many pieces. To overcome this issue, gelation reactions were performed in syringes inside a glovebox (**Figure S39**). A similar method was adopted to the earlier mentioned general procedure. All rheological characterisations were performed using polymer

networks swollen in toluene (due to the higher boiling point compared to benzene), to prevent rapid evaporation of the sol fraction.

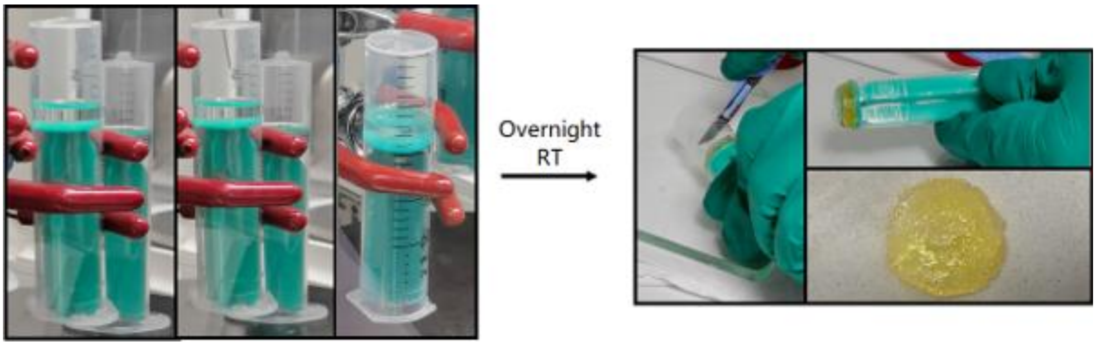

Figure S39: Preparation of the polymeric networks inside syringes.

Rheological characterisation

All tests were performed under air inside a fume-hood, after gels were removed from the inert atmosphere. Oscillatory frequency sweep tests were performed at 0.1% strain for the OX-linked polymer network and at 1% strain for the others. Strain levels were kept low to ensure the materials remained in their linear viscoelastic regions (LVERs) throughout the frequency sweeps. Frequency range was increased from 0.1 rad s^{-1} to 100 rad s^{-1}. Amplitude sweep tests were performed at 10 rad s^{-1} and the amplitude was increased from 0.01% to 400%. Both experiments were performed at 20 °C. Temperature ramp experiments were also performed at 10 rad s^{-1} while keeping the material in LVER. The heating rate was adjusted to be 0.05 °C s^{-1} and the samples were heated from 0 °C to 40 °C. Further increases in temperatures were avoided to prevent the evaporation of toluene from the swollen gels. Creep experiments were performed with an applied stress of 200 Pa over 300 seconds. This was followed by a recovery period of 600 seconds. All the experiments were repeated at least three times and the reported data are the averages of these repeats.

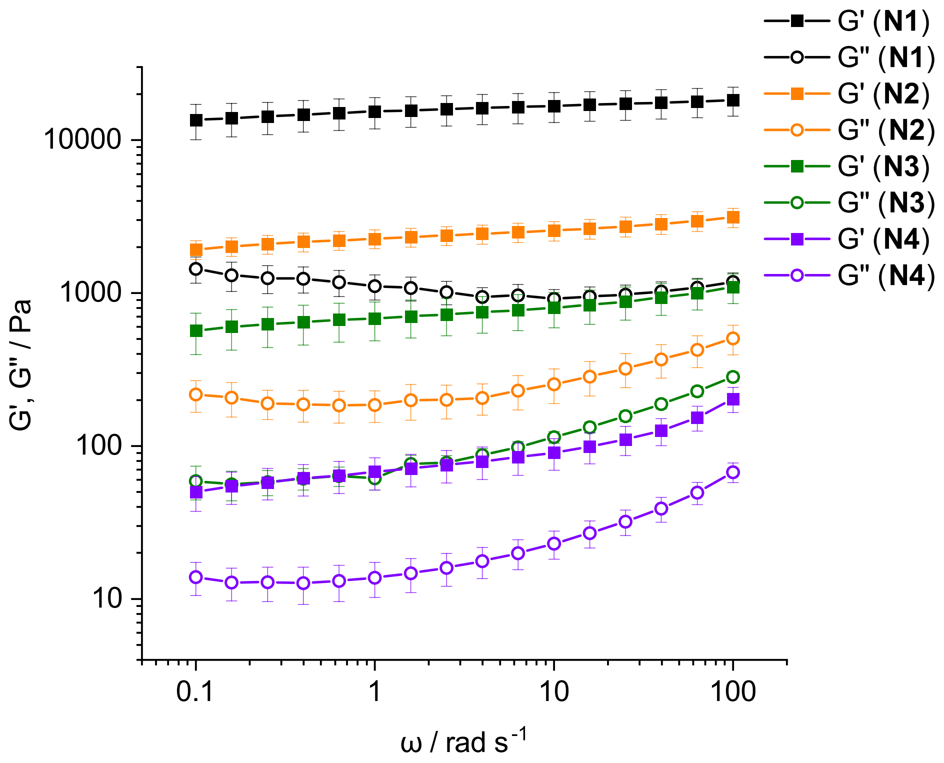

Figure S40: Changes in storage (G’) and loss (G’’) moduli values as a function of angular frequency for the polymer networks prepared with different cyclic substrates.

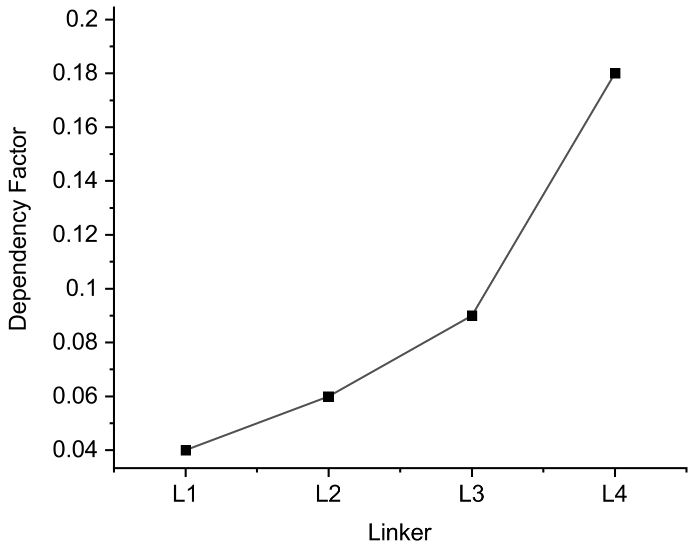

Figure S41: G’ and G’’ values of a covalently crosslinked polymer network is expected to be independent of frequency and any dependency is often attributed to an ‘imperfect’ network structure. Dependency factor/index (x) was calculated using by fitting the G’ data in Figure S40 and fitting it into the power law relationship G’ ~ ω^x, where x = 0 for a perfect polymer network.

Given the large pore sizes observed in SEM, it was thought that not all B and P sites had reacted to form crosslinks. According to the affine network model, the rubbery plateau (G') of a polymer network is directly related to its crosslink density at a constant temperature - $G \propto vkT$, where v is the crosslink density. From here, it can be stated that the crosslink density is the highest with **N1** which has a G' value of 10500 Pa (Table S4). This also corresponds to the highest number of occupied B/P sites. The number of linked sites then decreases four-fold with **N2**. This trend also continues with the **N3** and **N4**. Overall, the number of unreacted P/B units increases as the crosslink density decreases.

Table S4: Approximate G' value for each polymer network, extracted from the frequency sweep plots.

Polymer Network	G' (Approximate) / Pa
**N1**	10500
**N2**	2000
**N3**	800
**N4**	80

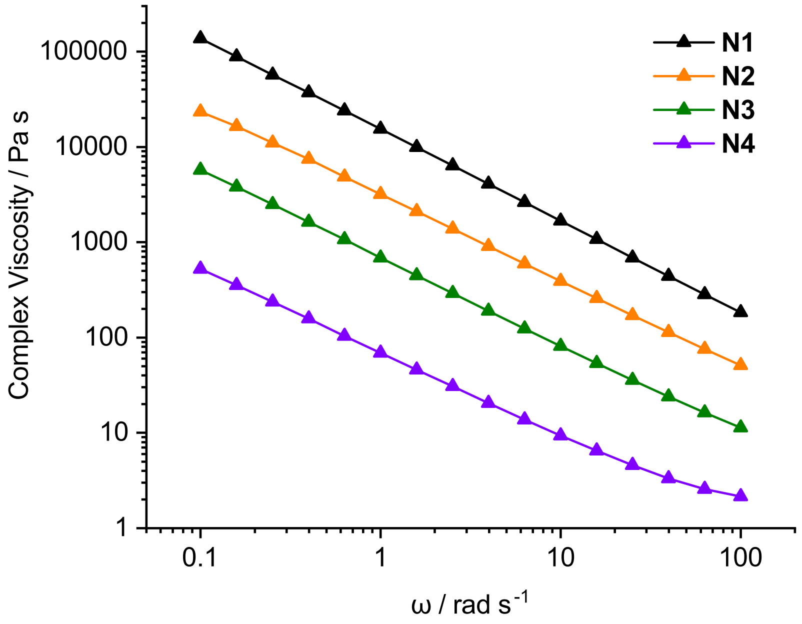

Figure S42: Changes in the complex viscosity of the polymer networks with increasing angular frequency. All the polymer networks exhibited shear-thinning behaviors with increased frequencies.

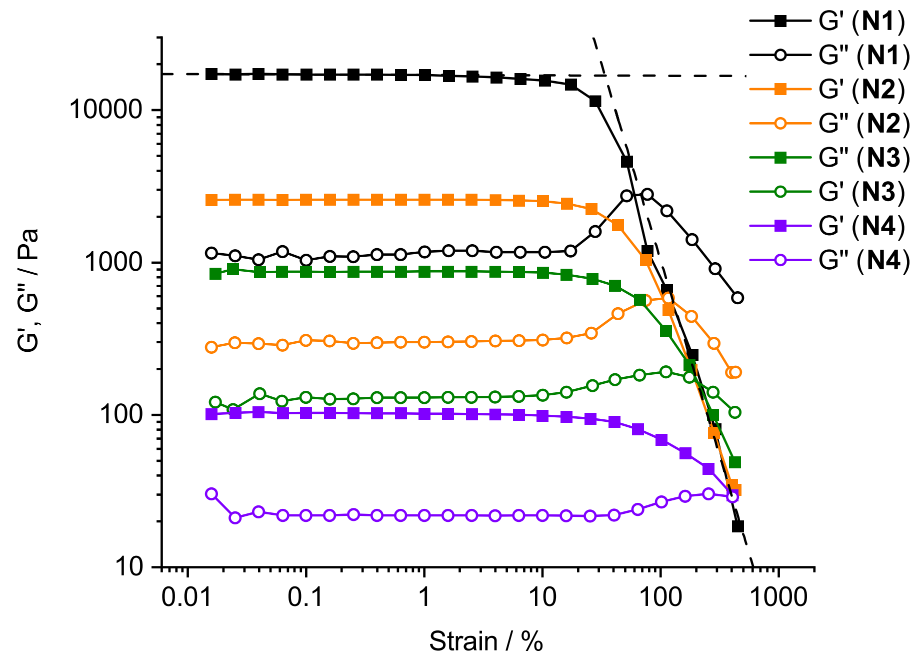

Figure S43: Amplitude sweep test results obtained with the polymer networks. Yield strain values were determined using the crossover strain point between the two fitted linear lines on G'.

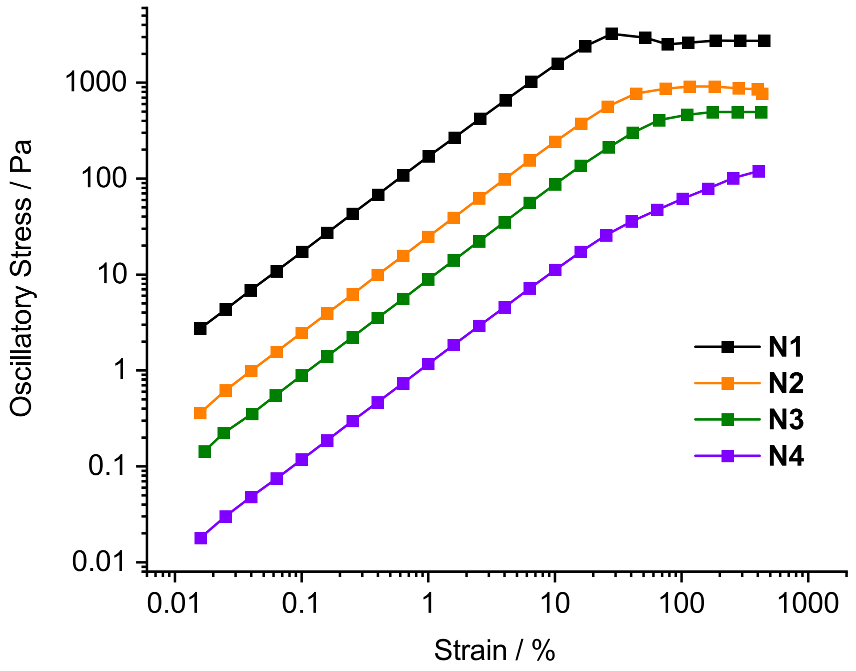

Figure S44: Changes in shear stress as a function of strain for the polymeric FLP networks.

Table S5: Yield strain (γ_{yield}), yield stress (τ_{yield}) and flow point (γ_{flow}) values obtained from Figure S43 and Figure S44.

Network	γ_{yield} (%)	τ_{yield} (Pa)	γ_{flow} (%)	γ_{flow} / γ_{yield}
**N1**	28	3200	55	1.96
**N2**	52	810	100	1.92
**N3**	73	410	180	2.47
**N4**	98	62	400	4.08

Ratio of flow strain (G'=G'') to yield strain were also calculated and it was found that **N1** and **N2** have a greater tendency to brittle fracturing (~2) than **N3** (~2.5) and **N4** (~4).

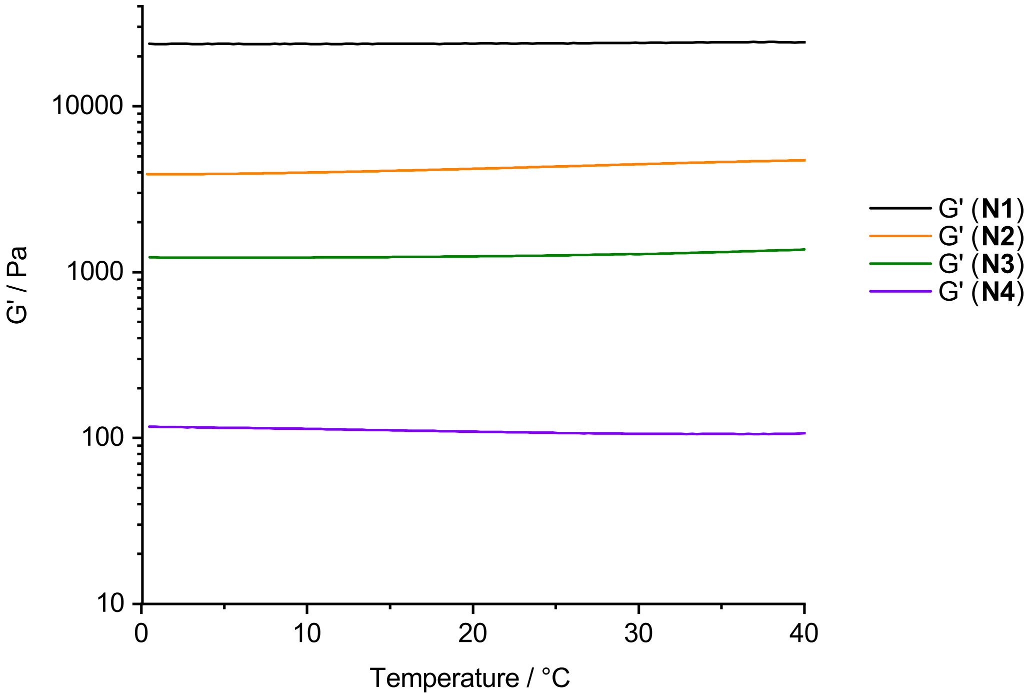

Figure S45: Temperature dependency of the prepared polymeric networks over the temperature range 0 °C - 40 °C.

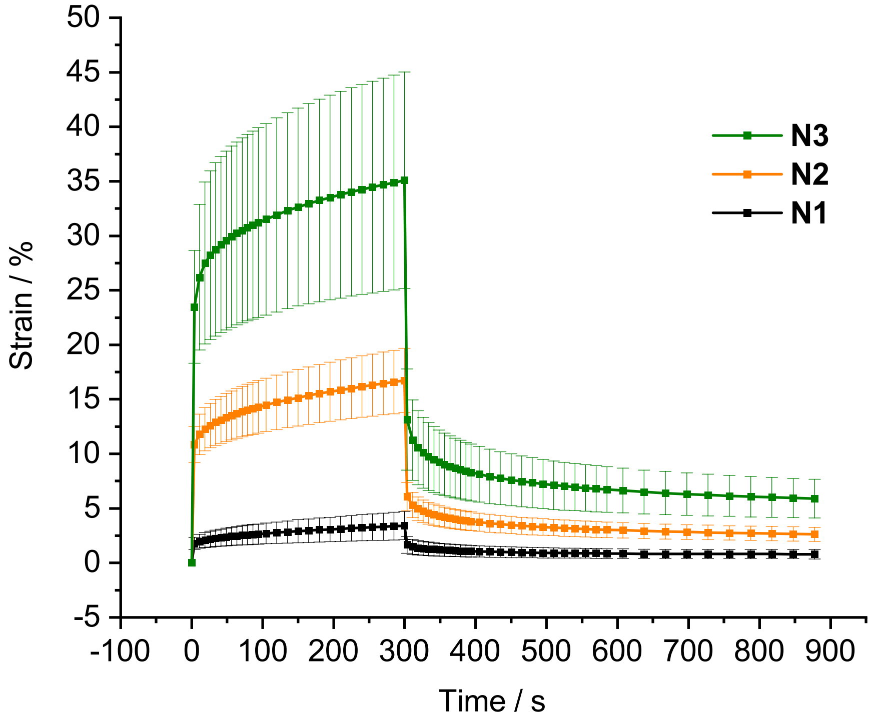

Figure S46: Creep and recovery behaviours of the polymeric FLP gels formed with **L1**-**3** cyclic substrates.

Obtained creep curves were fitted using the Burgers model (Figures S33 and S34, Equation S2) to quantitatively analyze the data.[11] The highest elastic and viscous components were calculated for **N1** (Table S4), confirming the strong resistance of the macromolecular chains to slipping and stretching in this polymer network.

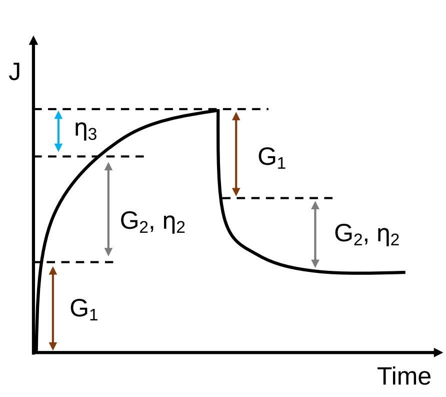

Figure S47: Illustration of the four-parameter Kelvin-Voigt model, also known as the Burgers model.[11-13] In this model, compliance, J, is used instead of strain, which is defined as strain divided by applied stress, as a stress-independent parameter. G and η are the elastic and viscous components, respectively.

Equation S2: Definition of the Burgers creep model, where λ_2 is the retardation time defined as $\lambda_2 = \eta_2 / G_2$.[14]

$$J(t) = \frac{1}{G_1} + \frac{1}{G_2}\left(1 - \exp\left(-\frac{t}{\lambda_2}\right)\right) + \frac{t}{\eta_3}$$

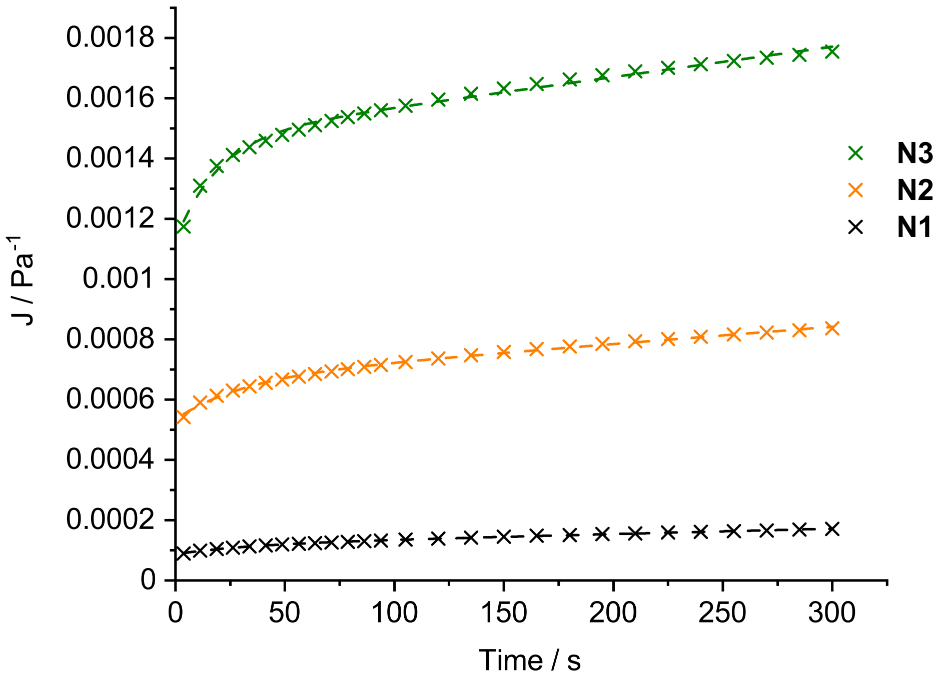

Figure S48: Changes in compliance values as a function of time and fittings obtained using Equation S2 (R^2 = 0.99 in all cases).

Table S6: Best-fitting parameters extracted from the definition of the Burgers model shown in Equation S2.

Network	G_1 / kPa	G_2 / kPa	η_2 (kPa s)	λ_2 (s)	η_3 (kPa s)
**N1**	11.48 ± 0.11	32.22 ± 1.02	1112 ± 90	34.52 ± 2.58	5595 ± 134
**N2**	1.87 ± 0.02	7.36 ± 0.27	232 ± 21	32.88 ± 2.85	1752 ± 63
**N3**	0.77 ± 0.01	2.92 ± 0.12	56 ± 6	19.26 ± 1.73	994 ± 38

Degradation of the Polymer Networks N1 and N2

Exposure to Boron Trichloride

16 mg **N1** xerogel was weighed and transferred into a vial under inert atmosphere at ambient temperature. It was then added into 4 ml of 1 M BCl_3 in DCM and its quick degradation was observed by measuring the swelling ratio as shown below in Figure S50. The same degradation experiment was also repeated with **N2**. 39 mg **N2** xerogel was weighted and transferred into a vial under inert atmosphere at ambient temperature. It was then added into 4 ml of 1 M BCl_3 in DCM and the changes in the xerogel were observed, as shown in Figure S49 and Figure S50.

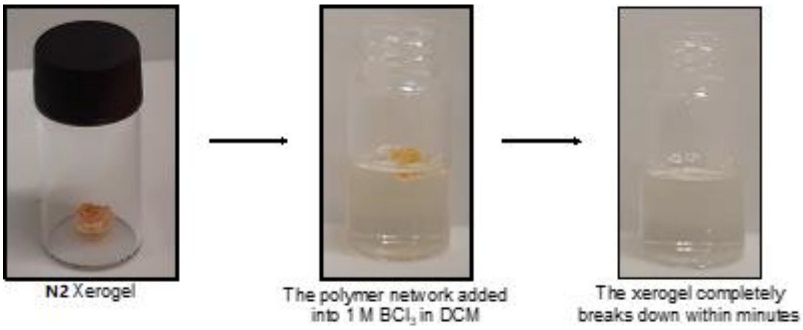

Figure S49: Degradation of **N2** in BCl_3. Also see video evidence Movie S3.

Changes in Xerogel Mass During Degradation

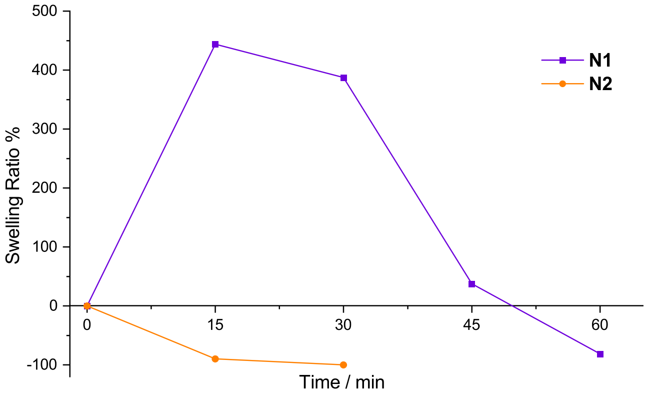

Figure S50: Degradation of **N1** and **N2** in the presence of BCl_3.

GPC Characterization

The supernatant during the degradation experiments was collected, vacuumed down and the resultant solid was characterized by both GPC and NMR spectroscopy. GPC traces shown below indicated the polymer gel had degraded into polymeric species that have comparable molecular weights compared to the parent polymers.

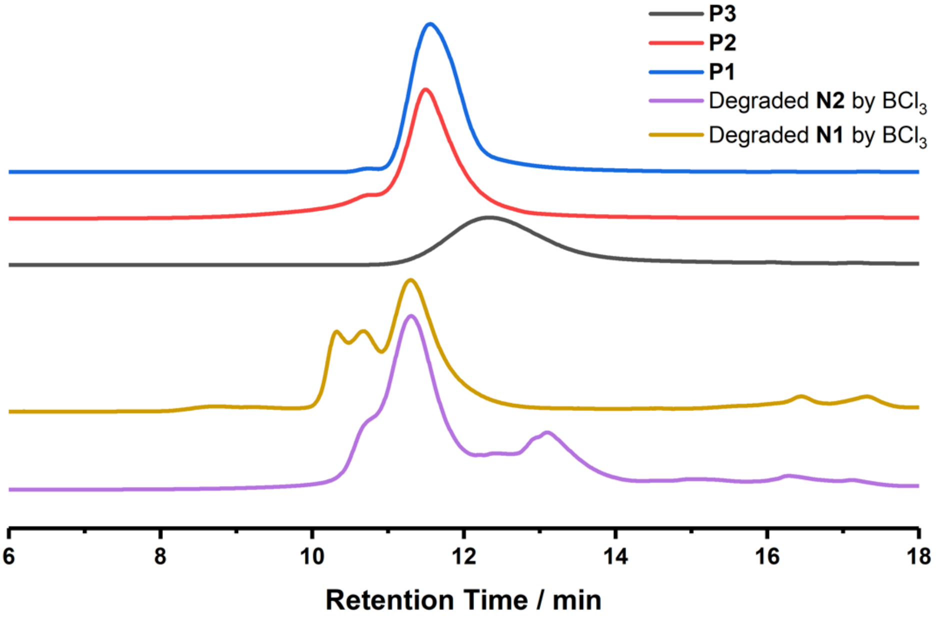

Figure S51: A comparison of the GPC traces of **P1**, **P2**, **P3** and the degradation products of **N1** and **N2** in BCl_3.

Small Molecule Studies

To better understand the degradation process, similar studies were performed with **SM1** and **SM2** and BCl_3 as outlined below.

SM1 (40 mg, 50 µmol) was dissolved in deuterated DCM (0.5 ml) inside a Youngs' tap NMR tube. 1 equivalent of BCl_3 (1 M in DCM, 50 µl, 50 µmol) was subsequently added at RT.

SM2 (35 mg, 46µmol) was dissolved in deuterated DCM (0.5 ml) inside a Youngs' tap NMR tube. 1 equivalent of BCl_3 (1 M in DCM, 46 µl, 46 µmol) was subsequently added and the solution mixed at RT.

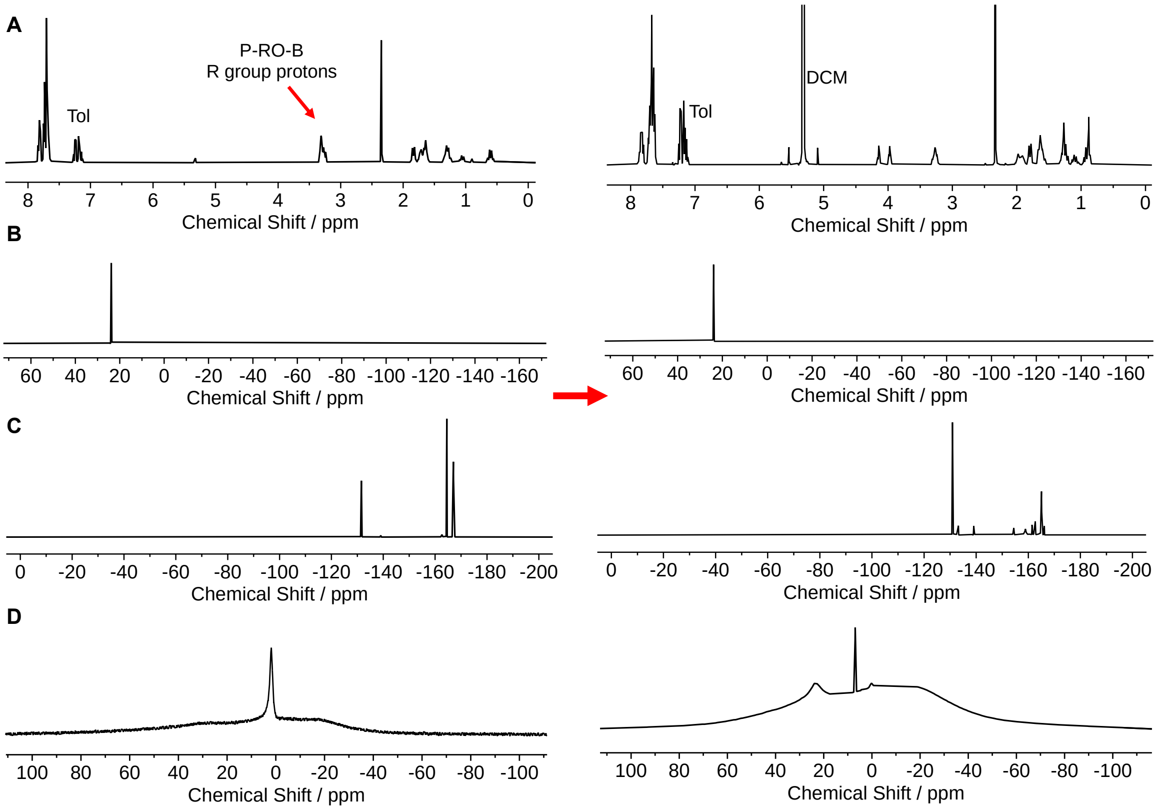

Figure S52: ^{1}H (**A**, 400 MHz), ^{31}P (**B**, 162 MHz), ^{19}F (**C**, 376 MHz) and ^{11}B (**D**, 128 MHz) NMR spectra for the small molecule studies performed by mixing **SM1** with 1 equivalent BCl_3.

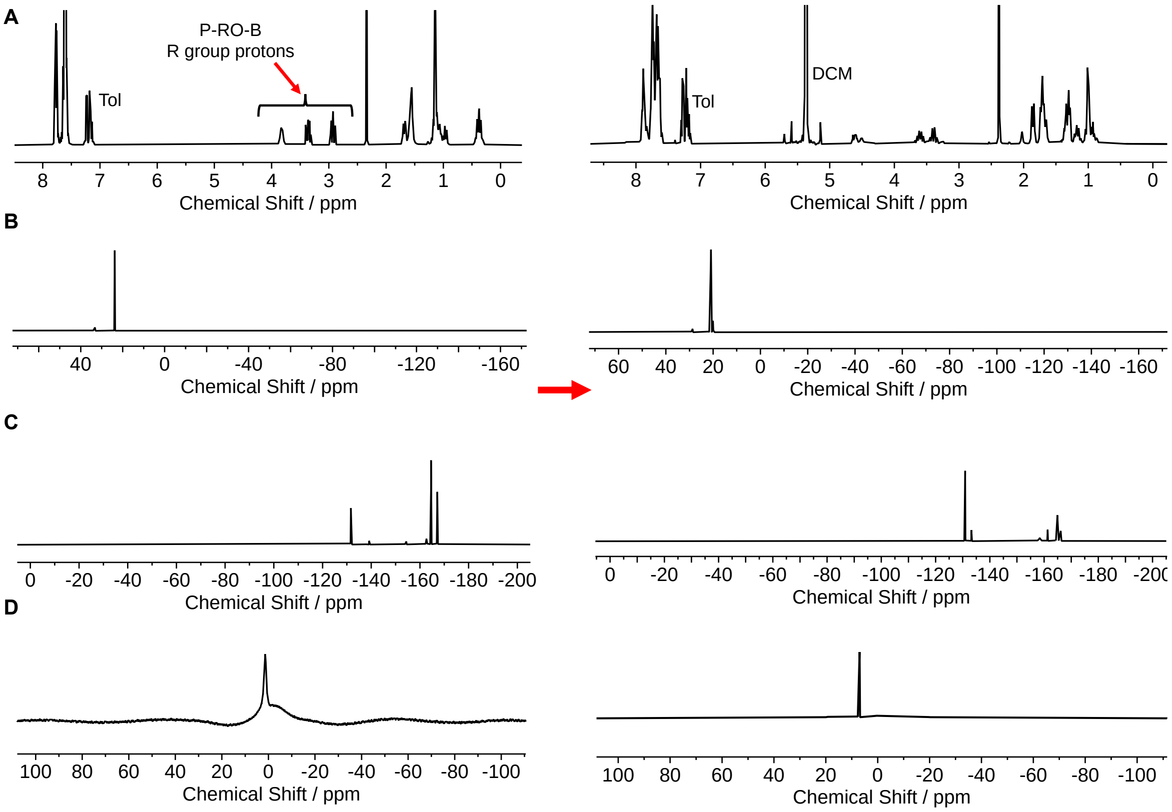

Figure S53: ^{1}H (**A**, 400 MHz), ^{31}P (**B**, 162 MHz), ^{19}F (**C**, 376 MHz) and ^{11}B (**D**, 128 MHz) NMR spectra for the small molecule studies performed by mixing **SM2** with 1 equivalent BCl_3.

The results show that a tetracoordinate phosphine center is maintained when considering ^{31}P NMR spectra, however both the ^{11}B and ^{19}F NMR spectra indicate substantial environment changes. The initial boron resonances (1.77 (**SM1**) and 1.38 ppm (**SM2**)) disappeared, along with generation of another boron species. One principal peak can be seen at 6 ppm, which is believed to be the tetra-coordinated BCl_3, suggesting displacement of the incumbent **1**, which is then subject to other degradation processes. The protons arising from the opened ether moieties are also shifted further downfield, reinforcing this theory since a more de-shielded environment could occur as a result of BCl_3 displacing **1**.

Exposure to Pyridine

0.1 g partially swollen **N1** was transferred to a vial under air and submerged in pyridine at ambient temperature. The polymer gel was left undisturbed for 3 weeks. At the end of this period, the network structure was completely broken down and the polymers had dissolved in pyridine.

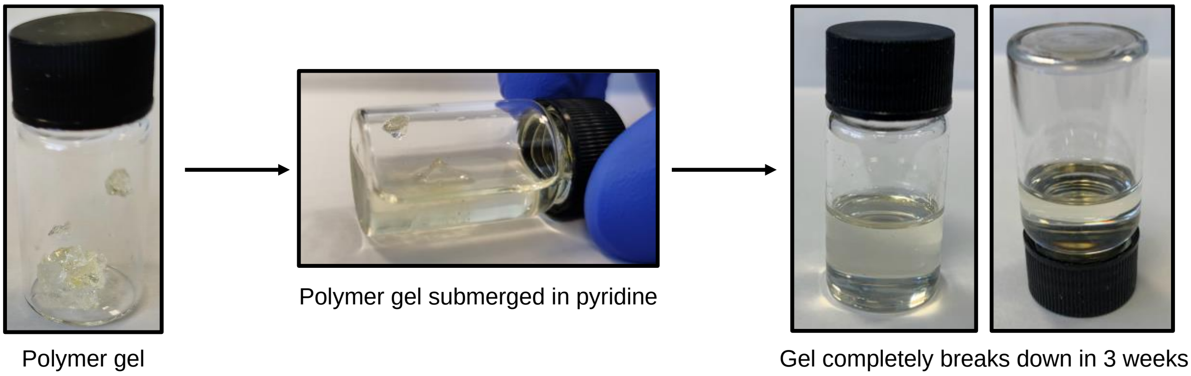

Figure S54: Degradation of **N1** in pyridine.

In addition, the swelling ratio of N2 using pyridine was also measured to investigate the degradation process: 36 mg **N2** xerogel was weighted and transferred into a vial under inert atmosphere at ambient temperature. It was then added into 4 ml anhydrous pyridine and the changes in the swelling ratio of the xerogel was recorded.

Changes in Xerogel Mass During Degradation

When the xerogel was solvated by pyridine, an increase in the weight can initially be observed. After this initial swelling, the mass starts to decrease as a result of degradation. Degradation of the xerogels with pyridine was slow when compared to BCl_3. For the first 7200 min (5 days) the swelling ratio of the gels only decreased to 357%. However, when this pyridine pre-soaked gel was shortly exposed to air, it degraded quickly within ~30 mins. As a control experiment, toluene immersed gel with exposure to air was also performed and no degradation was observed, indicating both pyridine and moisture are key factors of degradation.

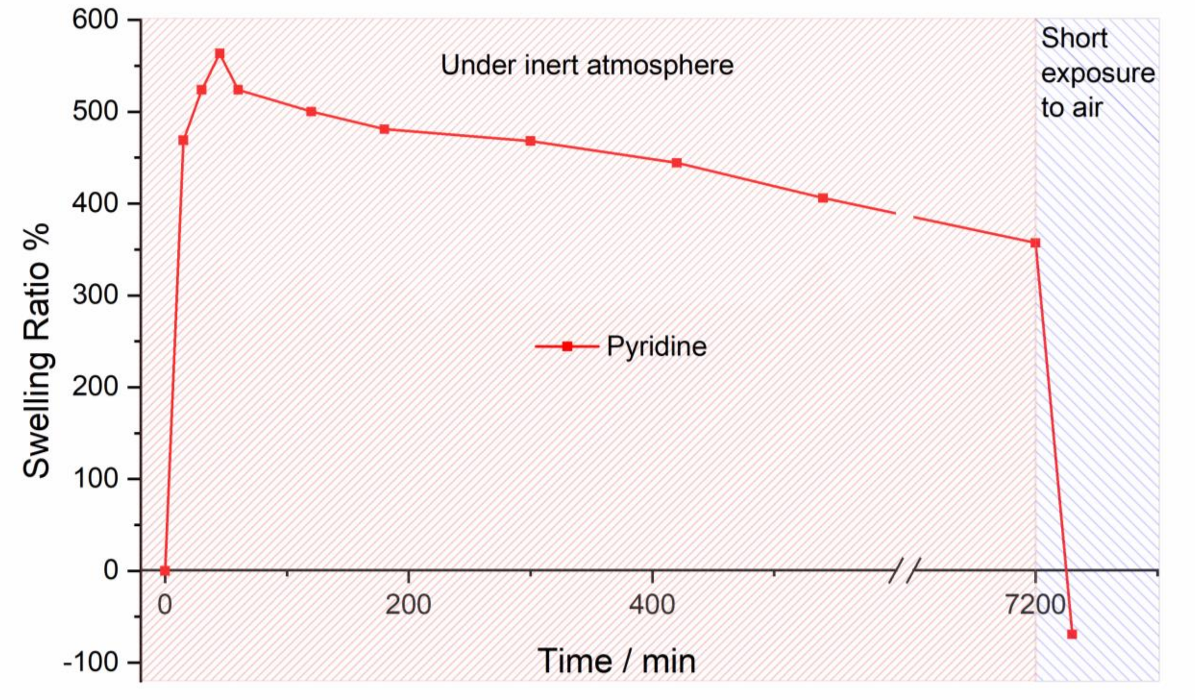

Figure S55: Degradation of **N2** xerogel when submerged in pyridine.

GPC Characterization

The supernatant of the pyridine degradation experiments were collected and vacuumed down. The residue was characterised again using GPC.

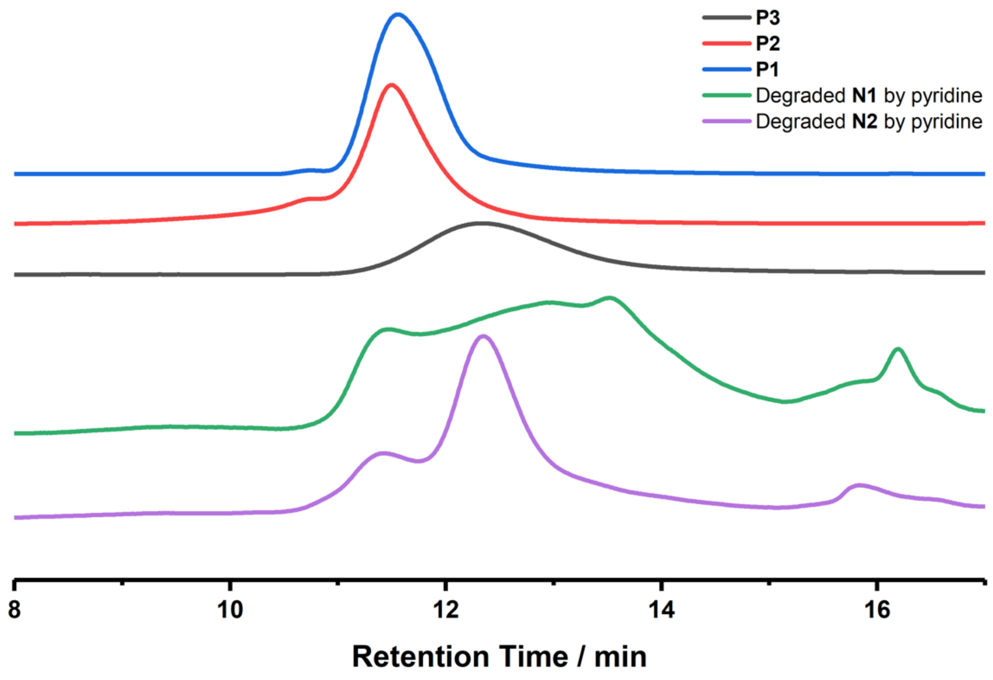

Figure S56: A comparison of the GPC traces of **P1**, **P2**, **P3** and the degradation products of **N1** and **N2** in pyridine.

SM1 (40 mg, 50 µmol) was dissolved in deuterated DCM (0.5 ml) inside a Youngs' tap NMR tube. Anhydrous pyridine (39 µl, 50 µmol) was subsequently added at RT.

SM2 (35 mg, 46µmol) was dissolved in deuterated DCM (0.5 ml) inside a Youngs' tap NMR tube. Anhydrous pyridine (35 µl, 50 µmol) was subsequently added at RT.

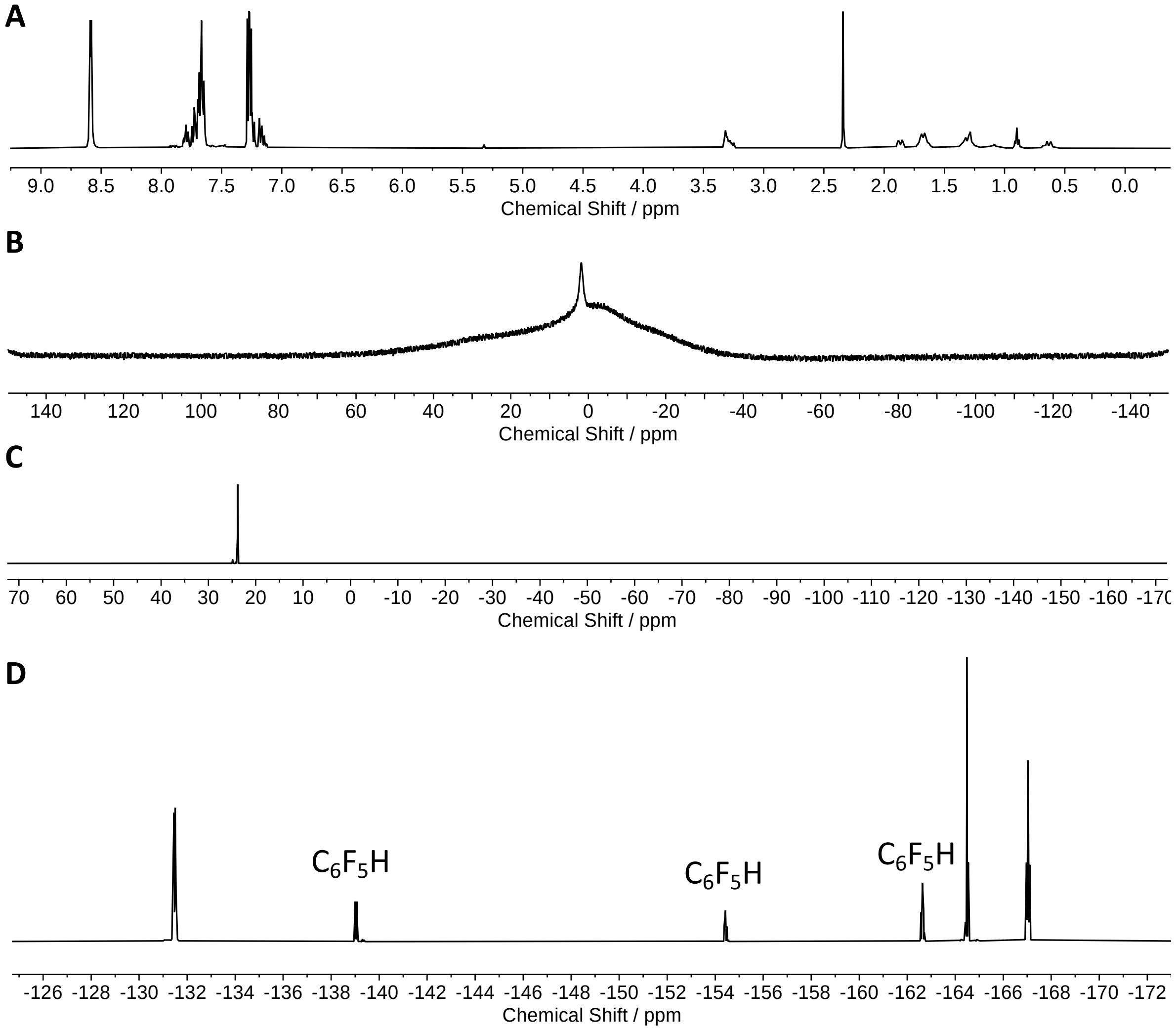

Figure S57: ^{1}H (**A**, 400 MHz), ^{11}B (**B**, 128 MHz), ^{31}P (**C**, 162 MHz), ^{19}F (**D**, 376 MHz) and NMR spectra for the small molecule studies performed by mixing **SM1** with 1 equivalent pyridine. The NMR spectra obtained 24 hours after mixing the starting materials.

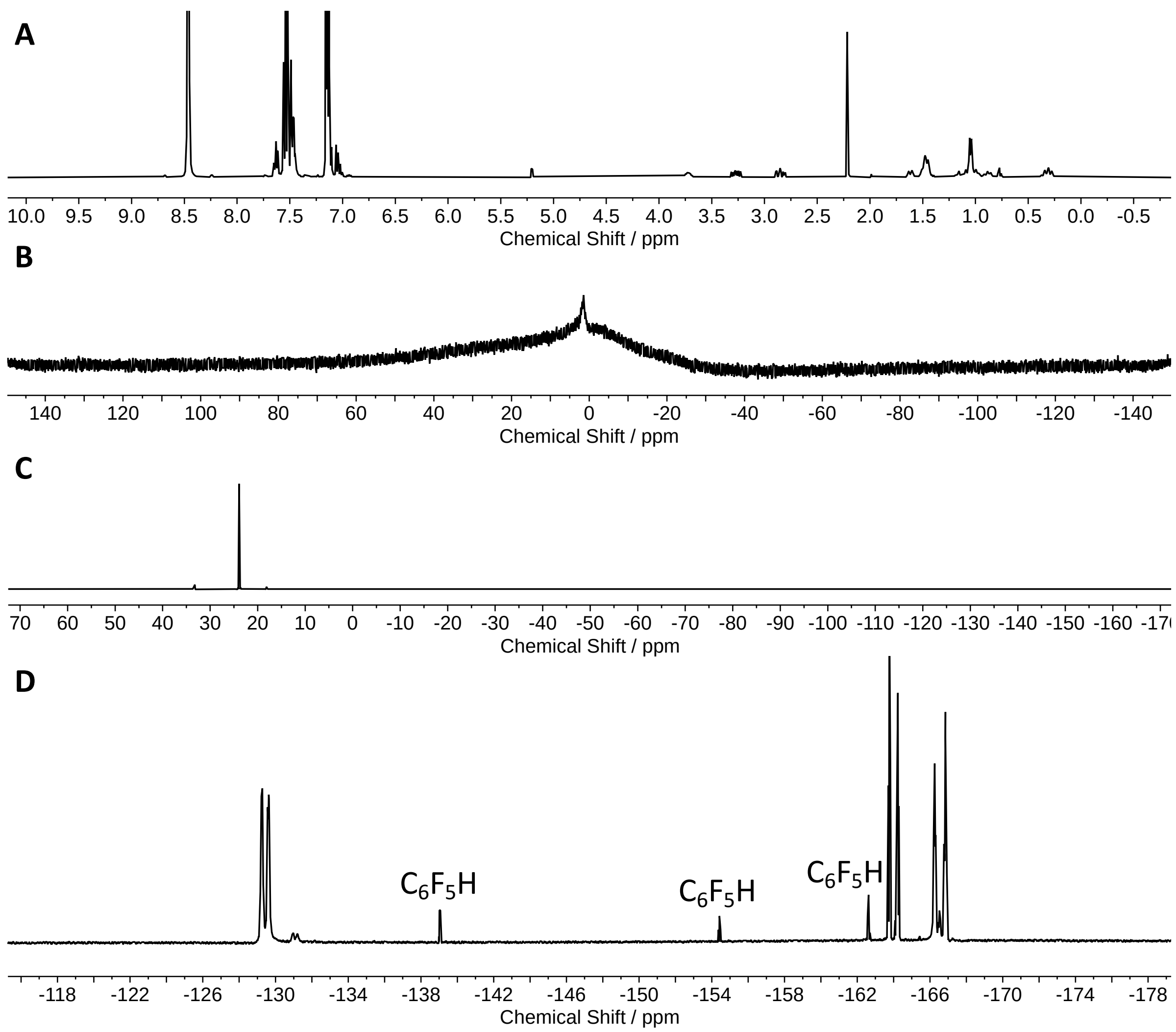

Figure S58: ^{1}H (**A**, 400 MHz), ^{11}B (**B**, 128 MHz), ^{31}P (**C**, 162 MHz), ^{19}F (**D**, 376 MHz) and NMR spectra for the small molecule studies performed by mixing **SM2** with 1 equivalent pyridine. The NMR spectra obtained 24 hours after mixing the starting materials.

In both cases, small molecule studies showed the formation of C_6F_5H species in the ^{19}F NMR spectra, this is commonly attributed to protodeborylation reactions. Given that the degradation of the xerogel **N2** sped up upon exposure to moisture, protodeborylation is understood to participate in the degradation of the cyclic ether-linked poly(FLP) gels in pyridine.

Poly(FLP)s as Functional Materials

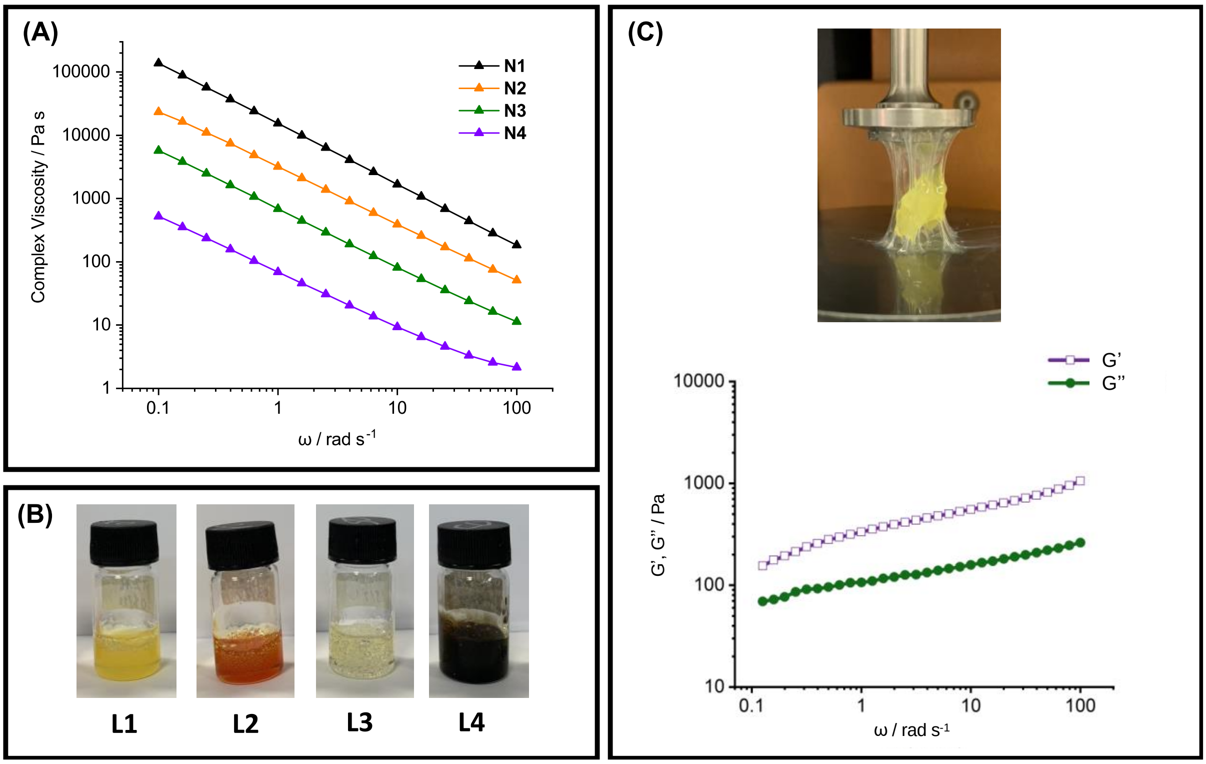

Figure S59: Potential applications of poly(FLP)s as chemical sensors (**A** and **B**) and adhesives **(C)**. **(A)** shows the obtained viscosity profiles when using different linker molecules and **(B)** shows the color changes when 10 equivalents of linker molecules were used. Investigations are ongoing in our laboratory to further understand the link between the colors and susbtrates. **(C)** demonstrates the adhesiveness of the gels when we purposefully prepared softer gels (<1000 Pa) using 4 mol% crosslink density poly(FLP) system with **L1** as the linker.

References

1. Parks, D. J.; Piers, W. E.; Yap, G. P. A., Synthesis, Properties, and Hydroboration Activity of the Highly Electrophilic Borane Bis(pentafluorophenyl)borane, HB(C6F5)21. *Organometallics* **1998,** *17* (25), 5492-5503.

2. Wang, M.; Nudelman, F.; Matthes, R. R.; Shaver, M. P., Frustrated Lewis Pair Polymers as Responsive Self-Healing Gels. *Journal of the American Chemical Society* **2017,** *139* (40), 14232-14236.

3. Williamson, D. T.; Buchanan, T. D.; Elkins, C. L.; Long, T. E., Synthesis of Block Copolymers Based on the Alternating Anionic Copolymerization of Styrene and 1,3-Cyclohexadiene. *Macromolecules* **2004,** *37* (12), 4505-4511.

4. Peuser, I.; Neu, R. C.; Zhao, X.; Ulrich, M.; Schirmer, B.; Tannert, J. A.; Kehr, G.; Fröhlich, R.; Grimme, S.; Erker, G.; Stephan, D. W., CO2 and Formate Complexes of Phosphine/Borane Frustrated Lewis Pairs. *Chemistry – A European Journal* **2011,** *17* (35), 9640-9650.

5. Krachko, T.; Nicolas, E.; Ehlers, A. W.; Nieger, M.; Slootweg, J. C., Ring-opening of Epoxides Mediated by Frustrated Lewis Pairs. *Chemistry – A European Journal* **2018,** *24* (48), 12669-12677.

6. Birkmann, B.; Voss, T.; Geier, S. J.; Ullrich, M.; Kehr, G.; Erker, G.; Stephan, D. W., Frustrated Lewis Pairs and Ring-Opening of THF, Dioxane, and Thioxane. *Organometallics* **2010,** *29* (21), 5310-5319.

7. Zhao, X.; Lough, A. J.; Stephan, D. W., Synthesis and Reactivity of Alkynyl-Linked Phosphonium Borates. *Chemistry – A European Journal* **2011,** *17* (24), 6731-6743.

8. Welch, G. C.; Prieto, R.; Dureen, M. A.; Lough, A. J.; Labeodan, O. A.; Höltrichter-Rössmann, T.; Stephan, D. W., Reactions of phosphines with electron deficient boranes. *Dalton Transactions* **2009,** (9), 1559-1570.

9. Kühl, O., The Range of Chemical Shifts, Coupling Constants, and What Influences Each. In *Phosphorus-31 NMR Spectroscopy: A Concise Introduction for the Synthetic Organic and Organometallic Chemist*, Kühl, O., Ed. Springer Berlin Heidelberg: Berlin, Heidelberg, 2008; pp 7-23.

10. Beckett, M. A.; Strickland, G. C.; Holland, J. R.; Sukumar Varma, K., A convenient n.m.r. method for the measurement of Lewis acidity at boron centres: correlation of reaction rates of Lewis acid initiated epoxide polymerizations with Lewis acidity. *Polymer* **1996,** *37* (20), 4629-4631.

11. Alfrey, T.; Gurnee, E. F., CHAPTER 11 - DYNAMICS OF VISCOELASTIC BEHAVIOR. In *Rheology*, Eirich, F. R., Ed. Academic Press: 1956; pp 387-429.

12. Cuomo, F.; Cofelice, M.; Lopez, F., Rheological Characterization of Hydrogels from Alginate-Based Nanodispersion. *Polymers* **2019,** *11* (2).

13. Suriano, R.; Griffini, G.; Chiari, M.; Levi, M.; Turri, S., Rheological and mechanical behavior of polyacrylamide hydrogels chemically crosslinked with allyl agarose for two-dimensional gel electrophoresis. *Journal of the Mechanical Behavior of Biomedical Materials* **2014,** *30*, 339-346.

14. Abdurrahmanoglu, S.; Okay, O., Rheological behavior of polymer–clay nanocomposite hydrogels: Effect of nanoscale interactions. *Journal of Applied Polymer Science* **2010,** *116* (4), 2328-2335.
